# Ultrafast Electron Transfer from CuInS_2_ Quantum Dots to a Molecular Catalyst for Hydrogen Production: Challenging Diffusion Limitations

**DOI:** 10.1021/acscatal.3c06216

**Published:** 2024-03-04

**Authors:** Andrew J. Bagnall, Nora Eliasson, Sofie Hansson, Murielle Chavarot-Kerlidou, Vincent Artero, Haining Tian, Leif Hammarström

**Affiliations:** † Department of Chemistry-Ångström Laboratory, Uppsala University, SE-75120 Uppsala, Sweden; ‡ Univ. Grenoble Alpes, CNRS, CEA, IRIG, Laboratoire de Chimie et Biologie des Métaux, 17 rue des Martyrs, F-38054 Grenoble, Cedex, France

**Keywords:** hydrogen, photocatalysis, copper indium sulfide, quantum dots, molecular catalyst, transient absorption, artificial photosynthesis

## Abstract

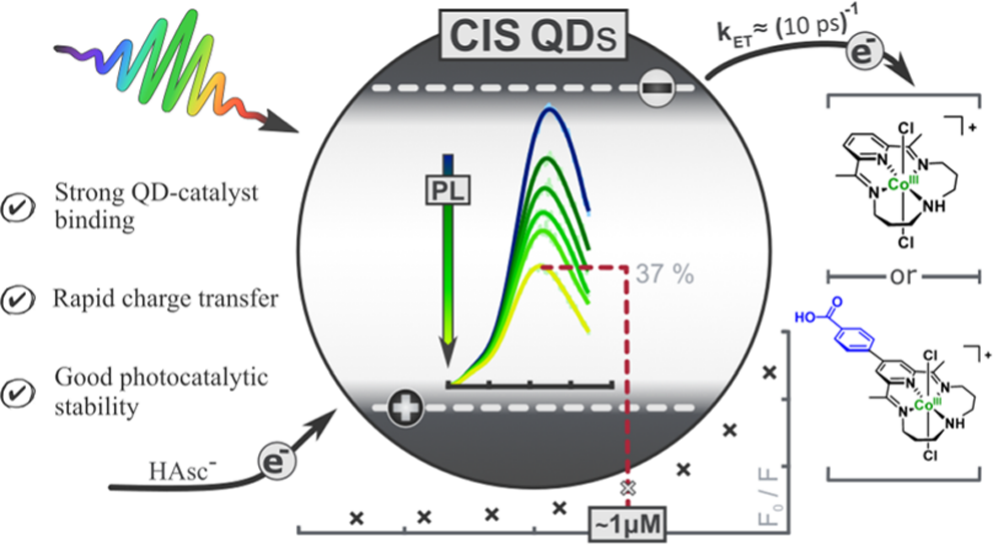

Systems integrating quantum dots with molecular catalysts are attracting ever more attention, primarily owing to their tunability and notable photocatalytic activity in the context of the hydrogen evolution reaction (HER) and CO_2_ reduction reaction (CO_2_RR). CuInS_2_ (CIS) quantum dots (QDs) are effective photoreductants, having relatively high-energy conduction bands, but their electronic structure and defect states often lead to poor performance, prompting many researchers to employ them with a core–shell structure. Molecular cobalt HER catalysts, on the other hand, often suffer from poor stability. Here, we have combined CIS QDs, surface-passivated with l-cysteine and iodide from a water-based synthesis, with two tetraazamacrocyclic cobalt complexes to realize systems which demonstrate high turnover numbers for the HER (up to >8000 per catalyst), using ascorbate as the sacrificial electron donor at pH = 4.5. Photoluminescence intensity and lifetime quenching data indicated a large degree of binding of the catalysts to the QDs, even with only ca. 1 μM each of QDs and catalysts, linked to an entirely static quenching mechanism. The data was fitted with a Poissonian distribution of catalyst molecules over the QDs, from which the concentration of QDs could be evaluated. No important difference in either quenching or photocatalysis was observed between catalysts with and without the carboxylate as a potential anchoring group. Femtosecond transient absorption spectroscopy confirmed ultrafast interfacial electron transfer from the QDs and the formation of the singly reduced catalyst (Co^II^ state) for both complexes, with an average electron transfer rate constant of <*k*_ET_> ≈ (10 ps)^−1^. These favorable results confirm that the core tetraazamacrocyclic cobalt complex is remarkably stable under photocatalytic conditions and that CIS QDs without inorganic shell structures for passivation can act as effective photosensitizers, while their smaller size makes them suitable for application in the sensitization of, *inter alia*, mesoporous electrodes.

## Introduction

In recent years, there has been a growing interest in combining the strong and independently tunable light harvesting properties of colloidal quantum dots (QDs) with the potentially fast and selective catalysis provided by molecular catalysts via interfacial electron transfer (ET), allowing the precise making and breaking of chemical bonds from photogenerated charge carriers with sufficient potentials.^1−8^ Some advantages of QD photosensitizers (PS) over molecular dyes are their exceeding photostability, long exciton lifetimes, and their broad absorption spectral coverage across the solar spectrum; these are essential properties in the design of efficient photocatalytic systems. Within the broader perspective, the discovery and development of QDs was recognized with the 2023 Nobel Prize in Chemistry awarded to Bawendi, Brus, and Jekimov.^9^

Ternary I–III–VI metal chalcogenide QDs, such as AgInS_2_ (AIS) and, in particular, CuInS_2_ (CIS) QDs, have attracted increasing attention in the past decade as alternatives to the commonly used Cd and Pb binary chalcogenides.^10−12^ Their high-energy conduction bands (CBs) are beneficial for reductive photochemistry, for example, to drive the hydrogen evolution reaction (HER) and CO_2_ reduction reaction (CO_2_RR; for convenience and in line with the literature, we keep the band nomenclature of bulk semiconductors for the CIS QDs). As a result of their composition, the ternary QDs often carry a large number of lattice imperfections, with potential fluctuations that result in carrier localization and complex photophysical behavior. Moreover, neat CIS QDs and nanorods have been reported to show limited photocatalytic H_2_ performance even with added cocatalysts, and CIS/ZnS core–shell structures have often been preferred for photocatalytic reactions.^3,13−15^ However, the tolerance of CIS QDs to off-stoichiometry offers additional degrees of freedom to tune photophysical and electronic properties. In CIS, higher photocatalytic activities (toward the HER) have been reported for Cu-deficient structures, attributed to more efficient hole transfer, provided by the lower valence band (VB)-edge, despite accelerated electron trapping rates,^14,16^ and even neat, Cu-deficient CIS QDs showed high H_2_ production activities with simple metal salts as cocatalysts.^16^

Molecular catalysts, on the other hand, represent an interesting strategy for enhancing the catalytic activity of more Earth-abundant first-row transition metals.^17^ Often inspired by biology, the coordination sphere around the metal can be tuned to improve the activity, selectivity, and stability in catalyzing the desired reaction, and anchoring groups can be included in the molecular structure to enable controlled integration onto materials.^18−20^ One such catalyst is the cobalt tetraazamacrocyclic complex, [Co(N_4_H)Cl_2_]^+^ (**1**), which is an effective and robust, oxygen-tolerant catalyst for the hydrogen evolution reaction (HER) in both organic and aqueous conditions. **1** is reported to display notable activity, efficiency, and stability in both electrocatalytic and photocatalytic conditions.^21−27^ Indeed, this catalyst has been successfully used with a diverse range of photosensitizers, including [Ru^II^(bpy)_3_]^2+^,^27−30^ the triazatriangulenium derivative organic dye (TATA^+^),^31,32^ and CdTe QDs,^33^ as well as in dye–catalyst assembly systems.^34,35^ However, the excellent performances of **1** and its anchorable derivatives with glutathione-capped CIS/ZnS QDs, reported by the Wang and Collomb groups, are particularly noteworthy.^15,36,37^ This combination of PS and molecular catalyst has reported turnover numbers (TONs) of up to 7700 at pH 5.0, with electron transfer (ET) occurring on the time scale of ∼1 ns in the case of an anchored derivative.

The anchoring groups on the catalysts are intended to bind them to the QDs and thus accelerate ET from the QDs by circumventing diffusional limitations that are assumed to hamper photocatalytic efficiencies.^38^ Improvements in photocatalysis TONs or photoluminescence (PL) quenching efficiencies from the addition of anchoring groups have, however, sometimes been smaller than one may expect when transitioning from purely diffusional to static QD–catalyst interactions.^5,36,39,40^ Although this is often not discussed, it may suggest that a fraction of the unmodified catalysts bind to the QDs despite lacking any specific anchoring group. Many cases have indeed been reported where small organic quenchers, as well as transition metal complexes and proteins, bind strongly to QDs, often explained by electrostatic interactions.^3,6,7,41−47^ However, other types of molecule-QD interactions have also been suggested.^8,48,49^ In our previous studies of CIS QDs combined with [Fe_2_(bdt)(CO)_6_]^0^ or [Re(bpy)(CO)_3_Cl]^0^ catalysts, we also observed a strong association of the charge-neutral complex with the QDs in the absence of a specific binding group or other easily recognizable interactions,^39,40^ and similar results were reported for CdSe QDs with [Fe_2_(pdt)(CO)_6_]^0^ (bdt = benzene-1,2-dithioloate; pdt = propane-1,3-dithiolate).^50^ The favorable self-assembly of these QD/molecule combinations is intriguing, and more efforts in understanding and controlling this behavior could benefit applications of QDs in, e.g., photocatalysis.

Moreover, despite the large number of reports on photocatalytic activities in QD-molecular systems and an increasing interest in QDs as photosensitizers for electron and hole acceptors, there are limited spectroscopic studies on ET rates involving molecular catalysts. For the present catalyst, Sandroni et al. reported (at pH 4.5) TONs of 4580 and 900 with respect to **1** and CIS/ZnS QDs, respectively, but no photophysics or ET rates were reported.^15^ Nie et al. introduced a 2′,6′-dicarboxypyridin-4′-yl anchoring group on the macrocyclic ligand of **1** for covalent attachment to the CIS/ZnS QD surface.^36^ They reported TONs of 2670 and 1360 with respect to the functionalized and unfunctionalized **1**, respectively. For the covalently attached **1**, ET rate constants (*k*_ET_) were determined by PL decay kinetics for the Co^III^-to-Co^II^ (0.61 ns, 84%) and Co^II^-to-Co^I^ (0.78 ns, 75%) reduction steps–only twice as fast as for **1** without covalent attachment. They also reported an ET rate constant obtained from femtosecond transient absorption (fs-TA) experiments of *k*_ET_ = (1.2 ns)^−1^ for surface-bound **1**, but it is unclear whether the first or second reduction step was monitored since the absorption band from the reduced catalyst was not observed. Direct observation of reduced/oxidized species by transient absorption spectroscopy (TAS) has many advantages compared to indirect determinations of ET rates relying on changes in the QD signal response in the presence of the acceptor/donor, such as accelerated excitonic bleach recovery dynamics or a lowering of the initial TA signal magnitude upon interfacial transfer of conduction band (CB) electrons, as such observations can reflect changes in the QD-environment interface, which are not directly related to ET. Yet, studies in the literature remain very scarce in this regard; more direct spectroscopic identification of intermediates and products could be beneficial in order to understand the underlying charge separation dynamics following photoexcitation.

**Figure 1:**
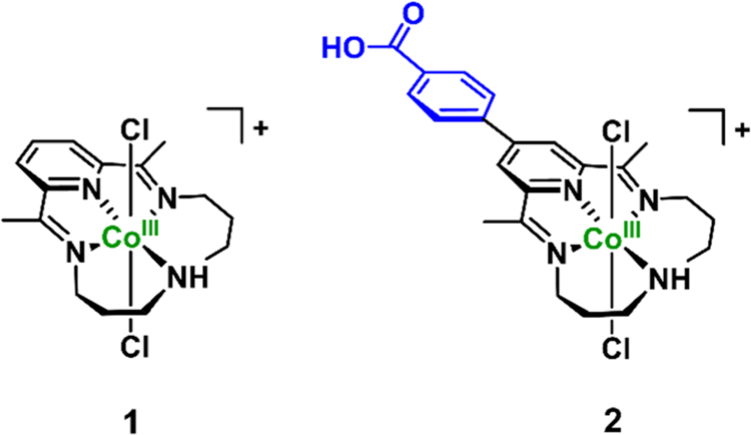
Chemical structures of the molecular catalyst, **1**, and its novel derivative, **2**, functionalized with a benzoic acid moiety (in blue), both in the Co^III^ oxidation state.

Herein, we combine CIS QDs with **1** to construct an efficient hydrogen-evolving system, using ascorbate as the sacrificial donor. Alongside **1**, a novel derivative incorporating a benzoic acid moiety was prepared and investigated: **2** (Figure 1). This moiety may function as an anchoring group to bind the catalyst to certain photosensitizers, analogous to previous studies by Nie et al. and also to similar systems with different catalysts.^36,39^ We investigate the role of the anchoring group in varying conditions through steady-state and time-resolved PL quenching experiments, reporting highly efficient quenching by both catalysts. A static quenching model based on a Poisson distribution of the catalyst molecules bound to the QD surfaces was applied, underscoring the high binding affinities as well as providing an indirect estimation of the QD concentration. Through fs-TA measurements, we provide direct (UV–vis) and indirect (MIR) evidence of ultrafast <*k*_ET_> ≈ (10 ps)^−1^ reduction of **1**, both with and without the anchoring group. This is 2 orders of magnitude faster than reported by Nie et al. using CIS/ZnS.^36^ The results challenge the common view that photocatalytic hydrogen production with QDs and molecular catalysts is limited by diffusional encounter and electron transfer to the catalyst.^38^ The aim of the present study is to increase the understanding of these photocatalytic inorganic–organic hybrid systems by investigating and modeling the factors behind the high TONs reported.

## Results and Discussion

### Quantum Dot Synthesis and Characterization

Earlier reports of ternary QD photosensitizers have largely relied on the use of organic solvents with organic acids as proton sources, and there still remain a number of challenges in producing high-quality QDs in aqueous conditions, which is cheaper, simpler, and greener than producing water-soluble QDs through ligand exchange from organic synthesis.^51,52^ Furthermore, many successful approaches rely on hazardous heavy metals,^53−56^ which are restricted by, e.g., the RoHS directive in the EU and US.^57,58^ Herein, we rely on a facile water-based synthesis of Cu-deficient CIS QDs with a hybrid passivation layer, developed by Huang et al.,^59^ based on a core/shell procedure reported by Chen et al.^51^ The hybrid passivation^60^ layer consists of shorter organic ligands (l-cysteine) and halide anions (iodide), which passivate surface sites inaccessible to the organic ligand. The hybrid passivation layer is introduced during synthesis so that no ligand or solvent exchange is required. The reduced size profile of the l-cysteine-coated particles can be beneficial for their utilization in photoelectrocatalysis (PEC) devices, allowing sensitization of photoelectrode materials which have restrictive pore sizes. This was demonstrated by Huang et al. with CIS-sensitized photocathodes of mesoporous nickel oxide (NiO) printed onto fluorine-doped tin oxide (FTO), used in PEC without the use of sacrificial donors.^59^ Small-sized capping ligands are likely beneficial for the interfacial ET rates when compared to thick insulating layers, which may lower the probability of electron tunneling. Avoiding insulating effects is proposed to have been an important factor behind the sub-ps ET between hybrid-passivated CIS QDs (Cu:In, ∼1:5) and a FeFe-hydrogenase mimic reported in our previous work.^39^

The synthesis resulted in CIS QDs in a chalcopyrite (or zinc blende)^59^ crystal phase according to powder X-ray diffraction (Figure S1). The QDs are Cu-deficient, with an elemental ratio of approximately 1:3.5 copper to indium, as determined by inductively coupled plasma atomic emission spectroscopy (ICP-AES).^39^ The surface ζ-potential of the aqueous CIS QDs was measured to be −30 mV, with a hydrodynamic diameter of ∼3.5 nm, close to the size estimated previously by high-resolution transmission electron microscopy (HRTEM).^59^ This implies electrically stabilized colloids (|ζ| ≥ 30 mV) with a negative net charge density within the slipping plane (i.e., the immobile layer) around the QDs.

**Figure 2:**
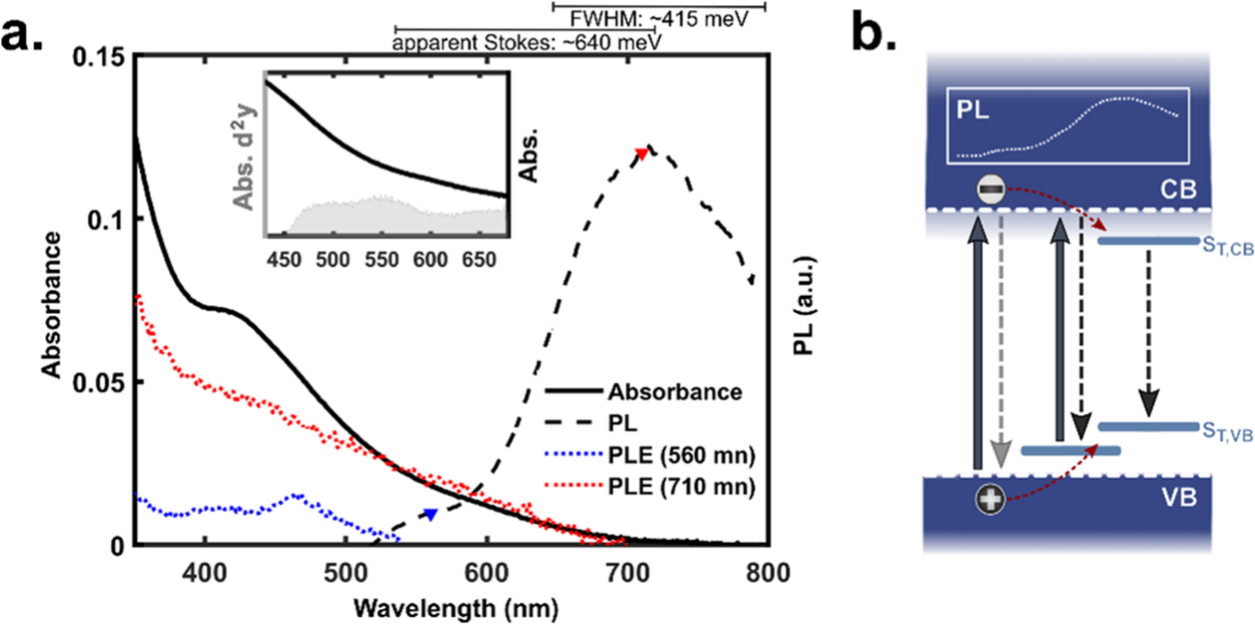
(a) Absorbance, photoluminescence (PL), and photoluminescence excitation (PLE) spectra for a CIS QD sample (ca. 1.3 μM) dispersed in water. The blue/red triangles on the PL spectrum indicate the wavelengths at which the PLE was monitored (560 and 710 nm). The 560 nm PLE is presented with a scale factor of 5:1. Inset: close-up showing absorption spectrum (black) and its second derivative (shaded gray). The second derivative has been smoothed with a moving average filter and scaled for clarity. (b) Schematic diagram of the involved transitions. Absorption is shown in solid arrows and PL in dashed arrows (tentative assignments).

Figure 2 shows representative ensemble absorption and photoluminescence (PL) spectra from the CIS QDs (see Figure S2 for spectra from other batches). The broad and featureless absorption spectrum is typical for the ternary species due to the variable nature of allowed optical transitions. From the second derivative of the absorption spectrum (inset, Figure 2a) and fs-TA measurements (*vide infra*), we estimate an optical bandgap of approximately 2.4 eV (∼520 nm) superimposed on a lower-energy absorption tail from sub-bandgap transitions centered around 2 eV (∼605 nm), presumably dominated by Cu^+^ states in proximity to the VB edge. Jara et al., for example, showed that the tail absorption decreases with increasing Cu deficiency.^61^ Based on our previous photophysical study of CIS QDs with higher Cu deficiency (Cu_0.2_In_1_S_*x*_), we relate the more distinct absorption feature at ∼430 nm to a higher energy transition, which may involve a deeper hole state that obtains more band-edge character if the VB edge is further depleted from Cu states by decreasing Cu content.^39^

The main PL band at ∼710 nm in Figure 2a exhibits a broad bandwidth (fwhm: ∼415 meV), a large apparent Stokes shift (*E*_VB–CB_–*E*_PL,710 nm_: ∼640 meV), and a multiexponential PL decay (Table S1). These PL characteristics are typical for CIS QDs and related ternary systems and are commonly ascribed to the involvement of localized carriers (electrons and/or holes). The PL excitation (PLE) spectrum monitored at the main band (λ_mon._ = 710 nm) includes contributions from excitation across all wavelengths in the absorption band, whereas the PL tail between 500 and 600 nm only results from excitation above the onset of the band-edge transition energy. A tentative assignment consistent with the data (see PL quenching studies) is depicted in Figure 2b. Note that alternative interpretations based on symmetry forbidden exciton transitions and large electron–phonon couplings have also been proposed to account for the low-energy absorption tail and PL in CIS QDs;^62^ we do not exclude that such models could also be valid in our case with high Cu deficiency, but this will not be explored further in this article.

### Photocatalytic Studies

**1** catalyzes the HER by a heterolytic ECEC-type mechanism starting from the Co^II^ state. Under homogeneous organic conditions, this was previously reported to involve the protonation and decoordination of the macrocycle amine at the Co^II^ state, in effect potentially acting as a proton relay, followed by a one-electron reduction before the rate-determining second protonation step.^23^ Under homogeneous aqueous conditions, however, it has also been proposed that the protonation of the macrocycle amine does not necessarily play a role. In either case, it is necessary to reduce the cobalt center to the Co^I^ state in order to initiate HER catalytic turnover via the successive involvement of a Co^III^-hydride and Co^II^-hydride species. In such a mechanism, the as-synthesized Co^III^ complex is a precatalyst for HER.^30^

**Figure 3:**
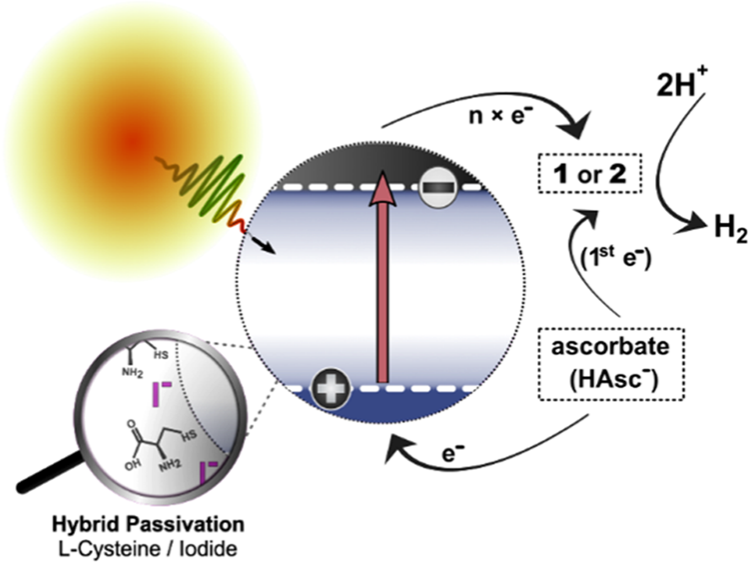
Illustration of the investigated photocatalytic system in aqueous solution with hybrid-passivated CIS QDs as the photosensitizer, **1** or **2** as the molecular HER catalyst, and ascorbate as the sacrificial electron donor.

The photocatalytic system consisted of the hybrid-passivated CIS QDs as the photosensitizer with either **1** or **2** as the catalyst in a solution of 0.5 M ascorbic acid/sodium ascorbate (H_2_Asc/NaHAsc) buffer in water at pH = 4.5 to maintain a constant bulk concentration of protons (Figure 3). Ascorbate also acts as the sacrificial electron donor (or QD hole scavenger), replacing the electrons donated to the catalyst by the photoexcited QDs and thereby mitigating electron–hole recombination processes.^36^

These conditions for pH and buffer concentration were already previously established as appropriate and effective for the closely related CIS/ZnS QDs with **1** in the literature.^15,36^ A solution pH of 4.5 resulted in higher initial TOFs than pH = 5.0 or pH = 5.5, although similar behavior with somewhat higher lifetime TONs per QD and catalyst were reported at the latter pH values. The catalyst system is known to be active in this pH range and reported to be stable down to pH = 2 or below.^22,25^ More acidic conditions at pHs below the p*K*_a1_ of ascorbic acid should sharply reduce the concentration of ascorbate, as per the Henderson–Hasselbalch equation,^63^ so pH = 4.5 represents a good balance of sufficient proton concentration for the HER and system stability.

**Figure 4:**
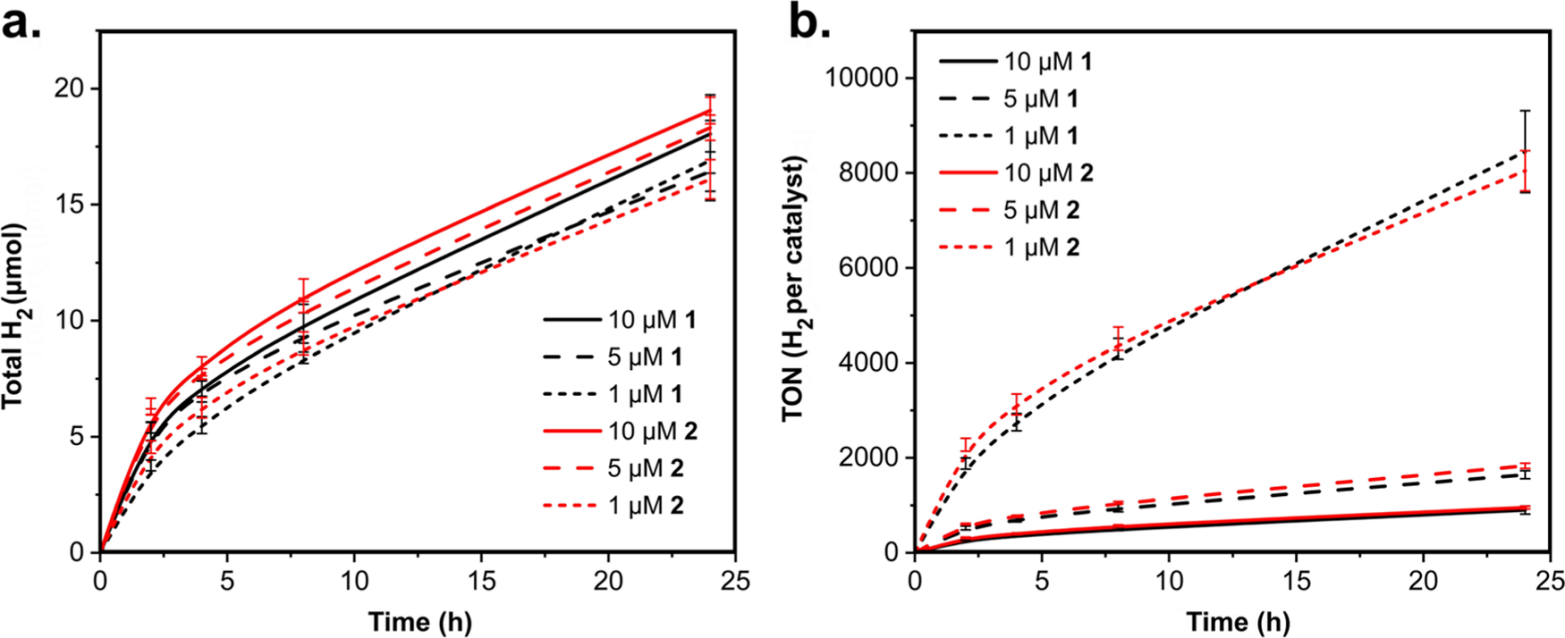
H_2_ (a) and TON of H_2_ produced per catalyst (b) measured by gas chromatography against irradiation time for different concentrations of **1** and **2** with constant visible light irradiation intensity (50 mW/cm^2^) and CIS QD concentration (QD absorbance at 405 nm of 0.35 with a 1 cm path length, corresponding to ca. 6.5 μM QDs; see text). Buffer solution: 0.5 M H_2_Asc/NaHAsc, pH = 4.5 (2 mL solution, 7 mL headspace).

To compare the hybrid-passivated CIS QDs to the CIS/ZnS core–shell QDs used in other studies and investigate whether the incorporation of the benzoic acid anchoring group to the catalyst would have any influence, photocatalytic experiments were carried out and monitored by gas chromatography measurements of produced H_2_ gas at 2, 4, 8, and 24 h of irradiation (Figures 4, S3, and S4). As the default illumination condition, light from an LED light source with an irradiance at 420–750 nm of 50 mW/cm^2^ was applied to the samples. This may be considered similar to the light intensity of the 420–750 nm region from the standard solar spectrum and was chosen to match the conditions previously applied by Nie et al. as closely as possible.^36^ The studies of Sandroni et al. and Nie et al. used 150 W Xe-lamps for irradiation, but the details on the intensity reaching the sample were not fully elaborated.^15,36^

It is known for the related systems that the rate of H_2_ production increasingly slows down over multiple hours and drops significantly after 1 day when pH = 4.5, which is attributed in the literature to either the degradation of the catalyst or the buildup of oxidized ascorbate in the form of dehydroascorbic acid (DHA), which traps electrons and hinders ET to the catalyst.^15,64^ Longer time periods were therefore not investigated for the system reported herein.

From the H_2_ measurements in Figure 4, it was observed that, under these conditions and with these concentrations of QDs (ca. 6.5 μM) and catalyst (1–10 μM), the concentration of the catalyst has only a small effect on the amount of H_2_ produced over 24 h: a decrease in concentration of either catalyst by 1 order of magnitude (from 10 to 1 μM) resulted in a decrease in hydrogen production of only about 10%. This means that the TON per catalyst decreases sharply with increasing catalyst concentration over this range.

Further control experiments determined that reducing either the irradiance or the concentration of CIS QDs by half relative to the standard conditions with 1 μM of either catalyst resulted in the amount of H_2_ produced being approximately halved. Furthermore, as expected, negligible H_2_ is produced in the absence of either light or QDs. However, in the absence of either cobalt complex, the amount of H_2_ produced is only reduced by approximately half, as the CIS QDs themselves have some intrinsic HER photocatalytic activity, which is known from previous reports.^15,16^ Nonetheless, this observed activity is noted to be higher than anticipated. Details are discussed in the Supporting Information (Figures S3 and S4 and Tables S2–S6).

Although the amount of H_2_ produced over 1 day by this system is on the same order of magnitude as that reported in the literature for systems using CIS/ZnS QDs, the behavior of the hybrid-passivated CIS system differs somewhat: for QDs with a ZnS shell, increasing the catalyst concentration resulted in notable increases in the hydrogen production, although with some loss of TON per catalyst. The TON over 24 h obtained with 1 μM of catalyst here (∼8000) is very similar to what was reported by Sandroni et al. The approximately 10-fold-to-100-fold lower QD concentration employed in our experiments may be at least partially compensated for by stronger light irradiation; the irradiation intensity is not comparable based on the published data. Nevertheless, we estimate that the quantum yield for H_2_ production is at least similar, if not higher, in the present system. Initial quantum yields of H_2_ production over the first 2 h in the experiments of Figure 4 were calculated as 2–3% (see Section S4.1 in the Supporting Information). This is similar to the monochromatic quantum yield reported by Nie et al. for CIS/ZnS and **1** (2.89%) and about half of the value for their catalyst with a dicarboxypyridine anchoring group (5.89%).^36^

It is important to note that the observed rate of H_2_ evolution is given by the rate of photon absorption multiplied by the H_2_ generation quantum yield. The latter is determined by the relative rates of several productive ET and proton transfer steps versus those of the competing charge-trapping recombination reactions. It is interesting that our hybrid-passivated CIS QDs appear to give similar quantum yields to the core–shell QDs, given that the shell is expected to retard trapping and recombination reactions.^15^

Furthermore, the effect of the presence of the anchoring group on **2** (versus **1**) seems to be practically negligible, giving only a 4% improvement over 24 h on average across the three concentrations. This was unanticipated and would at first seem to imply that the limiting factor in H_2_ production under these conditions cannot be the proximity of catalyst molecules to the hybrid-passivated CIS QDs to facilitate ET. At the same time, it was surprising to find that the yield of H_2_ was essentially independent of catalyst concentration, even at the modest concentrations employed (see above). To explore the reason for this behavior and understand the dynamics of electron transfer from QDs to the catalyst, we undertook a photophysical study of the system, and the results suggest a different explanation for the similar results for the two catalysts (*vide infra*).

### Photoluminescence Quenching by Ascorbate Buffer

We first investigated the effect of ascorbate on the PL of the CIS QDs: PL intensity quenching and time-correlated single-photon counting (TCSPC) experiments were carried out on aqueous dispersions of QDs with increasing concentrations of ascorbate buffer at pH = 4.5, without the catalyst (Figure S5). Increasing concentrations of ascorbate resulted in a linear increase in both *F*_0_/*F* and τ_0_/τ up to ca. 0.5 M, with *K*_sv_ = 6.2 ± 0.4 M^–1^. Notably, this value is 26 times the value obtained by Orlando et al. for their CIS/ZnS QDs, for which lifetimes were roughly 10 times longer.^65^ PL quenching was attributed to hole transfer processes from the QDs to ascorbate, and it is concluded that ascorbate predominantly acts as a dynamic quencher. Thus, ascorbate’s reductive quenching effect should be dependent on its local concentration and be generally slower than the oxidative quenching processes involving ET to catalyst species at the QD interface (see below). It should be noted as well that addition of ascorbate induces a moderate red shift of the PL emission peak; this is the opposite effect to that of the catalysts. This is discussed further in the Supporting Information (Section S5.1).

### Photoluminescence Quenching by Catalysts

**Figure 5:**
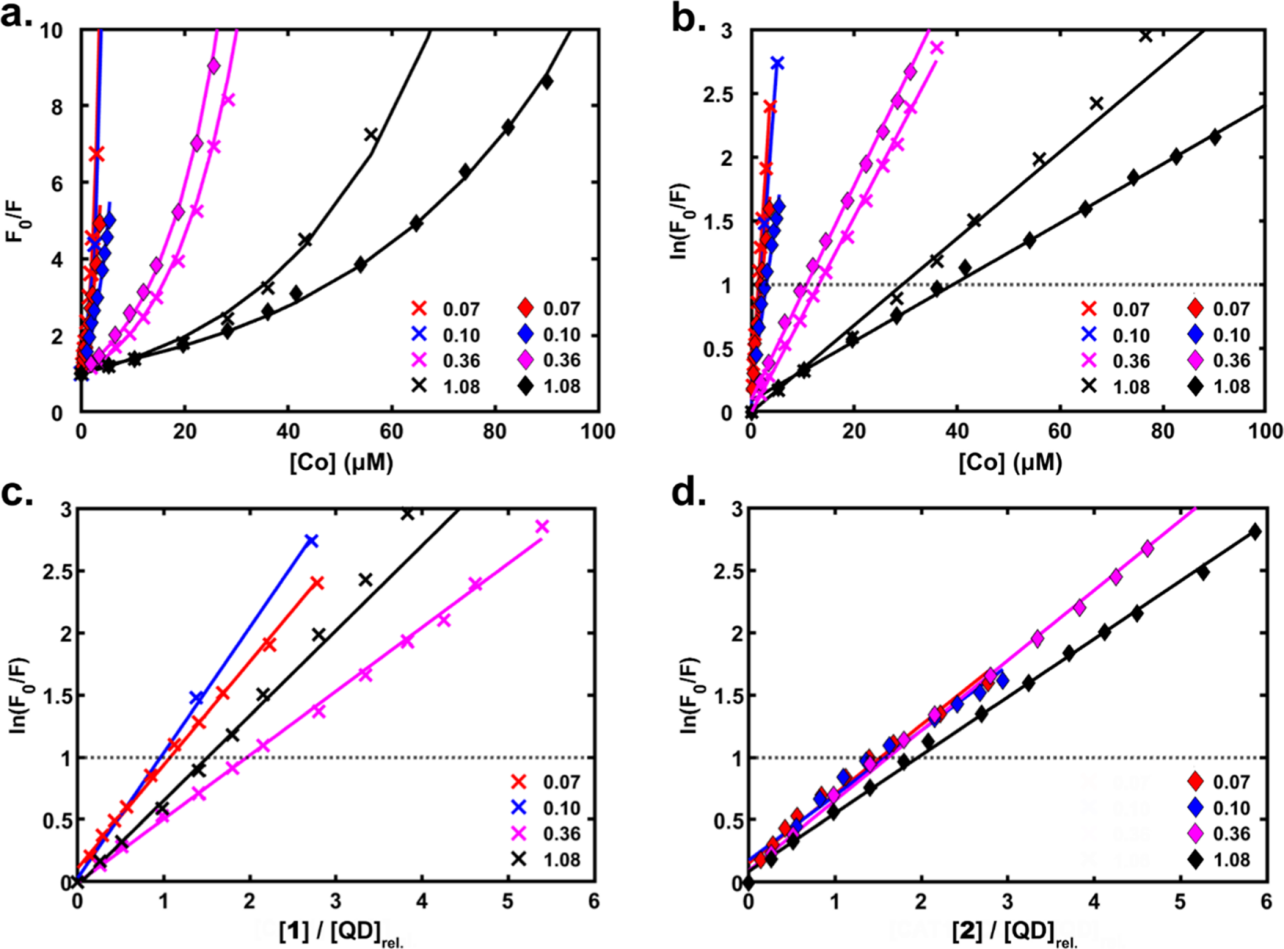
Photoluminescence quenching of the CIS QD system with **1** (×) or **2** (⧫) for different QD concentrations represented by the absorbance at 405 nm in a 1 cm cuvette, as indicated in the insets. The plots show (a) *F*_0_/*F* against the added catalyst concentration, [Co], (b) ln(*F*_0_/*F*) against [Co], (c) ln(*F*_0_/*F*) against [**1**]/[QD]_rel._, where [QD]_rel._ is the concentration relative to that of the sample with *A*_405_ = 0.10, and (d) ln(*F*_0_/*F*) against [**2**]_._/[QD]_rel._. Solid lines represent fits, and the dotted black lines in panels (b–d) indicate where ln(*F*_0_/*F*) = 1.

PL quenching experiments were carried out, combining the CIS QDs with each catalyst in different concentrations, with and without ascorbate buffer. Highly efficient quenching of the PL intensity from the QDs was observed already at very low catalyst concentrations, by 56 and 50%, respectively, for **1** and **2** at the lowest QD concentration used (Abs_405_ = 0.070, estimated to be [QD] ≈ 1.0–1.5 μM, *vide infra*). The PL intensity ratio *F*_0_/*F* (intensity without/with quencher) increased strongly with catalyst concentration (Figure 5) and was accompanied by a small blue shift of the main emission peak at 700–730 nm (see Figures S6 and S7). Because the unquenched PL lifetime is below 1 μs, the strong quenching at only ∼1 μM catalyst is inconsistent with diffusional quenching. Moreover, photoluminescence lifetime measurements by TCSPC clearly do not support a dynamic contribution; the lifetimes retrieved from triexponential fits of the PL decay profiles remain essentially unperturbed by the addition of the catalyst: τ_1_ = 27 ± 1 ns [∼66%], τ_2_ = 150 ± 4 ns [∼29%] and τ_3_ = ca. 460 ns [∼5%] (λ_ex_ = 405 nm, λ_em_ = 700 nm; see Figure S8 and Table S1). Therefore, a static quenching model with rapid (≪1 ns) quenching by catalysts preassociated with the QDs is necessary to interpret the data.

From Figure 5a, it is clear that the relationship of the PL intensity ratio (*F*_0_/*F*) against the concentration of either catalyst does not obey a linear trend, which would be characterized as a Stern–Volmer relationship and pertain to either dynamic (collisional) quenching processes or static quenching for molecular photosensitizers.^66^ Rather, the plots show a marked upward curvature: When a logarithmic scale is applied to the *y*-axis (Figure 5b–d), the data sets show a clear linear behavior, indicating that the PL intensity ratio grows exponentially with increasing concentration of either catalyst. This demonstrates that the application of the modified Stern–Volmer equation for cooperative dynamic and static quenching cannot provide an acceptable fit for the data, as this would lead to a quadratic dependence on quencher concentration.^67^ Instead, the data could be fitted with a static quenching model with a random (Poissonian) distribution of bound quenchers over the QDs. A QD with at least one quencher is rapidly (≪1 ns) quenched and gives negligible contribution to the PL intensity, as supported by our TCSPC results. Thus, the PL intensity ratio *F*_0_/*F* (eq 1) is equal to the inverse of the fraction of QDs without a bound quencher (*P*_*n*=0_), which for a Poisson distribution is *P*_*n*=0_ = *e*^–*n̅*^, where *n̅* is the average number of quenchers per QD.
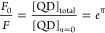
1The model is analogous to previous treatments of fluorescence quenching in micelles^68,69^ or the “quenching sphere of action” at high concentrations of quenchers in solution^66,70^ and has been applied previously to QD- and QRod-quencher systems.^43,71−74^ The previous studies with small molecule quenchers reported, in most cases, significant quenching only at much higher concentrations (mM) or with strong electrostatic attraction, showing that association in the present case is unusually strong.

The data for each [QD] was normalized using the Beer−Lambert law and the QD absorption at 405 nm, setting [QD]_rel._ = 1 for the sample with *A*_405_ = 0.10 and **1** as the quencher (Figure 5c,d). The good agreement between data for a 15-fold variation in QD concentration, when normalized to the relative QD concentration, suggests that the amount of unbound catalyst is rather small under the present conditions. If the fraction of free catalyst had been large with a low concentration of QD, it would have decreased significantly at the higher QD concentrations to give a larger slope in Figure 5c,d. We do not observe such behavior, suggesting that most of the quenchers are bound even at the lowest [QD]. The model fits the data quite well, even up to *F*_0_/*F* = 10 (*n̅*  ≈ 3) or more. This suggests that binding of the catalyst is independent of the occupancy on the QDs in this concentration range. If, instead, binding had followed a Langmuir isotherm (or other isotherms) due to catalyst interactions, as is often assumed,^43,47,74,75^ the quenchers would not have been Poisson distributed,^41^ and we would have seen deviations from eq 1 in Figure 5. We note that such isotherms are often assumed for QD/catalyst systems but are not compatible with the Poisson distribution model used to fit the data in the same studies. Compared to the latter, they would give fewer QDs without quencher and would thus overestimate *n̅* .

The data can be used to estimate the concentration of QDs (Table S7) from the known concentration of the catalyst and the value of *n̅*. In Figure 5b, the horizontal dotted black line marks the value where  equals unity. In the model, this is when the concentration of the bound catalyst is equal to the concentration of QDs (*i.e.*, *n̅* = 1). On average, **1** quenches ca. 50% more efficiently than **2;** this probably reflects more ideal binding of **1** to the QDs and unhindered ET processes. Thus, the experiments with **1** are reasoned to yield the best estimates of *n̅* and also QD concentrations. The highest quenching efficiency is observed in the experiment with **1** and a CIS QD absorbance at 405 nm of 0.10 in a 1 cm cell, which gives a QD concentration of approximately 1.9 μM (ε_405 nm_ ∼ 54,000 M^–1^ cm^–1^). Note that if the fraction of unbound catalyst is not negligible, the QD concentration would have been lower than 1.9 μM. We do, however, consider this to be the more reliable [QD] estimate and use the Beer–Lambert law to normalize the remaining data sets (Figure 5c,d). The obtained concentrations are in relatively good agreement with our calculations (see Calculation in Supporting Information Section S3.3) based on the average QD diameter and amount of cationic (In^3+^) precursor. Furthermore, quenching experiments with the similarly charged model quencher, methyl viologen (MV^2+^), follow the same model (eq 1) and yield very similar estimates of [QD] (see Figure S9), supporting the validity of these assumptions.

It is noted that the presence of ascorbate increases the values of *n̅* obtained from the fits (Figure S10); the reasons for this are not clear. We do not believe that this effect is because of the presence of ascorbate increasing the degree of catalyst binding, as the data in Figure 5 suggests that the binding is already nearly complete. We have also excluded pH-induced alterations in the catalysts, such as changes in their oxidation states (*vide infra*) or reduction potentials, since we observe comparable enhancements in the quenching efficiencies with either methyl viologen (MV^2+^) or ferrocyanide (Fe(CN)_6_^4–^), which would not change oxidation state due to an acidic environment (either acetate or ascorbate buffer at pH 4.5).^76^ We tentatively suggest that the increased ionic strength induces some agglomeration/aggregation that enables individual catalyst molecules to effectively act as static quenchers for groups of more than one QD that are in close contact. In any case, it is clear that ascorbate by itself quenches the QD PL much more slowly than the catalysts, so that the primary interaction of the QDs is with the catalyst.

Overall, it is intriguing that both catalysts bind so strongly to the QDs. Ultrafast quenching by metal complex catalysts has been inferred before,^3,7,8^ and electron transfer has been directly demonstrated by TAS, even on a sub-ps time scale,^39,40^ but only in the presence of excess catalyst. Here, we show that catalysts are predominantly bound to the QDs even at concentrations as low as 1 μM of each species, meaning that the fraction of unbound catalysts is surprisingly small and that single catalysts per QD are sufficient to completely quench PL. The reduction potentials of the catalysts suggest that ET from the CIS QDs to the catalyst is thermodynamically favorable, and the lack of a spectral overlap between the QD PL and catalyst absorption spectra rules out significant contributions from Förster energy transfer (FRET) processes. PL quenching experiments, therefore, suggest efficient oxidative quenching by surface-adsorbed **1** and **2** with high association constants, inferring electron transfer rates faster than the time resolution of the TCSPC experiments (<100 ps). The latter is confirmed by the TA results below.

### Direct Catalyst Reduction by Ascorbate Buffer

It has been noted in previous studies^24,29,33,36^ that ascorbate also acts as an effective and quantitative reductant to irreversibly convert **1** from Co^III^ to Co^II^. To verify this for **1** and **2**, titration experiments were carried out under O_2_-free conditions in a glovebox, monitored by absorbance spectroscopy. By titrating ascorbate buffer into a solution of either catalyst (or *vice versa*), complete conversion of Co^III^ to Co^II^ by only 0.5 equiv of ascorbate (total of protonated and deprotonated forms) is indeed observed, indicating ascorbate’s behavior as an overall two-electron donor (see Figures S11–S13), noting that two ascorbyl radicals disproportionate to generate one DHA molecule and regenerate one ascorbate ion. Under atmospheric conditions, however, O_2_ competes with ascorbate to continuously reoxidize **1** and its derivatives to Co^III^, so this is understood to comprise the majority of the population of cobalt species in the experiments carried out in the presence of oxygen.

In the full QD–catalyst system, on the other hand, the results of titration experiments are more intricate as the model redox behavior of **1** with ascorbate is substantially complicated by the presence of CIS QDs (see Figures S14 and S15). In contrast to the titration experiments with free catalyst and ascorbate, without QDs (discussed in the previous paragraph), it appears that the entire population of Co^III^ cannot be reduced to Co^II^, even when ascorbate is in excess (∼45 equiv HAsc^–^ per catalyst), and the concentration of CIS QDs is relatively low, meaning that some Co^III^ active sites persist (see Supporting Information, Section S5.7). Furthermore, PL quenching controls in 0.1 M ascorbate buffer sample solutions prepared under an O_2_-free glovebox result in PL intensity ratios similar to the samples prepared under atmospheric conditions (see Figure S16), where O_2_ will be regenerating the Co^III^ state. The negligible difference in the PL quenching efficiency in the presence and absence of oxygen indicates either that (i) both the Co^II^ and Co^III^ forms of both catalysts quench PL oxidatively with roughly equal efficiencies (i.e., statically) or (ii) the Co^III^ initial state is prevalent for the majority of the surface-adsorbed catalysts prior to photoexcitation, even in the absence of oxygen. Ultrafast fs-TA was therefore employed to follow the formation and give direct evidence of charge separation products in both the presence and absence of oxygen.

### Charge Carrier Dynamics in CIS Quantum Dots

**Figure 6:**
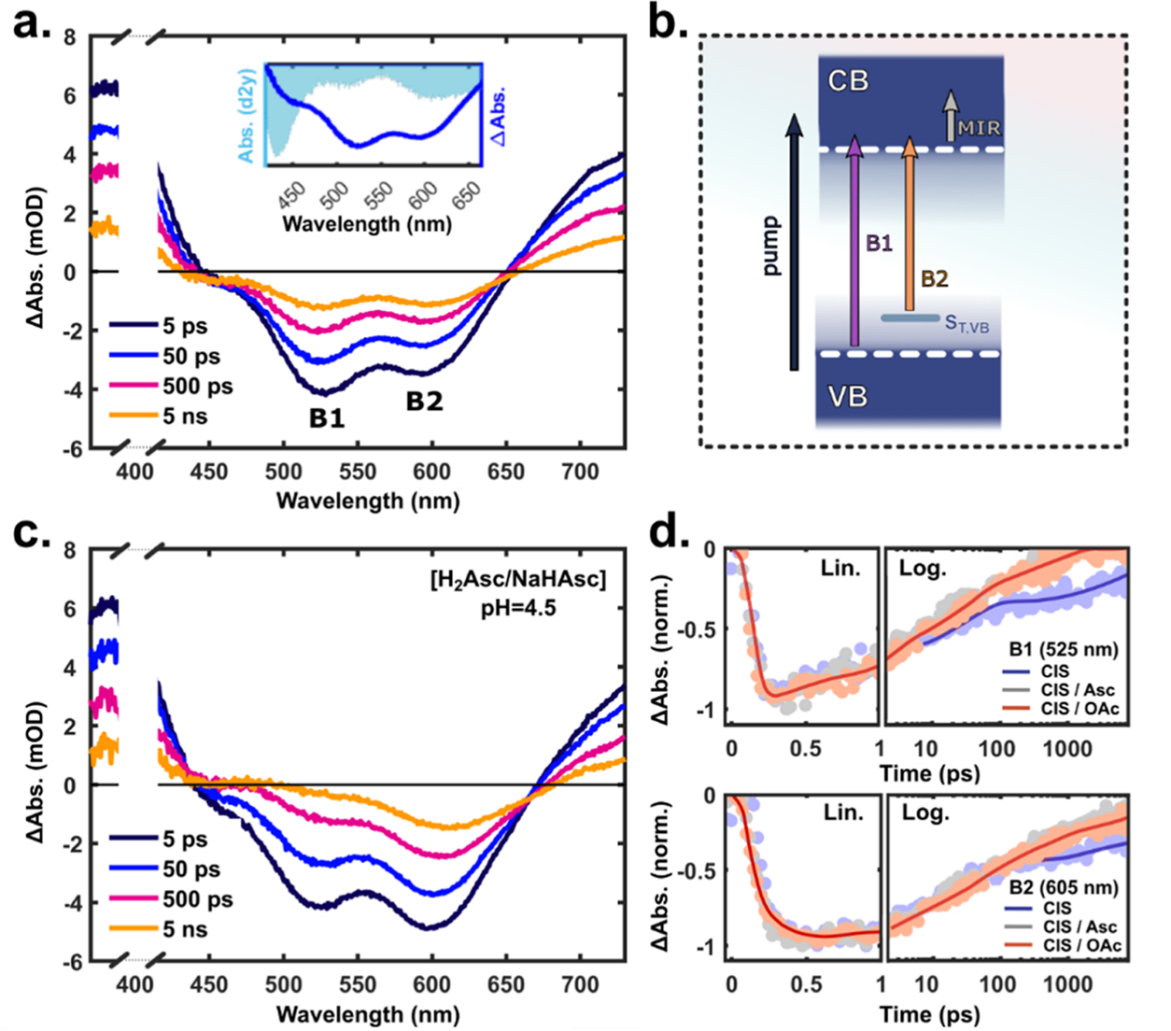
Transient absorption (TA) measurements with a 400 nm excitation pulse. (a) TA difference spectra of CIS QDs in H_2_O at indicated time delays. The inset shows the second derivative of the QD ground-state absorption spectrum (light blue, shaded), together with the TA spectrum at 50 ps pump–probe time delay (dark blue). (b) Schematic of the pump-induced optical transitions monitored in the UV–vis (B1/B2 bleach bands) and MIR (PIA). (c) TA spectra of CIS QDs in H_2_Asc/NaHAsc buffer (pH = 4.5, 0.1 M). (d) Kinetic traces at 525 and 605 nm of CIS QDs in H_2_O (blue) and in buffer at pH = 4.5 (H_2_Asc/NaHAsc: gray, NaOAc/HOAc: red). The pump scattering has been removed from the spectra in panels (a) and (b) for clarity (white cutout).

The fs-TA spectra of the CIS QDs in water after 400 nm excitation (Figure 6a) show negative bands at ∼430 nm (B0), ∼520 nm (B1), and 610 nm (B2) as well as broad positive signals (<400 nm, >525 nm) extending into the NIR and MIR regions (Figure S17). Similar features are observed for pump wavelengths between ∼340 and 520 nm, with negative bands corresponding to the second derivative of the ground-state absorption spectrum (inset of Figure 6a). This indicates that the fate of the carriers, after initial relaxation, remains indifferent to excess energies upon excitation above and similar to the optical bandgap. Excitation across the absorption tail band (∼550–650 nm), on the other hand, results in TA spectra dominated by the B2 bleach band (Figure S17c). We attribute the B1 and B2 bands to the bleaching of two independent optical transitions dominated by state-filling effects involving VB–CB (B1) interband and S_T,VB_–CB (B2) sub-bandgap optical transitions, as illustrated in Figure 6b. This assignment is consistent with our previous study on similar QDs (albeit with higher Cu deficiency).^39^

The bleaching of optical transitions results from the reduced oscillator strength experienced by the probe pulse due to pump-generated carriers occupying the involved states, effectively lowering the probability of probe absorption in those spectral regions. The lowest energy band-edge exciton bleach (B1) dynamics is usually considered to be dominated by the CB-edge (1S_e_) population due to the higher density and degeneracy of the VB as well as fast hole trapping events.^77^ The latter is likely responsible for the few hundred femtosecond increase of the B2 bleach band (Figure 6d), i.e., the bleaching of Cu^+^–CB transitions due to hole localization events converting optically active Cu^+^ to inactive Cu^2+^. The broad and featureless positive signals in the visible and NIR region presumably have contributions from electron and hole intraband transitions as well as trap states, but their origin will not be discussed further herein. In the MIR, we associate the probe pulse absorption induced by the pump pulse to intra-CB transitions (Figure 6b, gray arrow) with possible contributions from shallow trap states.

The overall TA decay dynamics are multiexponential, reflecting several processes and decay channels for the different carriers. The recovery of the B1 bleach band for neat CIS can be fitted with a sum of three exponentials (τ_1_: 2–3 ps [30–40%], τ_2_: 30–50 ps [25–35%], and τ_3_: >8 ns [30–40%] over five different QD batches), whereas the B2 band is dominated by the two longer time components τ_2_ (50–60%) and τ_3_ (40–50%).

In the presence of the ascorbate buffer (H_2_Asc/NaHAsc, pH = 4.5), the QD signals in all probe regions (UV–vis/NIR/MIR) are subject to both static (relative signal intensities at *t*_0_) and dynamic (*t* > *t*_0_) changes compared to neutral pH, see Figure 6a vs c. The same CIS QD optical response is seen in acetate buffer (NaOAc/HOAc, pH = 4.5), which excludes hole transfer on these time scales and rather indicates a sensitivity to pH, e.g., changing the physiochemical environment on the QD surface. In particular, the B1 bleach band recovery and MIR decay are accelerated in the presence of the buffers (see Tables 1 and S8), which points toward effects on the CB population (as opposed to hole dynamics). Note that we do not observe any dynamic red shift of the bleach band that otherwise would suggest that the accelerated loss of these signals is related to inter-QD transfer caused by partial aggregation; however, QD surface changes could, e.g., result in a higher abundance of trap states that capture photogenerated carriers (electrons).

**Table 1:** Fitting Parameters and Electron Transfer Rate Constants Obtained from Multiexponential Fits^a^

UV–vis^b^ (525 nm)	τ_1_ (*A*_1_) [ps]	τ_2_ (*A*_2_) [ps]	τ_3_ (*A*_3_) [ps]	τ_4_ (*A*_4_) [ps]	<τ>^d^ [ps]	<*k*_ET_>^e^ [ps^-1^]	<τ_ET_>^e^ [ps]
CIS	1.8 (35%)	28 (33%)	610 (23%)	4700 (8.1%)	33		
CIS/1	1.3 (47%)	19 (35%)	270 (15%)	4400 (4.1%)	10	0.070	14
CIS/2	0.90 (39%)	11 (35%)	170 (20%)	4100 (6.6%)	11	0.065	15

MIR^c^ (3820 nm)	*τ*_1_ (*A*_1_) [ps]	*τ*_2_ (*A*_2_) [ps]	*τ*_3_ (*A*_3_) [ps]	*τ*_4_ (*A*_4_) [ps]	<*τ*>^d^ [ps]	<*k*_ET_>^e^ [ps^–1^]	<*τ*_ET_>^e^ [ps]
CIS	2.0 (29%)	20 (37%)	240 (22%)	4100 (12%)	33		
CIS/1	0.83 (39%)	7.9 (33%)	110 (20%)	4200 (8.6%)	9.7	0.073	14
CIS/2	1.0 (38%)	10 (34%)	190 (18%)	4900 (9.9%)	13	0.046	22

a Kinetics traces extracted from the data sets in ascorbate buffer (pH = 4.5) shown in Figure 7 with an average catalyst number *n̅* = [Co]/[QD]_rel_.

b 0.7 in the UV–vis ([Co] = 50 μM).

c 0.6 in the MIR ([Co] = 70 μM).

d The amplitude-weighted average lifetimes <τ> were calculated from eq 3; see main text.

e The average electron transfer rate constants and lifetimes were calculated from eq 5.

### Photoinduced Electron Transfer: Co^III^ to Co^II^

The addition of approximately one equivalent of either **1** or **2** to the CIS QDs results in significant changes to the optical response in the MIR and UV/vis probe regions, as shown in Figure 7 (see also Figures S19 and S20). Note that these experiments were performed in ascorbate buffer (H_2_Asc/NaHAsc, pH = 4.5) with oxygen present; see Figures S21, S24, and S25 for controls without buffer and oxygen, respectively. First, the broad positive QD feature in the MIR shows a significantly accelerated decay upon addition of either catalyst, consistent with the depletion of pump-generated CB electrons upon ET from the photoexcited QDs to the catalysts (see Figure 7b).

**Figure 7:**
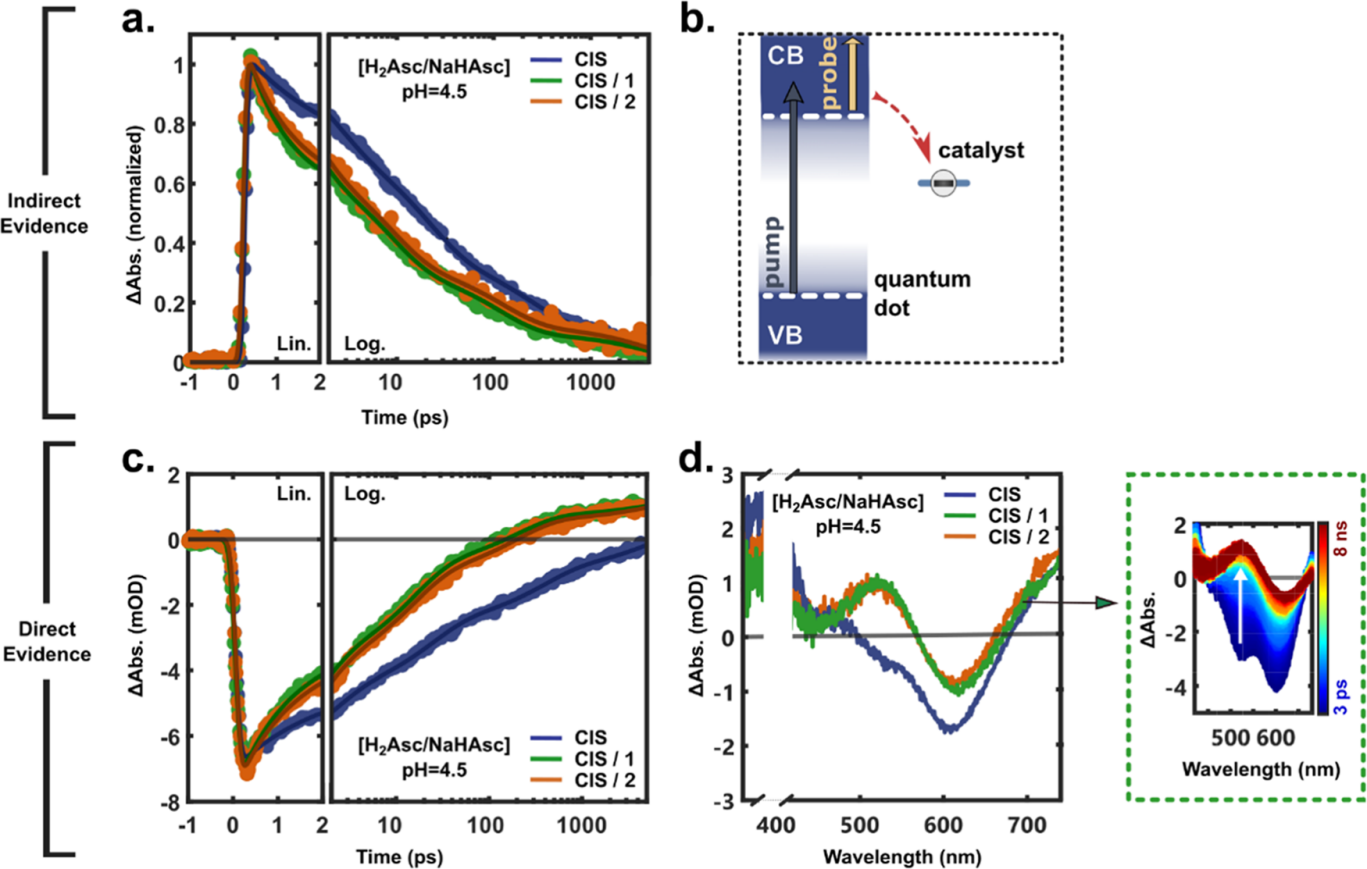
Transient absorption spectra (λ_pump_: 400 nm) of CIS quantum dots, QDs, without (blue) or mixed with **1** (green) or **2** (orange) in 0.1 M H_2_Asc/NaHAsc buffer (pH = 4.5). (a) Normalized MIR kinetics averaged between 3600 and 4000 nm (2780 and 2500 cm^–1^) with an average catalyst number *n̅* = [Co]/[QD]_rel._ of ca. 0.6 ([Co] = 70 μM). (b) Schematic representation of pump and probe in the MIR together with the transfer of electrons from the CB of photoexcited QDs to the catalysts. (c) Kinetic traces extracted at the PIA of the reduced catalysts (Co^II^) in the UV–vis probe region (∼520 nm) with a catalyst/QD ratio of *n̅* ≈ 0.7 ([Co] = 50 μM). The corresponding difference spectra at *t* = 2 ns are presented in panel (d), with a close-up of the CIS/**1** spectral evolution from 3 ps (dark blue) to 8 ns (dark red).

Second, in the UV–vis probe region, the presence of either catalyst leads to an accelerated recovery of the B1 bleach band while the lower energy B2 bleach band remains relatively unperturbed on the lower energy side. This is consistent with our assignment of a significant contribution from (localized) holes to the B2 dynamics, which renders it rather insensitive to the survival probability of the 1S electron. Superimposed on the bleach bands, we observe the formation of a long-lived PIA, as evident in Figure 7c,d. The PIA is in relatively good agreement with the expected contribution from a Co^II^ ET product based on Co^II^ and Co^III^ reference spectra (see Figures S11 and S14). The derivative-like shape of the TA signal on longer time scales is, however, reminiscent of a Stark-effect-induced signal; such signals may form upon charge separation across the interfacial region due to the emerging electric field. To assess this possibility, we performed control experiments with MV^2+^ as the electron acceptor (Figure S22); knowing that MV^2+^ quenches the PL statically (*vide supra*), we may expect a similar derivative-like signal from the QD-MV^2+^ charge-separated state. The transient response from surface-adsorbed MV^2+^, however, shows no indication of Stark-shifted optical transitions. The PIA from its radical cation is clearly distinguishable and superimposed on the QD signals within 1 ps, consistent with the static PL quenching (50% PL quenched at sub-μM [MV^2+^], see Figure S9). Furthermore, the Δ*T*A spectra (Δ*A*_QD/CAT_ – Δ*A*_QD_) of the QD–catalyst system with ascorbate show nearly identical spectral signatures to the unbuffered samples (Figure S21), suggesting that the ET product is Co^II^ in both cases, i.e., that the initial state prior to QD excitation is dominated by Co^III^ when exposed to oxygen. Hence, we attribute the PIA in the QD–catalyst TA spectra to Co^II^ formation.

Due to the complexity of the system in buffer, we fit the data sets with the number of exponential terms required to adequately describe the data (minimize residuals). Representative kinetics and fit parameters corresponding to the data set shown in Figure 7 are presented in Table 1. The fits to the UV–vis data with the catalyst included an offset to account for the remaining signal of the Co^II^ state at the end of the experiment. It is clear that the catalysts reduce all lifetime components τ_1_–τ_3_ while leaving their relative amplitudes relatively constant. This means that ET to the catalyst occurs on a range of time scales. Moreover, the data includes contributions from QDs with different number of catalysts, including those with no catalyst. Attempts were made to analyze the kinetic traces with a multiphasic version of the Infelta–Tachiya equation^68,69^ developed for a Poisson distribution of fluorescence quenchers in micelles
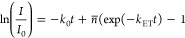
2A sum of three such expressions with different values of *k*_0_ and *k*_ET_ gave, perhaps not surprisingly, unreliable results because of too many fit parameters and complications from overlapping signals from the Co^II^ product.

Instead, we use the multiexponential fits that, although they do not exactly describe the behavior of eq 2, give a reasonable description of the behavior of the heterogeneous kinetics of the present system. Thus, we compare the average lifetimes <τ> obtained from the amplitude-weighted average log_10_(<τ>) with and without addition of catalysts:^78^
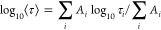
3In eq 3, *A_i_* and τ*_i_* are the relative amplitude and lifetime of the *i*th component from the multiexponential fit (the infinite component in the presence of the catalyst is ignored in the average). Similarly, the average rate constant <*k*> is given by
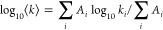
4This definition of average <τ> and <*k*> has the satisfactory property that <τ> = 1/<*k*>. This is not the case for the common linear or quadratic amplitude-weighted average lifetimes and rate constants, which gives undue weight to either the longer lifetime or larger rate constant components. Note that a lower number of exponential terms than four yields similar results for <τ> but results in oscillating fit residuals for some of the data sets in ascorbate buffer (see Table S8 for more fits).

We define the average ET rate constant as
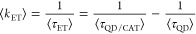
5The average ET rate constants obtained from the multiexponential fits of the B1 bleach band yield <*k*_ET_> ∼(14 ps)^−1^ and ∼(15 ps)^−1^ for **1** and **2**, respectively. These values agree fairly well with the values obtained in the MIR region: <*k*_ET_> ∼(14 ps)^−1^ and ∼(22 ps)^−1^ for **1** and **2** at 3820 nm, respectively, albeit with a slightly lower average catalyst number per QD (*n̅* = 0.6) compared to the UV–vis data (*n̅* = 0.7). At *n̅* = 0.7, ca. 50% of the QDs have no catalyst, while ca. 35% have one. Although the dependence of <*k*_ET_> on *n̅* is not simply linear, no major additional uncertainty is introduced by assuming that <*k*_ET_> would be approximately twice as large in the ideal case of exactly one quencher for every QD. This gives an estimated electron transfer rate constant on the order of ≈(10 ps)^−1^ for *n* = 1.

An alternative fit model was also used, in which the fit parameters for the QDs without catalyst were fixed and scaled according to the fraction of QDs with *n* = 0, exp(−*n̅*). Then, a sum of two or three exponents was added to account for the fraction of QDs with *n* ≥ 1. This should thus slightly overestimate *k*_ET_ at *n* = 1 and result in somewhat smaller values than above: <τ_ET_> ≤ 7 ps (see Supporting Information, Table S9). We note that the average catalyst numbers obtained from allowing the scaling factor exp(−*n̅*) to vary freely agree well with the *n̅* estimated from the modeling of the PL quenching data.

The QDs without catalysts result in a fraction of the QD MIR signal that remains for the duration of the experiment in the presence of the catalysts, with dynamics similar to that of the catalyst-free measurements. If the number of catalysts per QD is Poisson distributed, a catalyst/QD ratio of *n̅* = 0.6 is expected to leave 55% of unperturbed QD signal (*n* = 0). This is in good agreement with the PIA/PI*A*_0_(*t*) intensity ratio of ca. 60% after a few tens of ps (when ET is essentially complete) in the presence of **1**, albeit slightly higher for **2** (∼70–80%), see Figure S19. The similar trend in the PL quenching data indicates that **2** has a lower binding affinity compared to **1**, and thus a larger subset of QDs without any bound catalyst at a given *n̅* = [Co]/[QD]_rel._. This, together with the essentially indifferent ET rates for **1** and **2**, points toward similar binding sites and ET geometry for both catalysts.

### Competitive Co^III^ and Co^II^ Reduction

In the absence of oxygen, on the other hand, we may expect some buildup of Co^II^ since HAsc^–^ is known to reduce **1** in the dark (Co^II^ to Co^III^ reoxidation by oxygen can prevent such a buildup).^29,33,36^ By titrating ascorbate into an oxygen-free **1** solution, we indeed observe complete Co^III^ to Co^II^ conversion by only 0.5 equiv of ascorbate (Figure S11). As discussed before, however, there are indications that the Co^III^ to Co^II^ reduction by HAsc^–^ is not quantitative in the presence of CIS QDs (see Figures S14 and S15). TA measurements with QD/catalyst ratios of ∼1:1 (H_2_Asc/NaHAsc, pH = 4.5) in oxygen-free solutions did not result in any new features reminiscent of Co^I^ expected from catalysts initially in the Co^II^ state, if the ET rates from Co^II^ to Co^I^ are sufficiently fast to be observed in the experiment (<8 ns). If the reduction of surface-adsorbed catalyst by HAsc^–^ is indeed incomplete, a faster Co^III^ to Co^II^ reduction by the QDs may outcompete Co^II^ to Co^I^ if the QD has more than one catalyst bound (i.e., *n >* 1). We therefore compared the TA response across varying concentrations of **1** (10, 30, 60, 180 μM), with an estimated range of *n̅* = [**1**]/[QD]_rel._ = 0.1–2 in the presence and absence of oxygen. When exposed to atmospheric oxygen, the positive transient from Co^II^ increases in magnitude with increasing [**1**] and appears to saturate at higher [**1**]; see Figure S23. The latter is consistent with the reduction of mainly one catalyst/QD at these pump fluences (<absorbed photons>/QD < 1), i.e., the transiently formed [Co^II^] is proportional to the QD population with one *or* more (*n* ≥ 1) catalyst bound, 1 – exp(−*n̅*). The Co^I^ species, however, remains elusive. In the oxygen-free samples, we do not observe any new signal associated with Co^I^ formation at any QD/catalyst ratio, despite its expected strong signal in the blue (and red) region of the TA spectra based on its characteristic peaks in acetonitrile.^24^ For the three lower catalyst/QD ratios (*n̅* ≤ 1), however, we observe less PIA from Co^II^ compared to the aerated samples (Figures S24 and S25). Hence, it appears that a subpopulation of the catalyst is indeed in the Co^II^ state prior to photoexcitation under O_2_-free conditions and may participate in ET reactions on time scales beyond our measurable time window (>8 ns). In contrast, the TA response for the highest [**1**]/[QD] (*n̅* ≈ 2) is indifferent to the presence or absence of oxygen; it thus seems that at higher occupation numbers, most QDs accommodate at least one Co^III^ and any Co^II^ cannot compete for photogenerated electrons.

## General Discussion

Altogether, this study showcases the high catalytic activity of the system in question for photocatalytic H_2_ evolution, comparable to that reported for CIS/ZnS core–shell QDs with **1**.^15,36^ The novel derivative of **1** also works similarly well to produce H_2_ under these conditions, but its anchoring group is not found to provide any significant improvement in these experiments. Cyclic voltammetry in MeCN shows a positive potential shift in both the Co^III/II^ and the Co^II/I^ redox couples by ca. 125 mV (Figure S26), caused by the –CO_2_H substituent, which notably also does not lead to any obvious effect in the photocatalytic experiments.

Indeed, despite lacking the intended anchoring group, **1** generally proves to be the marginally more effective quencher, implying that a specific anchoring group is not necessary in this system for the molecular catalyst to interact strongly with the QDs. The lack of apparent sensitivity to the binding group suggests that the native ligand layer is not favorably exchanged by **2**. Thiolates are known to bind strongly to “soft” metal cations^79^ such as Cu(I) and can be presumed to outcompete the carboxylic acid anchor for the same sites—this is also a possibility in the case of the 2,6-dicarboxypyridin-4-yl anchor previously employed.^36^ Nevertheless, for photocatalysis, the modification at the *para-*pyridine position of the macrocycle did not adversely affect the catalyst’s comparative performance, indicating that this site can be functionalized without impeding the catalytic mechanism, which validates modification at this position for improvement of this catalyst following rational design principles.^34,35,80^

Similar TONs were recorded to those reported for CIS/ZnS, in spite of using hybrid passivation, and it is estimated that the quantum yields are also similar: initial quantum yields of H_2_ production over the first 2 h in these experiments were calculated as 2–3% (see SI Section 4.1). This is interesting, as the shell has been described to reduce charge recombination and unproductive trapping. Quantum yields were not determined in Sandroni et al.,^15^ but Nie et al.^36^ reported a quantum yield of 5.35%, with CIS/ZnS and dicarboxypyridine-anchored **1**. In this respect, it is interesting to note that Wu and co-workers reported a quantum yield of 20% for CIS QDs with Glu ligands and nickel(II) ions, assumed to form a cocatalyst *in situ*, although the very high light intensity (3 W LED light at 470 nm) may complicate direct comparison with other systems.^16^

We further note that direct comparison with literature reports is complicated in general, not only because of quite different and sometimes ill-defined irradiation intensities at the samples but also large uncertainties in [QD]. The previous papers gave a QD concentration of ca. 110 μM in most experiments, but the reported absorbance at 420 nm in a 1 cm cuvette (0.366) of Sandroni et al.^15^ is at least 1 order of magnitude too small to agree with the concentration and extinction coefficient given. Nie et al.^36^ reported up to 90% PL quenching of 110 μM QDs by only 10 μM catalyst, which is not possible with the short QD PL lifetimes reported. We thus believe that their QD concentrations could be at least 1 order of magnitude smaller than the values given. Critically, this must necessarily affect comparisons of H_2_ production.

For the system investigated here, it is particularly noteworthy that practically all QDs bind at least one catalyst, even at only a few equivalents of catalyst to QD, and in the absence of any anchoring group. This may also explain why this specific catalyst is reported in the literature to perform surprisingly well with various QDs under conditions at which many other molecular catalysts do not work so well (i.e., without a large excess of photosensitizer and with relatively high catalyst concentration).^15^ The origin of this favorable binding interaction is not clear; we note that **1** is soluble in water and its electrocatalytic reactions have been investigated in aqueous solution, so association to the QDs should not be simply due to a hydrophobic effect. Electrostatic attraction of the cationic catalysts should not be the main reason, as even the tetraanionic quencher [Fe(CN)_6_]^4–^ gives significant quenching at only 10 μM with ∼1 μM QDs in the presence of ascorbate.^76^ Moreover, an increase in quenching efficiencies was observed with addition of either ascorbate or acetate buffer (pH = 4.5) for both positively and negatively charged model quenchers (MV^2+^ and [Fe(CN)_6_]^4–^). Per purely electrostatic considerations, a higher local [H^+^] (shifting the ζ-potential positive) would be expected to result in opposite trends for cations and anions. The strong QD–catalyst association is nevertheless desirable, and further understanding would be helpful for system design.

The process studied by fs-TA was the photoinduced electron transfer leading to the reduction of Co^III^ to Co^II^, but to complete the catalytic cycle, at least one further reduction step and two protonation steps are required. Investigation of further reduction of the Co^II^ catalyst, by PL quenching and fs-TA with ascorbate under an argon atmosphere, suggested that a significant fraction of **1** remains in the Co^III^ initial state, in contrast to previous reports. We observe no formation of Co^I^, although it would have a strong signal in the blue range of our TA spectra. Therefore, we conclude that reduction of the Co^II^ state of the catalysts is slower than the time scale of the experiment, i.e., >8 ns. In contrast, similar experiments with CIS/ZnS but with only observation of QD signals (PL and fs-TA) led Wang and co-workers to propose that the rates of electron transfer from QDs to Co^II^ and Co^III^ were very similar (τ ≈ 1 ns).^36^ While some studies show much slower electron transfer to Co^II^ than to Co^III^,^81,82^ other studies show more similar rate constants, and this is strongly dependent on the Co ligand set and the potential of the sensitizer.^65,83^ We note, however, that if both the samples with and without ascorbate also contained mainly the Co^III^ state of the catalyst in the study of Wang and co-workers, as we observed here, this could explain the similarity of their results, and reduction of the Co^II^ state would probably have been much slower than 1 ns.

It is important to understand whether the catalyst also remains bound throughout the whole catalytic cycle and, in particular, the Co^II^ state. To this end, we note, first, that the rate of photocatalytic H_2_ production is essentially the same with either 10 or 1 μM catalyst. If a large fraction of Co^II^ would be unbound, we would expect to have seen limitations in electron transfer efficiency from diffusion. Second, the TOFs per catalyst of our system are close to those reported from optimized conditions with different photosensitizers and electrochemistry: the calculated TOFs of either catalyst were 0.26–0.32 s^–1^ over 2 h and 0.093–0.098 s^–1^ over 24 h (Table S3). Noting the greater longevity of the QD-based systems,^15^ this compares reasonably with the reported photochemical initial TOFs of 0.09–0.10 s^–1^ with excess [Ru(bpy)_3_]^2+^ in similar ascorbate buffers.^25,30^ It also agrees with the theoretical TO*F*_max_ of 0.168 s^–1^ that would be set by the rate-determining second protonation step from electrochemical benchmarking (5300 M^–1^ s^–1^ × [H^+^], when pH = 4.5),^23^ despite photon absorption being the key limiting factor in the present case. Assuming that **1′**s characteristic ECEC heterolytic mechanism remains in play, this indicates that the critical Co^I^ intermediate is still generated efficiently. This suggests that Co^II^ is reduced efficiently, without major charge recombination losses that would be expected from diffusional limitations at 1 μM reactant concentrations. Third, we note that the PL quenching efficiency by the catalysts in 0.1 M ascorbate buffer is largely indifferent to the presence or absence of oxygen, although fs-TA experiments hint that a subset of the catalyst is in the Co^II^ state prior to photoexcitation in ascorbate buffer and in the absence of oxygen. At low [Co]/[QD] ratios, many QDs have only one catalyst, and Co^II^ may thus be reduced by a QD fast enough to quench that QD’s PL with almost 100% efficiency, which cannot be the result of diffusional quenching.

We thus infer that the Co^II^ catalysts are also bound to the QDs. At higher [**1**]/[QD] in fs-TA measurements, most QDs have at least one bound Co^III^ catalyst and a Co^II^ catalyst on the same QD cannot compete for photogenerated electrons due to the greater favorability of ET to the more oxidized Co^III^ species. This makes the direct observation of Co^I^ formation difficult at any catalyst/QD ratio that can be practically investigated by fs-TA. We therefore propose that the catalyst remains largely bound to the QDs throughout the photocatalytic cycle, although spectroscopic evidence for Co^I^ formation is lacking. The strong binding and rapid photoreduction of the catalyst in either oxidation state explain in part why the photochemical H_2_ production rate hardly increases with increasing catalyst concentration above the concentration of QDs in our experiments. Already at low catalyst concentrations, most QDs have one catalyst bound, and this is sufficient to harvest most photogenerated charge carriers. Higher catalyst concentrations may instead lead to competition for redox equivalents and increase recombination losses or comproportionation (Co^I^ + Co^III^ → 2Co^II^).^27^

Overall, this gives a very different picture of the catalytic cycle in our CIS QD–cobalt catalyst system, where catalyst diffusion is not a key limiting process, versus the common general assumption for dissolved photosensitizer–molecular catalyst systems, where catalyst diffusion is presupposed to be rate-limiting (see, e.g., ref (38)). In the system we present here, the latter is clearly not correct, and while it is not necessarily true for all QD/catalyst combinations and conditions, other systems in the literature also exhibit static quenching behavior, in most cases thanks to a designed anchoring group or electrostatic attraction.^3,5−7,33,36,39,40,42,46,50,59^ Moreover, mass transport of water substrate (55 M) and ascorbate (0.5 M) is also not rate-limiting in the present study. Ultimately, the rate-limiting factors here, as well as in systems where the catalyst needs to diffuse, are, first, the rate of photon absorption, which is at best on the order of 10 s^–1^ per QD under ca. 1 sun irradiation, and second, the quantum yield, which is typically ∼10% or lower (see Section 4.1. in the Supporting Information). The low quantum yield is a consequence of various charge recombination and trapping processes, as well as side reactions. Thus, increasing the QD–catalyst electron transfer rate may not lead to increased efficiency, if the rate is already large enough to outcompete other exciton decay pathways. Similarly, an intrinsically high catalyst turnover frequency (TOF) may not impart any advantage if the catalyst is not limiting the overall photocycle. Instead, catalyst stability while waiting for the second electron and/or proton should be a critically important property. In this case, enhancing catalyst stability rather than intrinsic TOF or ET rates should be the first priority, i.e., incorporating catalysts which reliably and consistently survive catalytic cycling through their reactive intermediate states for the necessary time periods. In this context, **1** is a logical choice versus other cobalt tetraazamacrocycles, and this may at least partially explain its excellent performance with CIS and CIS/ZnS QDs.

While the present experiments suggested that the catalysts were predominantly bound, and it was not possible to register an important difference in either ET rate or photocatalysis with or without the intended anchoring group, we note that stronger, specific binding strategies may be more important in heterogenized systems. For example, with QDs and catalysts immobilized on electrodes for photoelectrochemical water splitting, strong binding may prevent gradual leaching of the catalysts from the electrode surface. For CIS and other metal chalcogenide QDs, better binding groups than carboxylate would be preferred, e.g., “softer” ligands.^79^

It is encouraging that good photocatalytic results are obtained with the hybrid-passivated CIS QDs, as their small size is favorable for sensitization of mesoporous photocathode materials, such as NiO, while also avoiding damaging the NiO layer.^59^ This may open the way to more photoelectrochemical designs with this QD–catalyst system.

## Conclusions

To summarize, the effectiveness of CIS QDs with hybrid passivation under photocatalytic conditions is demonstrated for the time scales studied (24 h), giving TONs of up to and above ∼8000 at very low catalyst concentrations. A novel, catalytically active derivative of the hydrogen-evolving catalyst [Co(N_4_H)Cl_2_]^+^ is also reported. We propose that the high performances reported for many combinations of quantum dots and molecular catalysts for photocatalysis to produce solar fuels can, in some cases, be attributed to the unanticipated formation of tightly bound complexes in solution, even in the absence of specifically designed anchoring groups. Femtosecond TA spectroscopy confirms the reduction of both catalysts by direct observation of the reduced states on ultrafast time scales ≈ (10 ps)^−1^ upon photoexcitation of the CIS QDs. The fact that this reduction occurs faster than the diffusion limit confirms the catalysts’ adsorption onto the QDs and the consequent static quenching mechanism, similar to previous results for other molecular hydrogen evolution and CO_2_ reduction catalysts investigated.^3,39,40^ The strong association of the catalyst and QDs from concentrations of each as low as 1–10 μM is intriguing and may be a more general phenomenon that can facilitate the study and exploitation of QD–catalyst systems.

## Experimental Procedures

### Quantum Dot and Catalyst Synthesis

The CIS QDs were synthesized according to a previously reported procedure with some additional controlled parameters for reproducibility.^51,59^**1** was synthesized according to a previously reported procedure.^24^**2** was synthesized via a novel method based on the previous procedure for macrocyclization. Full details are given in the Supporting Information.

### Photoluminescence Quenching

Samples for PL quenching experiments were prepared with specific relative concentrations of QDs estimated by the absorbance at 405 nm on the basis of the Beer–Lambert law; *A*_QD_ ∝ [QD]. Measurements were taken both in unbuffered conditions (without ascorbate) as well as in 0.1 or 0.5 M ascorbate buffer at pH 4.5 to emulate the conditions used in the photocatalysis experiments. The PL quenching spectra from the conditions investigated are shown in the Supporting Information (Figures S6 and S7). For the highest [QD], a 1 mm cuvette was used in a front-face collection geometry to minimize inner filter effects while enabling monitoring of PL quenching at the higher QD concentrations required for photocatalysis and TA spectroscopy. Note that most of these experiments were performed in the presence of atmospheric oxygen, but the effect of removing oxygen on the quenching mechanism and rate seemed to be minimal (see Figure S16 for oxygen-free comparisons).

### Femtosecond Transient Absorption

The fs-TA experiments were performed using the 800 nm output from a Ti:sapphire-based regenerative amplifier. The pump beam was frequency doubled to 400 nm and attenuated using a neutral density filter prior to reaching the sample (ca. 20 and 40 μJ/cm^2^ for the MIR and UV–vis measurements shown in Figures 6 and 7). The samples were measured with a path length of 1 mm (UV–vis) or 100 μM (MIR). Samples with catalyst and/or buffer were prepared fresh prior to measurement, with the catalyst/QD ratio controlled by the CIS QD absorbance at 405 nm and the estimated ε_405_ from PL quenching modeling. Oxygen-free samples were prepared in a glovebox. See the Supporting Information for more details.

## Supporting Information Available

Additional experimental details, methods, and materials; data from CIS QD characterization, photocatalysis, photoluminescence quenching, absorption titration, femtosecond transient absorption, and cyclic voltammetry (PDF)
