# Supplementary material for: Ultrafast Electron Transfer from CuInS2 Quantum Dots to a Molecular Catalyst for Hydrogen Production: Challenging Diffusion Limitations
Source: ACS Catal. 2024 Mar 4;14(6):4186–201. doi: 10.1021/acscatal.3c06216 (PMC10949191; doi:10.1021/acscatal.3c06216)
Supplement: Supplementary file 1 — cs3c06216_si_001.pdf [file cs3c06216_si_001.pdf]

Supporting Information

Ultrafast Electron Transfer from $CuInS_2$ Quantum Dots to a Molecular Catalyst for Hydrogen Production: *Challenging Diffusion Limitations*

Andrew J. Bagnall,[§ 1,2] Nora Eliasson,[§ 1] Sofie Hansson,[1]
Murielle Chavarot-Kerlidou,[2] Vincent Artero,[2] Haining Tian,[1] and Leif Hammarström*[1]

[1] Department of Chemistry-Ångström Laboratory, Uppsala University, SE-75120 Uppsala, Sweden
leif.hammarstrom@kemi.uu.se

[2] Univ. Grenoble Alpes, CNRS, CEA, IRIG, Laboratoire de Chimie et Biologie des Métaux, 17 rue des Martyrs, F-38054 Grenoble, Cedex, France

The following files are available as Supporting Information.

1. Contents **2**

2. Experimental Details **3**

2.1 Optical Characterization and Photoluminescence Quenching 3

2.2 Photocatalysis 3

2.3 Femtosecond Transient Absorption 3

2.4 Material Characterization 4

3. CIS Quantum Dot Synthesis and Characterization **4**

3.1 Synthesis of Cu-deficient CIS QDs 4

3.2 Powder X-Ray Diffraction 5

3.3 Calculation of CIS Quantum Dot Concentration 5

3.4 Optical Characterization (Absorption and Photoluminescence) 6

4. Gas Chromatography H_2 Production Controls **7**

4.1 Estimate of the H_2 Quantum Yield and QD Excitation Frequency in Full Sunlight 10

5. Photoluminescence Quenching and Titration Controls **11**

5.1 Comment on Photoluminescence Quenching and Spectral Shifts 11

5.2 Photoluminescence Quenching with $HAsc^-$ 11

5.3 Photoluminescence Quenching with Molecular Catalysts 12

5.4 Photoluminescence Quenching with MV^{2+} 15

5.5 Photoluminescence Quenching with Catalysts in Ascorbate buffer 16

5.6 O_2-free Titration Controls of $HAsc^-$ Reduction of Catalysts **1** and **2** 17

5.7 Titration Experiments with all Photocatalytic System Components 19

5.8 Photoluminescence Quenching with Catalysts without O_2 20

6. Femtosecond Transient Absorption (fs-TA) **21**

6.1 Fs-TA of CIS Quantum Dots 21

6.2 Fs-TA of CIS QDs with Catalysts 21

6.3 Fs-TA of CIS Quantum Dots with Methyl Viologen 25

6.4 Fs-TA of CIS Quantum Dots with Varying [**1**] 26

6.5 Fs-TA of CIS/**1**/$HAsc^-$ Samples in the Absence and Presence of Oxygen 27

7. Synthesis and Characterization of Catalyst 2 .... **29**

7.1 Synthesis of **2** 29

7.1.1 Synthesis of tert-Butyl-4-(2,6-diacetyl-pyridin-4-yl)benzoate 29

7.1.2 Synthesis of $[Co(N_4H\text{-}CO_2{}^tBu)Cl_2]^+$ 30

7.1.3 Synthesis of $[Co(N_4H\text{-}CO_2H)Cl_2]^+Cl^-$ 31

7.2 Electrochemical Characterization 32

8. References **32**

2. Experimental Details

MilliQ purified water was used as solvent in all QD and catalyst syntheses and experiments. All reagents used in synthesis were purchased from commercial sources of high purity (Merck / VWR / Fisher Scientific).

2.1 Optical Characterization and Photoluminescence Quenching

Absorption spectra (Varian Cary 50, Agilent Technologies) were obtained using a 1- or 10-mm quartz cuvette. Photoluminescence (PL) and PL excitation (PLE) spectra were recorded on an Edinburgh Instruments fluorimeter in a right-angle (pathlength: 1 cm) or front-face (pathlength: 1 mm) collection geometry. Each individual PL intensity quenching experiment was performed with a constant CIS QD concentration.

Time correlated single photon counting (TCSPC) histograms were recorded using a pulsed diode laser source (Edinburgh Instruments EPL405) operating at 404.7 nm, unless noted otherwise. The time-to-amplitude converter (TAC) was set to a 1 or 5 μs time range over 100000 channels. Colloidal Ludox (IRF) and samples were measured in 1- or 10-mm quartz cuvettes in the forward mode under magic angle polarization. Fluorescence decays were fitted to a sum of three exponentials reconvolved with the IRF.

2.2 Photocatalysis

For each sample, 2 mL aqueous solution of 0.5 M ascorbate buffer at pH 4.5 was made up with the appropriate concentrations of CIS QDs and catalyst in a reaction vessel of total volume 9 mL, leaving a headspace of 7 mL. Once the reagents were all combined in each reaction vessel, they were kept in the dark, crimp-sealed with an airtight septum and purged with argon (the carrier gas for the gas chromatograph) for half an hour. Once purged, the vessels were placed in the photoreactor box and illuminated with 50 mW/cm^2 light intensity from a 5000K cold white LED light source (measured at the samples' positions with a Thorlabs S401C Thermal Power Sensor); similar to the light intensity of one sun across 420–750 nm). Stirring was applied at 300 rpm with identical stirring bars to maintain the consistency of the samples. The temperature inside the box was controlled by its cooling system to keep it at room temperature (20 °C). The samples were removed from the photoreactor box and kept in the dark at the selected time points for gas injections to measure the hydrogen produced, then placed back in the same positions. Injections were made using a 100 μL Hamilton gas syringe, and each sample composition was repeated in triplicate to calculate average values and the standard deviation.

2.3 Femtosecond Transient Absorption

The femtosecond transient absorption (fs-TA) experiments were performed using the output from a Ti:sapphire based regenerative amplifier with integrated oscillator and pump lasers (Libra, Coherent). The 800 nm output (3 kHz, ~1.5 mJ/pulse) was split into a pump and probe which were directed toward the UV–vis–NIR/MIR sample chambers (TAS, Newport Corp./Helios IR, Ultrafast Systems). The pump beam was frequency doubled to 400 nm (second harmonic generation) or passed through optical parametric amplifiers (TOPAS-prime/TOPAS-NIRUVis, Light Conversion) for alternative wavelengths (350-600 nm). The pump was passed through a phase locked chopper to halve the repetition rate (3 to 1.5 kHz) and attenuated using a neutral density filter prior to reaching the sample. The UV-Vis/NIR super continuums were generated by focusing ~1 μJ of the fundamental onto calcium fluoride (CaF_2) and yttrium aluminum garnet (YAG) crystals, respectively. The broadband UV-Vis/NIR probe was subsequently focused onto an optical fiber coupled to a MS260i spectrometer (Newport Corp.) after passing through the sample (1 mm pathlength). Alternatively, the probe for MIR measurements (ν_{probe}: 2600 cm^{-1} / λ_{probe}: 3850 nm) was generated through an optical parametric amplifier (TOPAS-prime, Light Conversion) coupled with frequency mixers for difference-frequency generation

and recorded on a multichannel spectrometer with a MCT sensor array (Helios IR, Ultrafast Systems). Dual channel detection (a reference probe) was used to monitor intensity fluctuations in the probe. A sample cell with a 100 μm Teflon spacer between two CaF_2 windows was used for MIR measurements. Individual scans were analyzed carefully for inconsistencies (*e.g.* due to photodamage) prior to analysis.

2.4 Material Characterization

A Zetasizer Nano S (Malvern, U.K) and a Zetasizer Nano-ZS (Malvern, U.K) was used to determine the hydrodynamic diameter and the surface ζ-potential of the CIS quantum dots dispersed in water. The powder X-ray diffraction (p-XRD) pattern was obtained using a Simons D5000 diffractometer (λ = 1.5418 Å, 45 kV, 40 mA) with a 20–80 2θ scan range and 0.02° step size.

3. CIS Quantum Dot Synthesis and Characterization

3.1 Synthesis of Cu-deficient CIS QDs

Hybrid-passivated CIS QDs were prepared following the literature procedure previously reported by Huang *et al.* and dispersed after centrifugation in 1.4 mL MilliQ water before further dilution.[1] Specific synthesis details are included here to aid in reproducibility.[2] The following stocks solutions were made up fresh each time for use in the synthesis:

- $CuCl_2$ dihydrate (3.4 mg, 0.020 mmol, 1.0 mM) in water (20 mL)
- $InCl_3$ anhydrate (221 mg, 1.0 mmol, 1.0 M) in absolute ethanol (1 mL)
- Trisodium citrate dihydrate (294 mg, 1.0 mmol, 0.40 M) in water (2.5 mL)
- Na_2S (78 mg, 1.0 mmol, 1.0 M) in water (1 mL)
- Tetra-*n*-butylammonium iodide (TBAI, 20 mg, 0.054 mmol, 11 mM) in water (5 mL)

A clean 2-necked 100 mL round bottom flask with both necks sealed by appropriate size rubber septa was used as the reaction vessel and heated using an oil heat bath on a hotplate, with the temperature controlled by a thermocouple in the oil bath. Aluminum foil was wrapped around the top of the reaction vessel once all reagents were added to minimize heating time or any chances of photobleaching. Light exposure was minimized. Constant stirring at 300 rpm with a 3 cm long cylindrical stir bar was maintained before and during both the reaction and workup. The temperature was set to warm to 40 °C during insertion of the reagents; a needle was left in the secondary neck septum to allow for pressure equilibration with the atmosphere. The reagents were promptly added one after another through the main neck in the following order: The capping ligand L-cysteine (2.4 mg, 20 μmol) was added as a solid, followed by 17 mL of MilliQ water. From stock solutions were added: 6.0 mL of $CuCl_2$ dihydrate/water (6.0 μmol), 0.040 mL $InCl_3$ anhydrate/ethanol (40 μmol), 0.40 mL trisodium citrate dihydrate/water (160 μmol). Then, checking that the bath temperature had reached 40 °C, 0.62 mL of Na_2S/water (620 μmol) was injected all at once, causing an immediate color change to orange, and the thermocouple-controlled hotplate temperature was set to 95 °C. The main neck was sealed and the needle was removed for the further heating. Typically, it took ca. 45 mins to reach 95 °C, the temperature was monitored and recorded at multiple time points to compare for consistency between syntheses. Once 95 °C was reached, the pressure was briefly reequilibrated with the atmosphere (by temporarily reinserting the needle) and the reaction was left to proceed for 40 mins, monitoring the temperature to keep it at 95 ± 2 °C. Workup: After this time, the reaction vessel was removed from the oil bath and cooled to room temperature with an ice water bath. Once room temperature was reached, 1.0 mL TBAI/water (0.011 mmol) was added dropwise and the mixture was left stirring at room temperature for a further 30 mins. Then, the reaction mixture (~25 mL) was partitioned between two 50 mL centrifuge tubes and absolute ethanol was added as an antisolvent to reach volumes of about 48 mL in each, ensuring that their weights were equal to within 1 mg. The mixture was centrifuged at 9600 rpm for 15 minutes. Then, the supernatant was removed to leave the precipitated CIS QD product. By default, this was redispersed

into 1.4 mL water total, giving a solution with an absorbance of ~10 per 1 cm. For characterization, these QD dispersions were diluted by about 200 times to reach 0.07 absorbance at 405 nm.

3.2 Powder X-Ray Diffraction

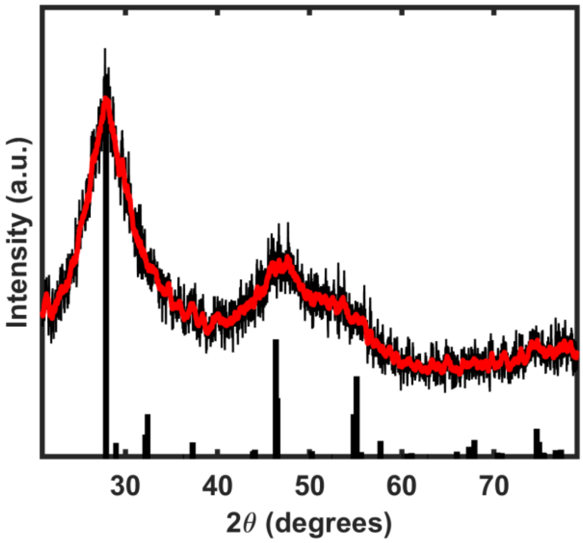

Figure S1. a) Powder X-ray diffraction (p-XRD) of the CIS quantum dots (black: raw data, red: smoothed using moving average filter). The corresponding planes of the chalcopyrite structure are indicated in black bars (JCPDS entry 85-1575).

3.3 Calculation of CIS Quantum Dot Concentration

A rough estimation of the CIS QD concentration was calculated from the hydrodynamic radius ($r_h \approx 1.8$ nm) obtained from DLS and from the lattice parameters determined by p-XRD. The estimated QD volume ($V_{QD} \approx 22$ nm^3) and chalcopyrite unit cell volume calculated from Eq. S1 (a: 5.52 Å, c: 11.1 Å) were used to determine the total number of unit cells per QD (eq. S2).

$$V_{uc} = a^2 c \times \sin(60) = 0.293 \text{ nm}^3 \quad \text{(S1)}$$

$$N_{uc/QD} = \frac{V_{QD}}{V_{uc}} = 77 \quad \text{(S2)}$$

The synthesized CIS QDs are prepared Cu-deficient, we therefore use the amount of In precursor (n_{In} = 0.04 mmol) to estimate the moles of QDs obtained from synthesis (Eq. S3), assuming that each unit cell is occupied by one In on average.

$$n_{QD} = \frac{n_{In}}{N_{In/QD}} = 5 \times 10^{-7} \text{ mol} \quad \text{(S3)}$$

The synthesized QDs were redispersed in 1.4 mL of water, which gives a crude $[QD]_{stock}$ estimate of ~4×10^2 µM. The QD stock solution was typically diluted ~200 times to obtain an absorption of 0.07 at 405 nm (1 cm cuvette), which corresponds well (within a factor of two) with the [QD] estimated from the PL quenching modeling.

3.4 Optical Characterization (Absorption and Photoluminescence)

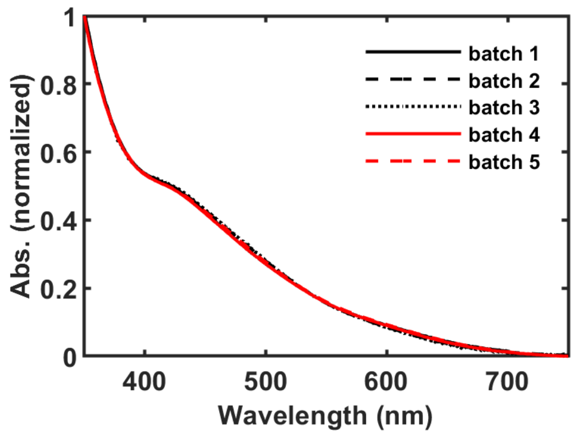

Figure S2. a) Absorption spectra for five different CIS QD batches. The spectra have been normalized at 350 nm. The different batches show minor differences in concavity across the absorption band.

Table S1. Photoluminescence (PL) lifetimes of CIS QDs measured by time-correlated single-photon counting. Fitted with a sum of three exponential functions reconvolved with the instrument response function (Figure S8c) at different wavelengths across the PL. All samples were diluted to have an absorbance of 0.07 at 405 nm in a 1 cm cuvette (solvent: deionized water).

Exn (nm)	Emn (nm)	τ_1 (ns)	% τ_1	τ_2 (ns)	% τ_2	τ_3 (ns)	% τ_3
470	770	23 ± 1	*63% ± 6%*	130 ± 4	*29% ± 1%*	440 ± 9	*7.4% ± 0.4%*
470	700	25 ± 1	*63% ± 4%*	130 ± 4	*30% ± 1%*	420 ± 8	*7.1% ± 0.4%*
470	670	23 ± 1	*60% ± 4%*	130 ± 3	*32% ± 1%*	400 ± 8	*7.6% ± 0.4%*
470	570	13 ± 1	*59% ± 2%*	61 ± 2	*37% ± 1%*	290 ± 6	*4.5% ± 0.2%*
470	550	9.8 ± 0.7	*59% ± 11%*	53 ± 1	*39% ± 2%*	280 ± 10	*2.2% ± 0.1%*
405	770	29 ± 1	*64% ± 4%*	160 ± 0	*30% ± 1%*	490 ± 7	*6.3% ± 0.2%*
405	700	27 ± 1	*66% ± 4%*	150 ± 4	*29% ± 1%*	460 ± 10	*5.3% ± 0.3%*
405	670	17 ± 0	*71% ± 1%*	140 ± 1	*26% ± 0%*	490 ± 0	*3.1% ± 0.0%*
405	570	22 ± 1	*67% ± 5%*	99 ± 3	*28% ± 1%*	360 ± 10	*4.3% ± 0.3%*
405	550	19 ± 1	*61% ± 6%*	68 ± 2	*35% ± 2%*	310 ± 8	*3.4% ± 0.2%*

4. Gas Chromatography H_2 Production Controls

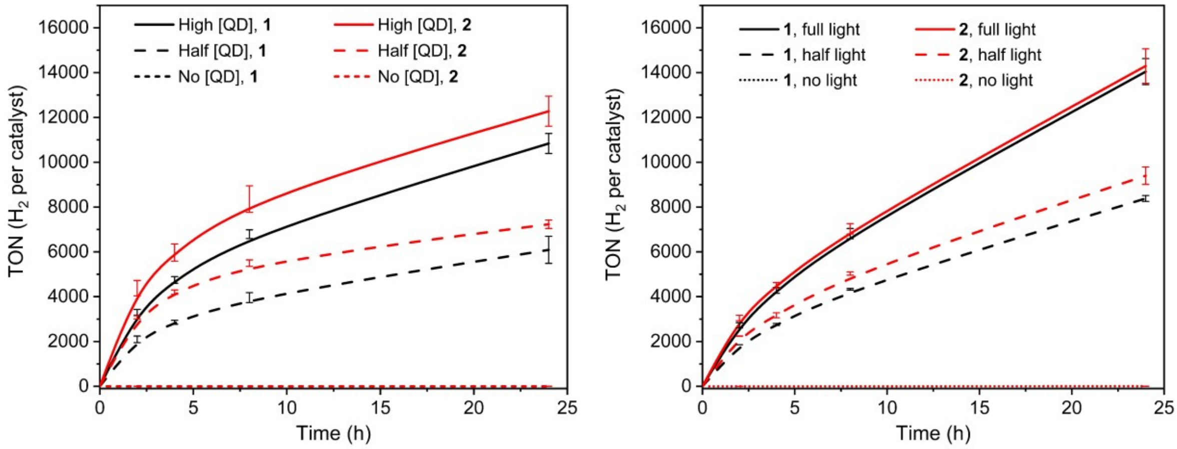

Figure S3. TON of H_2 produced per catalyst measured by gas chromatography against irradiation time. Buffer solution: 0.5 M H_2Asc/NaHAsc, pH = 4.5 (2 mL solution, 7 mL headspace). Left: Different concentration of CIS QDs with absorbances at 405 nm (1 cm pathlength) of 0.35 for 'high [QD]', 0.18 for 'half [QD]' and 0 for 'no [QD]': estimated as 6.5 µM, 3.3 µM and 0 µM, respectively. A constant irradiance of 50 mW/cm^2 over 420–750 nm and concentration of either catalyst of 1 µM. Right: Different irradiances (50 mW/cm^2, 25 mW/cm^2 and no light) for a constant QD absorbance at 405 nm of 0.35 across 1 cm (estimated 6.5 µM) and concentration of either catalyst of 1 µM. Note that a different CIS QD batch was used for the set of experiments in each figure, and the variation in total TON over 24 hours under the same conditions can be ascribed to interbatch dissimilitude.

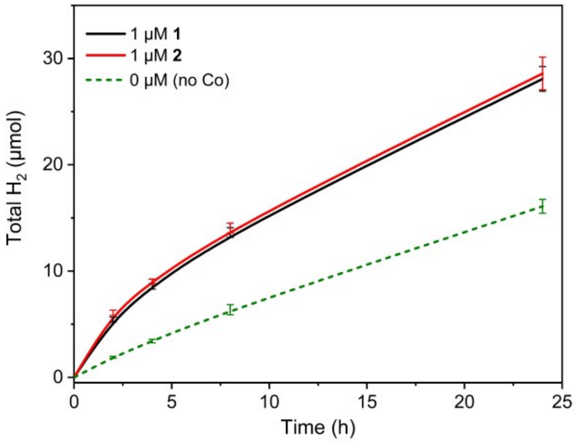

Figure S4. Total H_2 production measured by gas chromatography against illumination time for 1 µM of either catalyst versus the catalyst-free control. Buffer solution: 0.5 M H_2Asc/NaHAsc, pH = 4.5 (2 mL solution, 7 mL headspace). QD absorbance at 405 nm across 1 cm of 0.35, corresponding to ca. 6.5 µM QDs. Constant irradiance of 50 mW/cm^2. Note that the same QD batch was used as for Figure S3 (right) for direct comparison. Observe that the hybrid passivated CIS QDs by themselves are substantially catalytically active for the hydrogen evolution reaction under these conditions (this property is known for ZnS-shell and shell-less CIS QDs in literature[3–5]), producing about half as much H_2 as CIS QDs with either catalyst.

Table S2. Hydrogen measurement data from gas chromatography injections from photocatalysis experiments varying the concentration of each catalyst (see main text Figure 4).

	10 µM **1**	5 µM **1**	1 µM **1**	10 µM **2**	5 µM **2**	1 µM **2**
Time (h)	***H_2 in headspace (µmol)***					
0	0 ± 0	0 ± 0	0 ± 0	0 ± 0	0 ± 0	0 ± 0
2	5.4 ± 0.2	5.2 ± 0.4	3.8 ± 0.2	6.3 ± 0.3	6.1 ± 0.1	4.6 ± 0.3
4	7.1 ± 0.4	7.0 ± 0.5	5.5 ± 0.4	8.0 ± 0.4	7.7 ± 0.2	6.2 ± 0.4
8	10.0 ± 0.7	9.5 ± 0.8	8.6 ± 0.4	11.0 ± 0.4	11.0 ± 0.2	9.0 ± 0.5
24	18.0 ± 2.0	16.0 ± 0.9	17.0 ± 2.0	19.0 ± 0.6	18.0 ± 0.5	16.0 ± 0.8
Time (h)	***TON of H_2 per catalyst***					
0	0 ± 0	0 ± 0	0 ± 0	0 ± 0	0 ± 0	0 ± 0
2	270 ± 10	520 ± 40	1900 ± 100	320 ± 20	610 ± 10	2300 ± 100
4	360 ± 20	700 ± 50	2800 ± 200	400 ± 20	770 ± 20	3100 ± 200
8	500 ± 30	950 ± 80	4300 ± 200	570 ± 20	1100 ± 20	4500 ± 200
24	900 ± 80	1600 ± 90	8400 ± 900	950 ± 30	1800 ± 50	8000 ± 400

Table S3. Highest H_2 TOFs, derived from Table S2 (1 µM of either catalyst, est. 6.5 µM CIS QDs).

	1 µM **1**	1 µM **2**
Time (h)	***Average overall TOF of H_2 per catalyst (s^{-1})***	
0	0 ± 0	0 ± 0
2	0.260 ± 0.016	0.320 ± 0.019
4	0.190 ± 0.013	0.220 ± 0.015
8	0.150 ± 0.008	0.160 ± 0.009
24	0.098 ± 0.010	0.093 ± 0.005

Table S4. Hydrogen measurement data from gas chromatography injections from photocatalysis experiments varying the concentration of CIS QDs (see Figure S3, Left).

	**1**, high [QD]	**1**, half [QD]	**1**, no [QD]	**2**, high [QD]	**2**, half [QD]	**2**, no [QD]
Time (h)	***H_2 in headspace (µmol)***					
0	0 ± 0	0 ± 0	0 ± 0	0 ± 0	0 ± 0	0 ± 0
2	6.6 ± 0.3	4.2 ± 0.3	0.0003 ± 0.0003	8.8 ± 0.7	6.2 ± 0.1	0.0031 ± 0.0040
4	9.5 ± 0.3	5.7 ± 0.2		12.0 ± 0.8	8.4 ± 0.2	
8	14.0 ± 0.4	7.9 ± 0.4		17.0 ± 1.0	11.0 ± 0.3	
24	22.0 ± 0.9	12.0 ± 1.0	0.0021 ± 0.0020	25.0 ± 1.0	14.0 ± 0.4	0.0023 ± 0.0020
Time (h)	***TON of H_2 per catalyst***					
0	0 ± 0	0 ± 0	0 ± 0	0 ± 0	0 ± 0	0 ± 0
2	3300 ± 100	2100 ± 100	0.13 ± 0.10	4400 ± 300	3100 ± 70	1.5 ± 2.0
4	4700 ± 100	2900 ± 90		6000 ± 400	4200 ± 100	
8	6800 ± 200	4000 ± 200		8400 ± 600	5500 ± 100	
24	11000 ± 400	6100 ± 600	1.1 ± 1.0	12000 ± 700	7200 ± 200	1.2 ± 0.8

Table S5. Hydrogen measurement data from gas chromatography injections from photocatalysis experiments varying the irradiance (see Figure S3, Right).

	**1**, full light	**2**, full light	**1**, half light	**2**, half light	**1**, no light	**2**, no light
Time (h)	***H_2 in headspace (μmol)***					
0	0 ± 0	0 ± 0	0 ± 0.0	0 ± 0.0	0 ± 0	0 ± 0
2	5.4 ± 0.2	6.1 ± 0.2	3.7 ± 0.0	4.5 ± 0.0	0.0002 ± 0.0001	0.0004 ± 0.0003
4	8.6 ± 0.3	9.0 ± 0.2	5.5 ± 0.1	6.3 ± 0.2		
8	14.0 ± 0.5	14.0 ± 0.5	8.7 ± 0.1	10.0 ± 0.1		
24	28.0 ± 1.0	29.0 ± 2.0	17.0 ± 0.3	19.0 ± 0.8	0.0079 ± 0.0050	0.0120 ± 0.0020
Time (h)	***TON of H_2 per catalyst***					
0	0 ± 0	0 ± 0	0 ± 0	0 ± 0	0 ± 0	0 ± 0
2	2700 ± 100	3000 ± 100	1900 ± 1	2200 ± 5	0.12 ± 0.07	0.2 ± 0.1
4	4300 ± 100	4500 ± 100	2800 ± 50	3200 ± 100		
8	6800 ± 200	7000 ± 200	4300 ± 40	5000 ± 70		
24	14000 ± 600	14000 ± 800	8400 ± 100	9400 ± 400	3.9 ± 3.0	6.2 ± 1.0

Table S6. Hydrogen measurement data from gas chromatography injections from photocatalysis experiments to control for the presence of catalyst (see Figure S4).

	**1**, full light	**2**, full light	No catalyst, full light
Time (h)	***H_2 in headspace (μmol)***		
0	0 ± 0	0 ± 0	0 ± 0.0
2	5.4 ± 0.2	6.1 ± 0.2	1.9 ± 0.1
4	8.6 ± 0.3	9.0 ± 0.2	3.4 ± 0.2
8	14.0 ± 0.5	14.0 ± 0.5	6.4 ± 0.5
24	28.0 ± 1.0	29.0 ± 2.0	16.0 ± 0.7
Time (h)	***TON of H_2 per catalyst***		
0	0 ± 0	0 ± 0	*n/a*
2	2700 ± 100	3000 ± 100	*n/a*
4	4300 ± 100	4500 ± 100	*n/a*
8	6800 ± 200	7000 ± 200	*n/a*
24	14000 ± 600	14000 ± 800	*n/a*

4.1 Estimate of the H_2 Quantum Yield and QD Excitation Frequency in Full Sunlight

The approximate initial quantum yields of hydrogen production of 2–3% under white-light conditions were determined for the photocatalysis experiments in Figures 4, S3 & S4 as follows: The irradiance from the 5000K cold LED bulb from 420–750 nm was measured at the sample position by a thermal power sensor as 50 mW cm^{-2}. This is similar to the power density of a full sun (AM1.5) in the visible spectral range (100 mW cm^{-2} in total,[6] of which ca. 50% is in the visible). We estimate a flux of 4×10^{16} attainable photons cm^{-2} s^{-1}, for which the average photon energy is 240 kJ mol^{-1}, corresponding to an effective absorbed irradiance of 16 mW cm^{-2} (~32%). From the geometry of the reaction vessel (diameter 1.9 cm), the average pathlength through the solution is approximated as 1.5 cm. For the solution volumes of 2.0 mL, the area of irradiance is then approximated as 1.3 cm^2. This gives a rate of photon absorption (excitation frequency) of 7 s^{-1} per QD under these conditions. Over 2 hours, this is 50,000 photons per QD. Considering that two photons are required per H_2 molecule and that the extrapolated solution concentration of QDs was ~6.5 μM, initial (0–2 hours) quantum yields of hydrogen production of 2–3% were calculated under the optimal conditions for TON per QD (i.e. with 10 μM of either co-catalyst) across the independent photocatalysis measurements of different samples. It was noted that the quantum yields calculated for the controls under 25 mW cm^{-1} irradiance were also within this range, and that different co-catalyst:QD ratios had little noticeable effect.

5. Photoluminescence Quenching and Titration Controls

5.1 Comment on Photoluminescence Quenching and Spectral Shifts

We note that quenching by the catalysts leads to an apparent blue shift of the remaining PL, opposite to the effect with ascorbate (see Figure S5-7). This can reflect the quenching of different electronic states, where the adsorbed catalyst preferentially quenches lower energy transitions *e.g.* coupled to surface states; however, we consider the majority of the PL in the presence of the catalyst to be from QDs with no catalyst bound (ideal quenching). If the effect is due to a QD size distribution, this would imply that the catalyst preferentially quenches the subset of larger QDs, which is unintuitive from driving force perspective but consistent with their higher surface area. A shift in PL energies from a strong QD-catalyst interaction is also conceivable. We note that a similar PL blueshift and nonlinear dependence of the PL ratio (F_0/F) with concentration was observed by Natali and coworkers (2022) in quenching studies using core-shell CIS/ZnS QDs with different cobalt polypyridine complexes.[7] These analogous observations may suggest that the behavior is general to similar systems.

5.2 Photoluminescence Quenching with $HAsc^{-}$

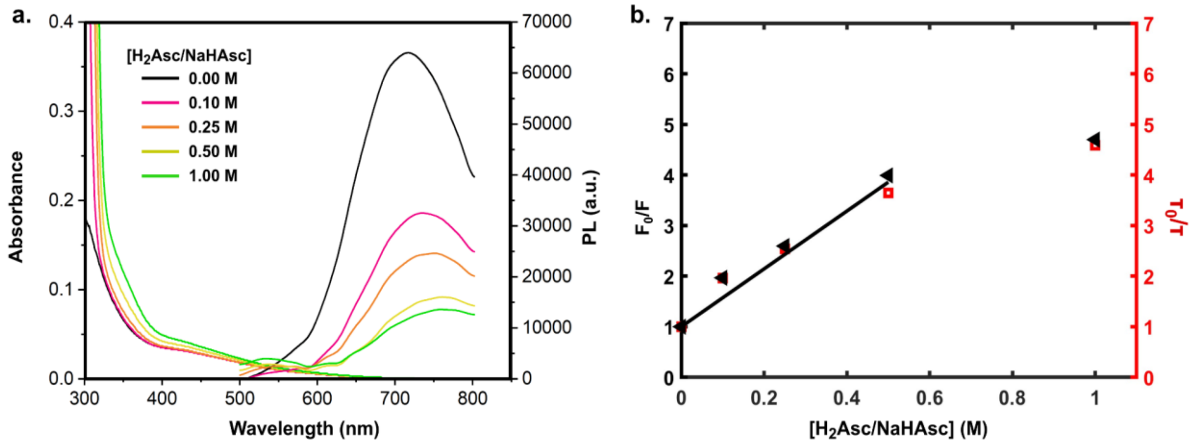

Figure S5. Photoluminescence (PL) quenching of the CIS quantum dots by the sacrificial electron donor ascorbate (at a concentration such that the QDs had an absorbance of 0.36 in water at 405 nm for a pathlength of 1 cm) with increasing concentrations of the ascorbate buffer (pH = 4.5). **a)** UV-Vis absorption spectra (left) of the solution corresponding to each PL spectra (right). PL from excitation at 405 nm. Inset is the total solution concentration of the ascorbate buffer. At pH = 4.5, the concentration of the sacrificial electron donor, ascorbate, is approximately 70% of the total concentration of $H_2Asc/NaHAsc$ (pK_{a1} ~ 4.2). Cuvette pathlength for quenching experiment: 1 mm. **b)** F_0/F (black triangles) and τ_0/τ (red squares) against buffer concentration, derived from the PL spectra shown in a) and from the integrated PL decay profiles measured by time-correlated single photon counting (TCSPC). A linear fit (black line) applied to the region up to 0.5 M $H_2Asc/NaHAsc$ concentration gives a slope of $K_{SV} = 6.2 \pm 0.4\ M^{-1}$. This is a *ca.* 25 times smaller value than recently reported for CIS/ZnS QDs which may be attributed to the absence of a ZnS shell in the present case).[7]

5.3 Photoluminescence Quenching with Molecular Catalysts

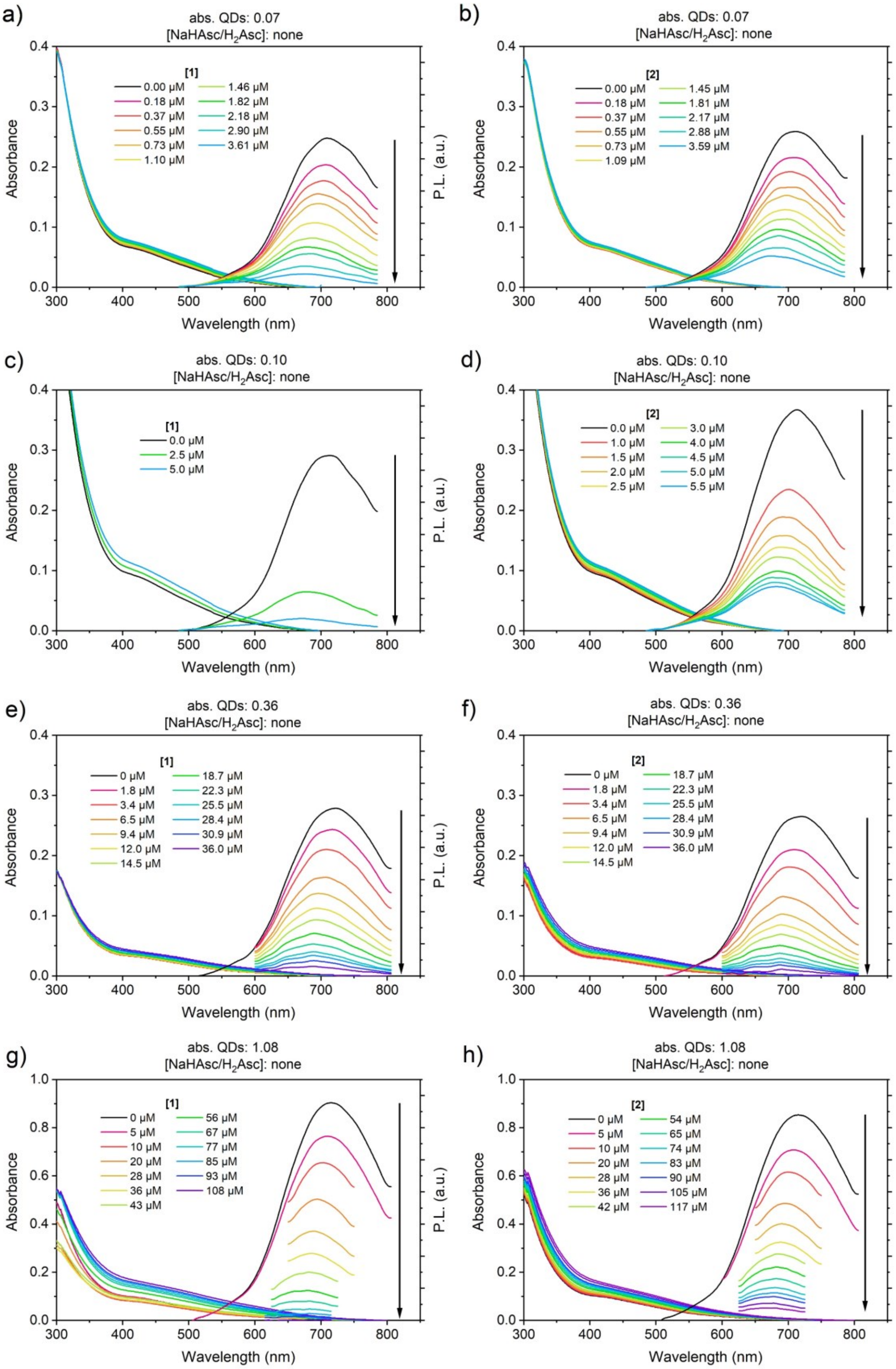

Figure S6. Spectra of the PL quenching of the CIS QDs in H_2O by increasing concentrations of each catalyst (inset). UV-Vis spectra (left) corresponding to each PL spectrum (right) from excitation at 405 nm. Cuvette pathlength: 1 cm for QD absorbance of 0.07–0.10 (right angle), 1 mm for QD absorbance of 0.36-1.08 (front-face). 'Abs. QDs' refers to their absorbance at 405 nm in a 1 cm cuvette to quantify their concentration.

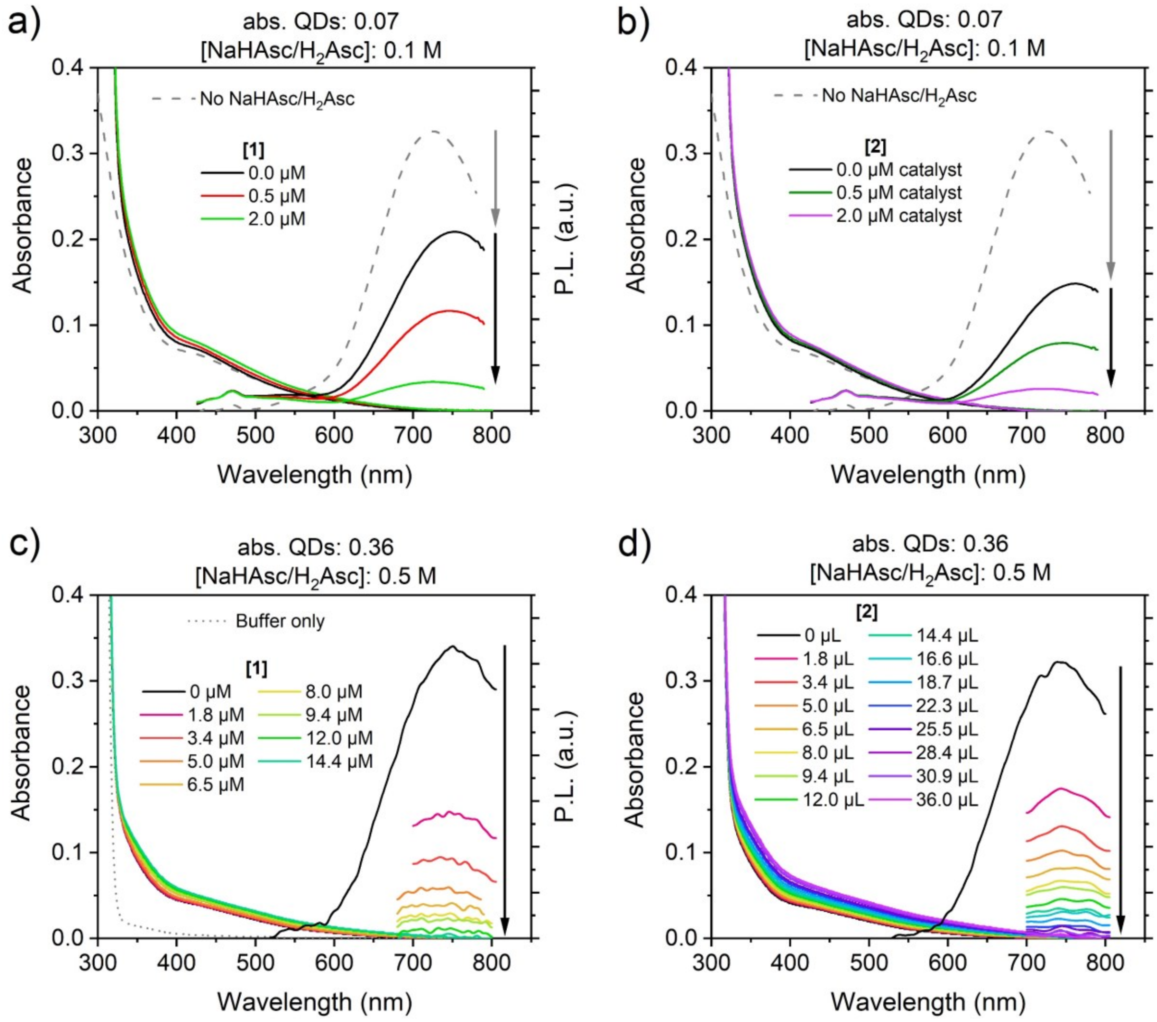

Figure S7. PL intensity quenching of the CIS QDs in buffer (H_2Asc/NaHAsc, pH = 4.5) by increasing concentrations of each catalyst (inset). UV-Vis spectra (left) corresponding to each PL spectrum (right) from excitation at 405 nm. Cuvette pathlength: 1 cm for QD absorbance of 0.07 (right angle), 1 mm for QD absorbance of 0.36 (front-face). 'Abs. QDs' refers to their absorbance at 405 nm in a 1 cm cuvette to quantify their concentration.

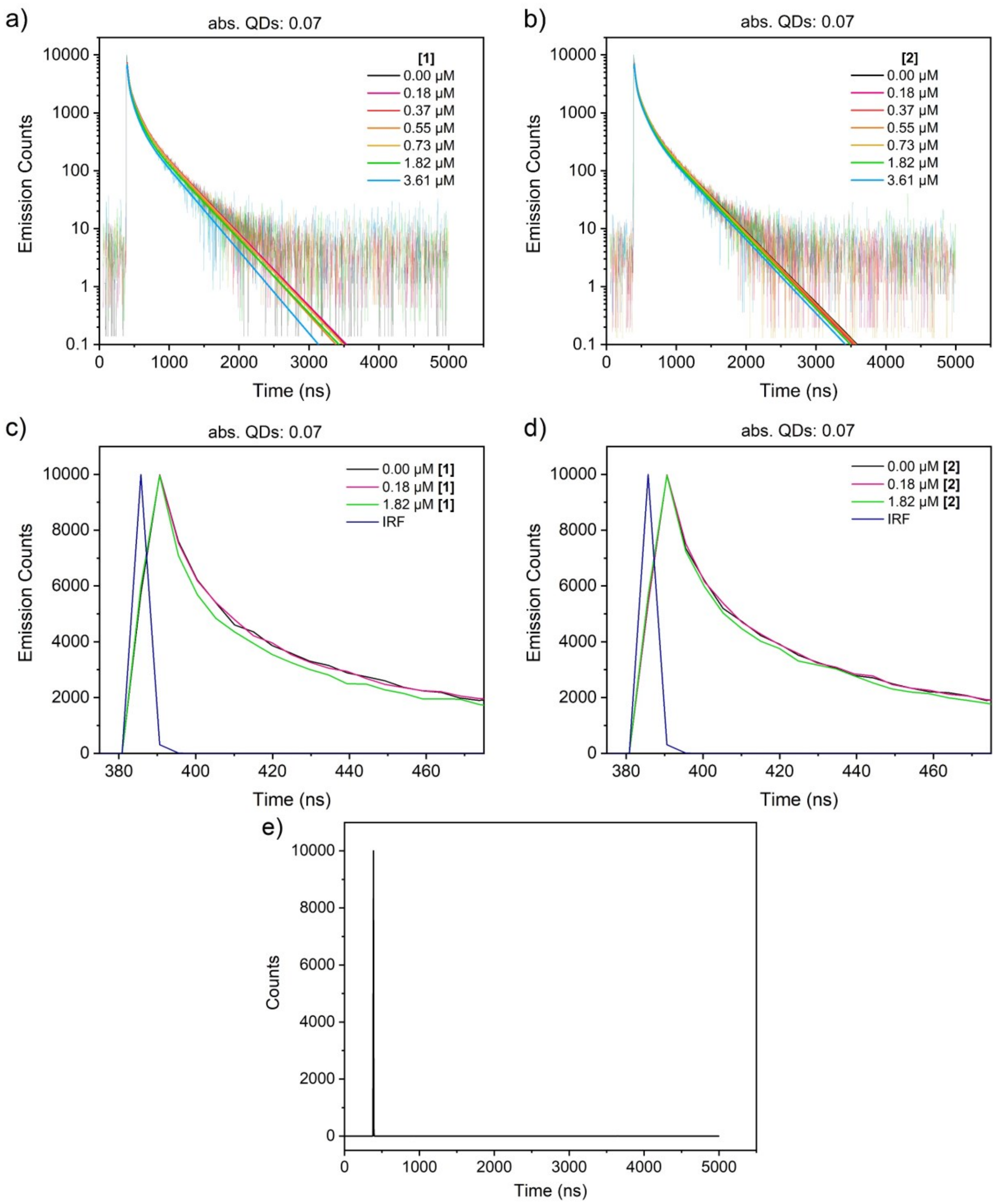

Figure S8. TCSPC traces and triexponential fits of counts on logarithmic scale for the same samples as in Figure S6a–b: A CIS QD sample diluted to have an absorbance of 0.07 at 405 nm (estimated [QD]: approximately 1 μM) with increasing concentration of either **a) 1** or **b) 2**. Values for the fits with 0 μM or 3.61 μM catalyst: **a)** No catalyst: 19 ns (57%), 98 ns (33%), 340 ns (10%); with 3.61 μM **1**: 16 ns (58%), 82 ns (33%), 306 ns (10%); **b)** No catalyst: 19 ns (57%), 102 ns (33%), 355 ns (10%); with 3.61 μM **2**: 19 ns (61%), 100 ns (30%), 341 ns (9%). Selected traces in a linear scale over the shorter delay times for **c) 1** or **d) 2** are shown against the instrument response function, which was recorded under same conditions and with a very dilute solution of colloidal silica instead of sample. **e)** Instrument response function plotted separately on a linear scale over the full delay time. PL excitation wavelength for TCSPC: 470 nm. Cuvette pathlength: 1 cm. Solvent: deionized water. Note the lack of a significant impact upon the decay profile or lifetimes with increasing catalyst concentration.

Table S7. Estimated CIS QD concentrations (column 5) from exponential fits to the PL intensity ratios (F_0/F) shown in Figure 5 in the main article. The bottom four rows show the results from experiments performed in buffer (H_2Asc/NaHAsc, 0.1 M/0.5 M, pH = 4.5). Note that the $[QD]_{ln(F0/F)=1}$ estimated from the PL maximum (shown in table) and integrated PL spectra yield similar results. From the A_{405} = 0.1 condition with **1** and no ascorbate, an $\varepsilon_{405\ nm}$ of 54000 $M^{-1}\ cm^{-1}$ is obtained for the CIS QDs.

**$Abs_{405\ nm}$ (rel. [QD])**	**Sample**	**$\bar{n}$**	**R^2**	**$[QD]_{ln(F0/F)=1}$ (μM)**	**Avg. [QD] (μM)**
0.07	CIS / **1**	690171 × [Co]	0.9912	1.4	1.7
0.07	CIS / **2**	496042 × [Co]	0.9379	2	
**0.1**	**CIS / 1**	**539125 × [Co]**	**0.9941**	**1.9**	2.5
0.1	CIS / **2**	325154 × [Co]	0.9354	3.1	
0.36	CIS / **1**	76441 × [Co]	0.9975	13.1	12.2
0.36	CIS / **2**	87914 × [Co]	0.9948	11.4	
1.08	CIS / **1**	34834 × [Co]	0.9939	28.7	34.9
1.08	CIS / **2**	24341 × [Co]	0.9945	41.1	
0.07	CIS / **1** (H_2Asc)	927618 × [Co]	0.9880	1.1	1.1
0.07	CIS / **2** (H_2Asc)	897994 × [Co]	0.9759	1.1	
0.36	CIS / **1** (H_2Asc)	295203 × [Co]	0.9877	3.4	5.5
0.36	CIS / **2** (H_2Asc)	131814 × [Co]	0.9760	7.6	

5.4 Photoluminescence Quenching with MV^{2+}

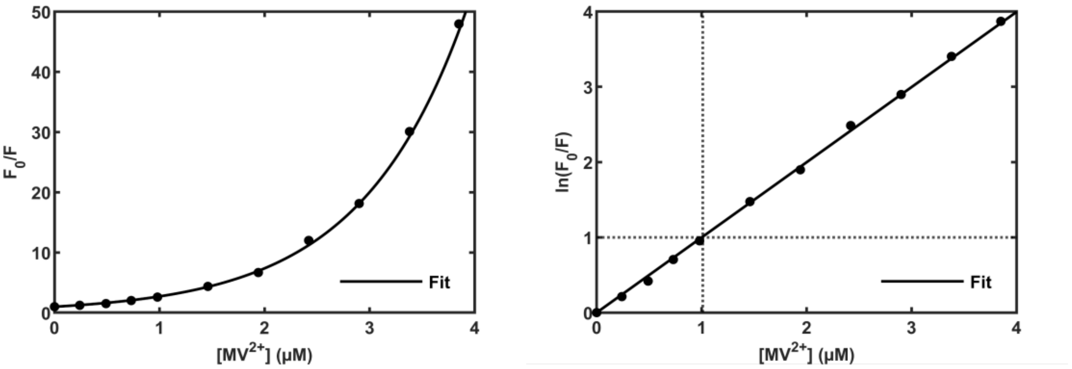

Figure S9. Photoluminescence (PL) quenching of CIS QDs with methyl viologen, MV^{2+}. The plots show **a)** the PL intensity ratio, F_0/F, and **b)** the linearized PL ratio, $\ln(F_0/F)$, against $[MV^{2+}]$. Solid lines represent exponential fits and the dashed lines in b) indicate the $[MV^{2+}]$ where $\ln(F_0/F) = 1$. Under the presumption of negligible free MV^{2+}, this yields a QD concentration estimation of $[QD]_{ln(F0/F)=1} \approx 1$ μM for an absorbance of 0.07 at 405 nm in a 1 cm cuvette. Adapted from ref.[8]

5.5 Photoluminescence Quenching with Catalysts in Ascorbate buffer

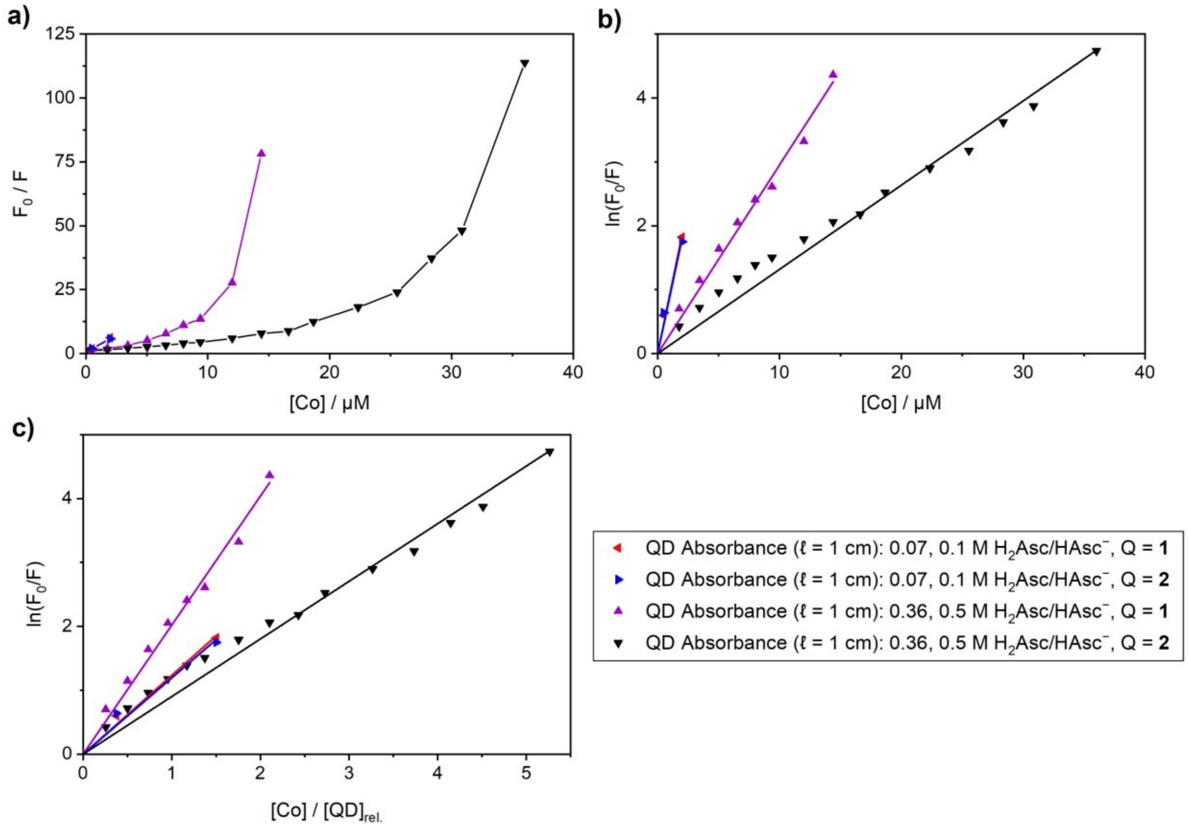

Figure S10. Photoluminescence quenching of the CIS QD system in ascorbate buffer (pH = 4.5, 0.1 M or 0.5 M) with **1** or **2** for different QD concentrations represented by the absorbance at 405 nm in a 1 cm cuvette, as indicated in the legend. The plots show: **a)** F_0/F against the added catalyst concentration, [Co], **b)** ln(F_0/F) against [Co], **c)** ln(F_0/F) against [Co]/[QD]$_{rel.}$, where [QD]$_{rel.}$ is the concentration relative to that of the sample with A_{405} = 0.10. Solid lines in b–c represent fits.

5.6 O_2-free Titration Controls of $HAsc^-$ Reduction of Catalysts **1** and **2**

O_2-free titration experiments followed by UV-Vis spectroscopy (Figures S11–12) show linear growth of Co^{II} peaks and simultaneous fall of Co^{III} peaks, indicating that the Co^{III} form of both catalysts is reduced by ascorbate to Co^{II}. Titrating approximately 0.5 equivalents of NaHAsc/H_2Asc results in the complete conversion of Co^{III} to Co^{II}; beyond 0.5 equivalents, no further change is observed, indicating that ascorbate/ascorbic acid is acting as an overall 2 electron donor for either catalyst in the absence of photosensitizer.

This is consistent with reverse titration experiments (Figure S13), where we observe a linear growth of the absorbance peaks corresponding to Co^{II} of **1** for up to 2 equivalents of **1** per ascorbate/ascorbic acid, beyond which the shape of the absorption profile begins to change and the growth slows down significantly, indicating that newly added Co^{III} (**1**) is no longer being reduced to Co^{II}. Hence, the reduction process is irreversible and does not exhibit hysteresis, and both species are stable in the presence of each other under QD-free conditions.

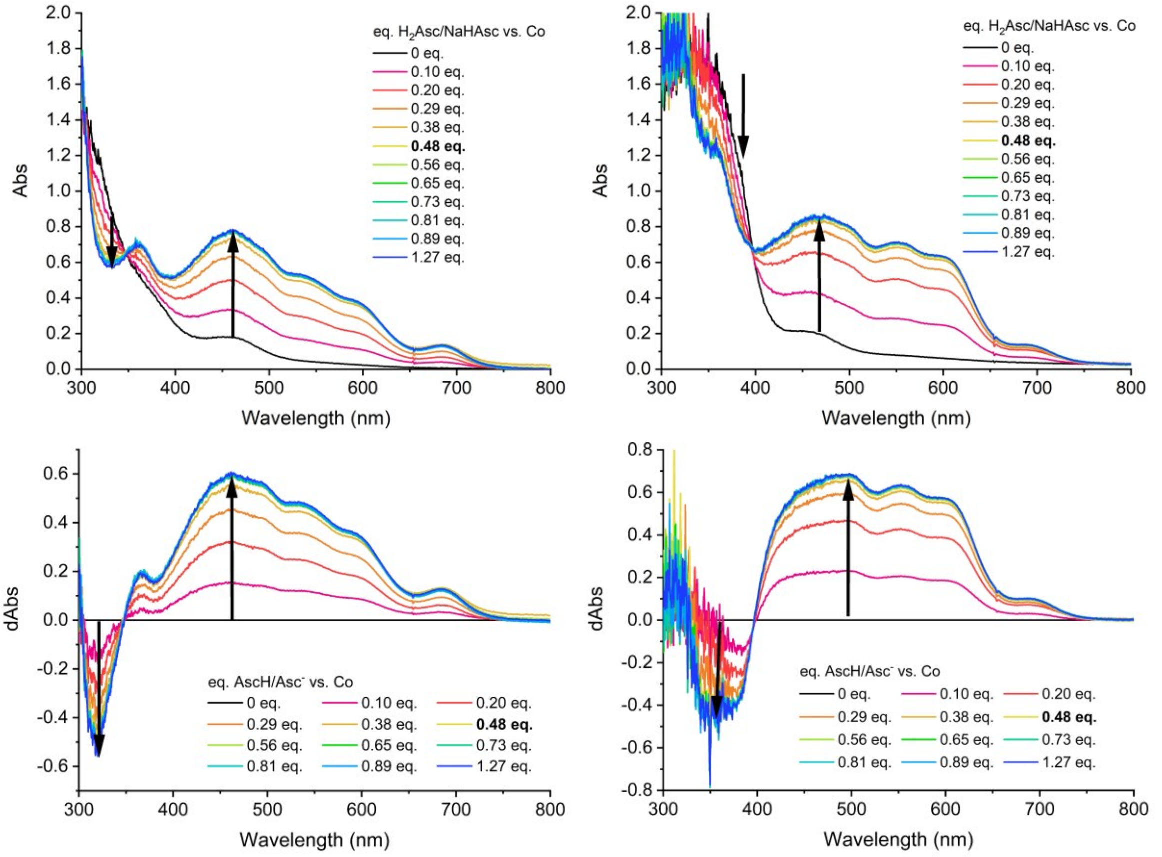

Figure S11. Titration absorption spectra (top) and change in absorbance (bottom) from gradual addition of **1** (left) or **2** (right) in ascorbate buffer (pH = 4.5, 3.1 mM NaHAsc, 1.5 mM H_2Asc) into aqueous solutions of only **1** or **2**, respectively. Constant [Co] of 0.60 mM. Performed in glovebox (oxygen-free) conditions. Note the stable isosbestic points at 347 nm and 397 nm, respectively.

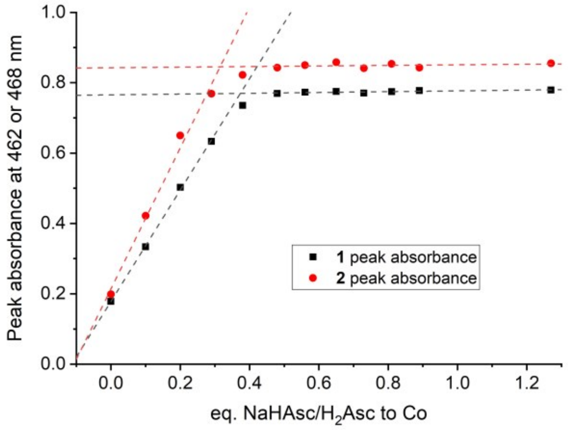

Figure S12. Plots of the peak absorbance at 462 nm (for **1**) or 468 nm (for **2**) from Figure S11 with increasing ascorbate concentration under conditions of constant catalyst concentration and no CIS QDs. The dashed lines over the first four datapoints are fits of the linear growth region.

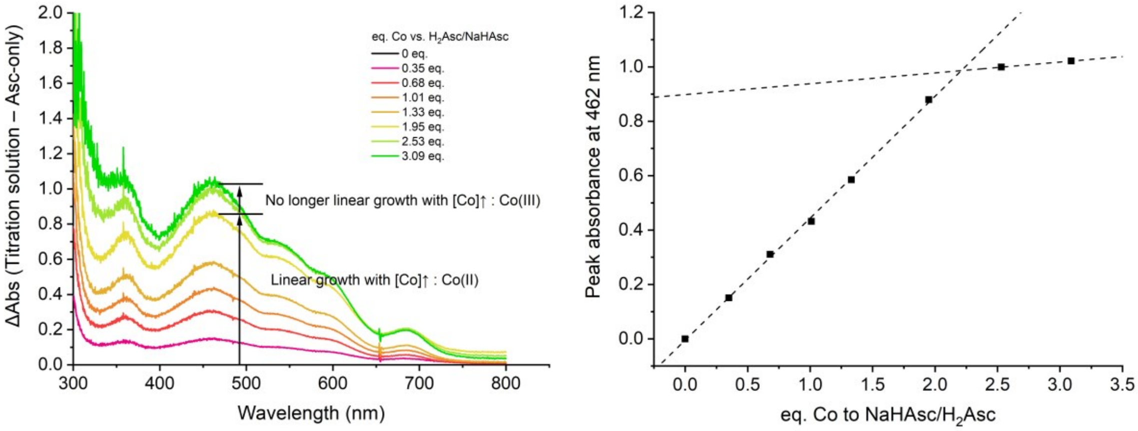

Figure S13. 'Reverse titration' experiment with gradual addition of a solution of 8.5 mM Co^{III} **1** in ascorbate buffer (pH = 4.5, 0.22 mM NaHAsc, 0.10 mM H_2Asc) into a solution of only ascorbate buffer (same concentrations for constant NaHAsc/H_2Asc total concentration). Performed in glovebox (oxygen-free) conditions. **Left**: Absorption spectra with ascorbate buffer. The absorbance baseline was subtracted for clarity. **Right**: Plots of the peak absorbance at 462 nm (for Co^{II} **1**). Dashed lines represent linear fits of the first six data points versus linear fits of the points from 2 eq. of catalyst to NaHAsc/H_2Asc onwards.

5.7 Titration Experiments with all Photocatalytic System Components

In titration experiments in the presence of CIS QDs (Figures S14–15) some broadened rising peaks which can be attributed to the formation of the Co^{II} form of each catalyst (*c.f.* Figure S11) can be identified: For **1**, at approximately 450 nm and 690 nm, while the 540 nm and 600 nm peaks visible in the QD-free samples appear to merge to a single peak at 580 nm; For **2**, at approximately 460 nm and with expected peaks around 550 nm, 600 nm and 700 nm appearing as a broad shoulder. At *ca.* 580 nm, Figures S14 and S15 show a growth in the Co^{II} absorbance to roughly half that expected without QDs. In order to get a sufficient absorption signal, however, titrations were done with much higher concentrations of catalyst than in the photocatalysis, PL quenching and fs-TA experiments. Nevertheless, it is clear that the conversion to Co^{II} state of the catalyst is not quantitative in the presence of QDs: Comparing the maximum increase in absorption at around 580 nm in the presence of QD to the difference in molar absorptivities of each oxidation state for both catalysts and accounting for the different total catalyst concentration, we estimate that only *ca.* 60% of either catalyst (61% of **1**, 58% of **2**) could be reduced in the presence of QDs, even with excess ascorbate (>> 0.5 eq.).

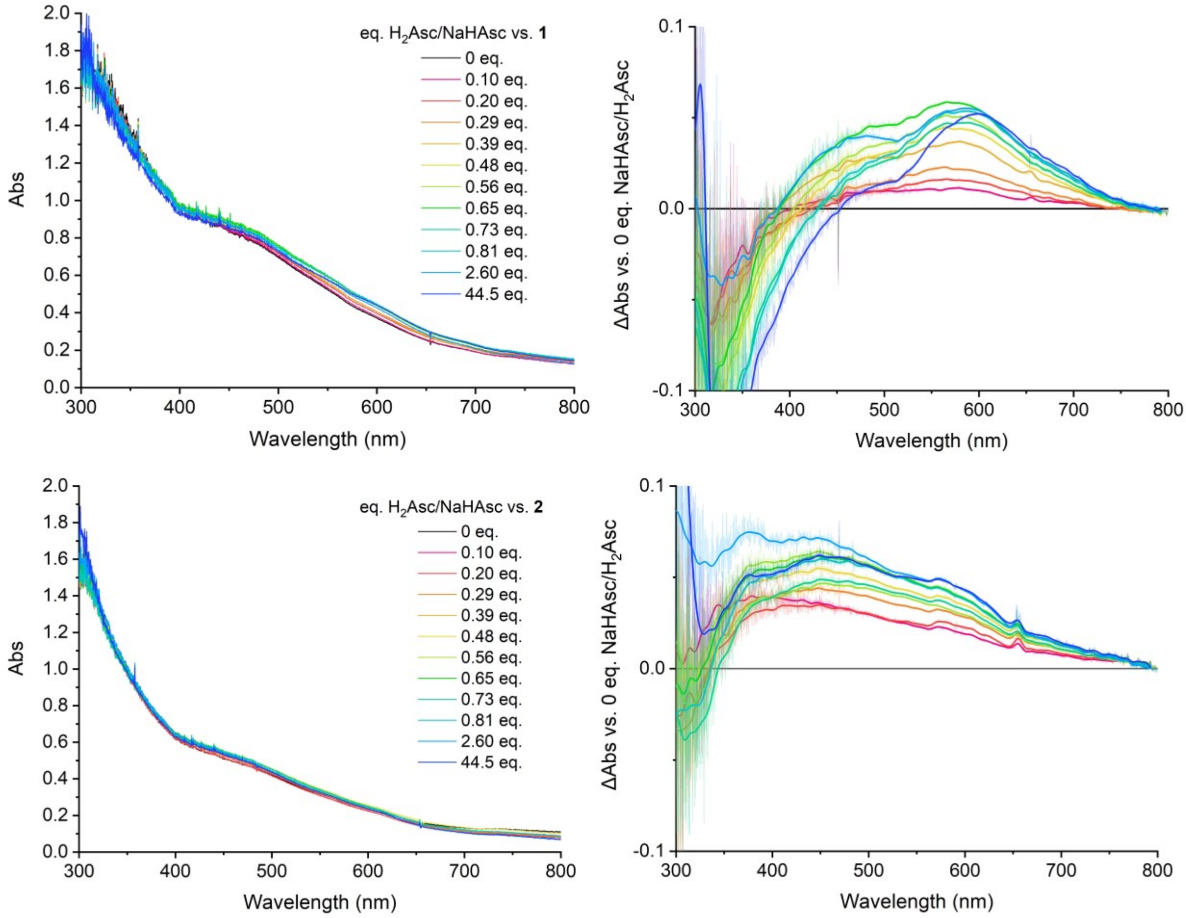

Figure S14. Titration absorption spectra from gradual addition of **1** (top) or **2** (bottom) in ascorbate buffer with CIS QDs to a solution of catalyst and QDs without ascorbate. Performed in glovebox (oxygen-free) conditions. Left: Raw absorbance spectra. Right: Change in absorbance versus the no-ascorbate initial solution, re-zeroed at 800 nm and smoothed to reduce instrumental baseline fluctuation. Constant [Co] of 144 μM and 86.4 μM and constant $[QD]_{rel.}$ of 5.7 μM and 3.4 μM (estimated from initial absorbance) for the **1** and **2** experiments, respectively: *i.e.* roughly 25 equivalents of catalyst per QD. Ascorbate buffer concentrations: 0.75 mM NaHAsc and 0.35 mM H_2Asc for **1** experiment; 0.45 mM NaHAsc and 0.21 mM H_2Asc for **2** experiment, respectively. 'Excess' ascorbate is 44.5 equivalents per Co.

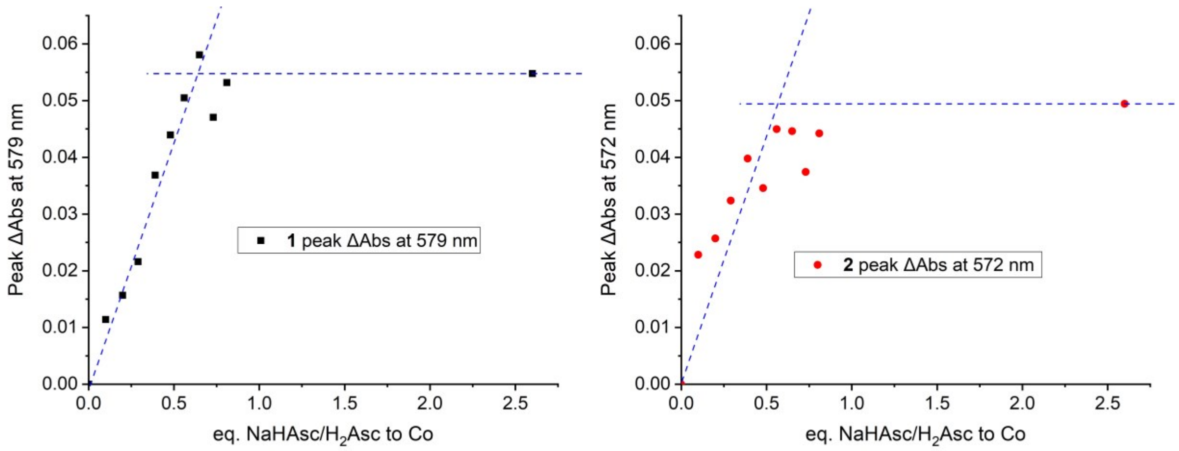

Figure S15. Plots of the change in peak absorbance versus the no-ascorbate control (at 579 nm for **1** and 572 nm for **2**) from Figure S14, with increasing ascorbate concentration under conditions of constant catalyst and QD concentrations. Dashed lines in blue represent a guide to the eye of the linear growth region followed by the saturated region at the ΔAbs corresponding to the titration endpoint with excess ascorbate. Changes appear to end between 0.50–0.75 eq. of ascorbate to cobalt, however the overall behavior is more complex than for the binary system without QDs.

5.8 Photoluminescence Quenching with Catalysts without O_2

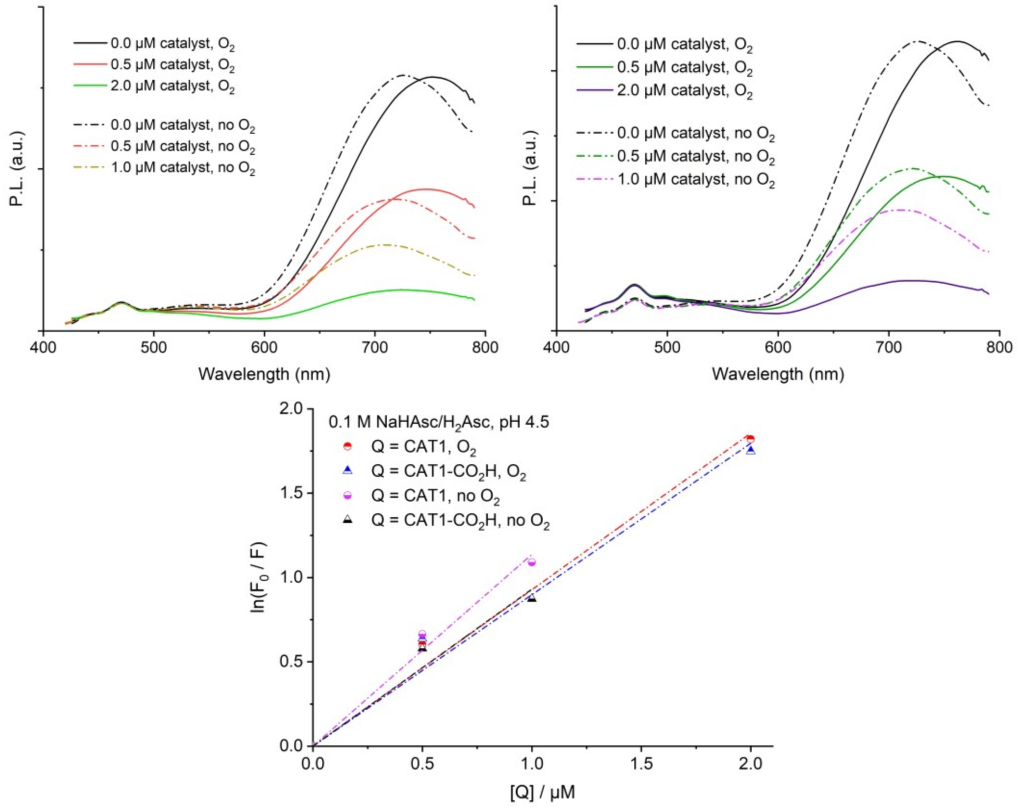

Figure S16. Top: Normalized PL spectra of the CIS QD system with **1** (left) or **2** (right) for a QD concentration corresponding to an absorbance of 0.07 at 405 nm in a 1 cm cuvette, estimated to be approximately 1.3 µM. Solvent: 0.1 M $NaHAsc/H_2Asc$, pH = 4.5. Atmospheric conditions (with oxygen) compared to glovebox conditions (oxygen-free). Bottom: Linearized PL ratios, $\ln(F_0/F)$, against the concentration of catalyst. Note that these experiments were performed on different batches of QDs, hence slightly different emission maxima.

6. Femtosecond Transient Absorption (fs-TA)

6.1 Fs-TA of CIS Quantum Dots

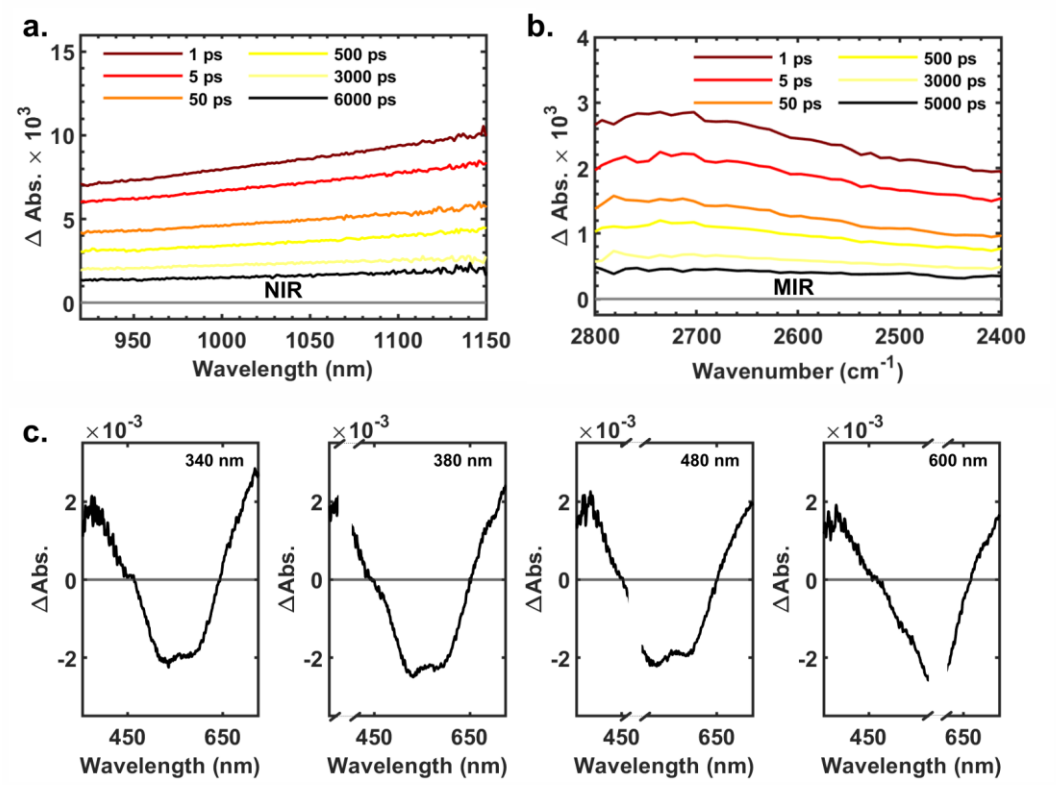

Figure S17. Transient absorption spectra of CIS quantum dots in water at varying pump-probe time-delays measured in the **a)** NIR and **b)** MIR using a 400 nm pump. The subplots in **c)** show the difference spectra at 20 ps pump-probe delay with pump wavelengths ranging from 340 nm (left) to 600 nm (right). The white cut-outs indicate the different pump wavelengths used (pump scattering).

6.2 Fs-TA of CIS QDs with Catalysts

The [Co]/$[QD]_{rel.}$ ratio in the data presented in the main article (Figure 7) and Figure S18-21 was *ca.* 0.7 and 0.6 for the UV-Vis and MIR measurements, respectively, as estimated from PL quenching modeling; see main article. The data was fitted using a sum of either three or four exponential terms, with an additional infinity component (offset) to account for the PIA from the reduced catalyst superimposed on the bleach band in the UV-Vis. Representative results from selected wavelengths are shown in Table S8. The obtained $<\tau>$ and ET rate constants are rather insensitive to the number of exponential terms used and whether the time components are fixed or kept free.

Table S8. Fit parameters and ET rate constants obtained from multiexponential fits.[a]

UV-Vis (525 nm)	τ_1 (A_1) [ps]	τ_2 (A_2) [ps]	τ_3 (A_3) [ps]	τ_4 (A_4) [ps]	$<\tau>^b$ [ps]	$<k_{ET}>^c$ [ps^{-1}]	$<\tau_{ET}>^c$ [ps]
**CIS**	1.8 (35 %)	28 (33 %)	610 (23 %)	4700 (8.1 %)	33	-	-
**CIS/1**	1.3 (47 %)	19 (35 %)	270 (15 %)	4400 (4.1 %)	10	0.070	14
**CIS/2**	0.90 (39 %)	11 (35 %)	170 (20 %)	4100 (6.6 %)	11	0.065	15
**CIS**	2.2 (39 %)	42 (34 %)	1300 (27 %)	-	33	-	-
**CIS/1**	1.3 (48 %)	21 (37 %)	430 (15 %)	-	8.6	0.085	12
**CIS/2**	1.2 (45 %)	20 (37 %)	430 (18 %)	-	9.5	0.074	13
**MIR (4000 nm)**	τ_1 (A_1) [ps]	τ_2 (A_2) [ps]	τ_3 (A_3) [ps]	τ_4 (A_4) [ps]	$<\tau>^b$ [ps]	$<k_{ET}>^c$ [ps^{-1}]	$<\tau_{ET}>^c$ [ps]
**CIS**	1.9 (27 %)	19 (37 %)	220 (23 %)	4200 (12 %)	35	-	-
**CIS/1**	0.86 (40 %)	8.5 (32 %)	110 (20 %)	4200 (8.7 %)	9.7	0.074	14
**CIS/2**	0.82 (31 %)	5.8 (34 %)	88 (24 %)	5000 (11 %)	13	0.049	20
**MIR (3595 nm)**	τ_1 (A_1) [ps]	τ_2 (A_2) [ps]	τ_3 (A_3) [ps]	τ_4 (A_4) [ps]	$<\tau>^b$ [ps]	$<k_{ET}>^c$ [ps^{-1}]	$<\tau_{ET}>^c$ [ps]
**CIS**	2.1 (32 %)	22 (36 %)	260 (21 %)	4000 (11 %)	31	-	-
**CIS/1**	0.84 (41 %)	8.6 (32 %)	120 (19 %)	4200 (8.4 %)	9.2	0.076	13
**CIS/2**	1.0 (36 %)	10 (37 %)	190 (16 %)	4900 (10 %)	13	0.043	23
**CIS**	3.5 (41 %)	66 (42 %)	2500 (17 %)	-	36	-	-
**CIS/1**	1.6 (51 %)	34 (36 %)	2600 (12 %)	-	12	0.058	17
**CIS/2**	2.0 (52 %)	42 (34 %)	2900 (14 %)	-	16	0.037	27
**CIS**[d]	3.5 (41 %)	66 (42 %)	2500 (17 %)	-	36	-	-
**CIS/1**[d]	1.5 (51 %)	33 (37 %)	[f]2500 (12 %)	-	12	0.058	17
**CIS/2**[d]	1.9 (51 %)	37 (34 %)	[f]2500 (15 %)	-	15	0.038	26

[a]Additional fits of the data shown in Figure 7 in the main article with an average catalyst number $\overline{n}$ = [Co]/[QD]$_{rel.}$ of 0.7 in the UV-Vis ([Co] = 50 µM) and 0.6 in the MIR ([Co] = 70 µM). [b]The amplitude weighted average $<\tau>$ were calculated from eq. 3 and [c]the average electron transfer rate constants and lifetimes were calculated from eq. 5 (see equations in main article).[d]The bottom row shows an example where τ_3 in the QD-catalyst systems were fixed to the value obtained from the CIS QD fits; no major difference was observed in the calculated parameters.

The results from fitting the fs-TA data with eq. S4 are shown in Table S9 and Figure S18. The first term accounts for the relative contribution from QDs with no catalyst bound (n = 0) assuming a scaling factor of exp($-\overline{n}$) according to the Poisson distribution. The dynamics of the free-QD fraction should be independent of the catalyst and the fit parameters were thus kept fixed to that of the catalyst-free measurements, ΔA_{QD}(t). The amplitude weighted average $<\tau>$ and ET rate constants could then be estimated from the second term accounting for the QDs with $n \geq 1$.

$$\Delta A(t) = \Delta A_{QD}(\mathrm{t}) \times \exp(-\overline{n}) + \sum_i A_i exp(-(k_i + k_{ET})\mathrm{t}) \quad (S4)$$

A_i and τ_i is the relative amplitude and lifetime of the ith component from the second term in eq. S4. The estimated ET rate constants are slightly larger than from the simple sum of exponentials presented in the main article and Table S8, with an $<k_{ET}>$ skewed towards higher catalyst numbers of the distribution and thus overestimate k_{ET} at n = 1. We note that the $n \geq 1$ fraction in the MIR data can be adequately fitted with a biexponential fit and yield slightly larger $<k_{ET}>$ compared to the UV-Vis data which is best fitted with three exponents to avoid oscillating residuals and minimize the SD (see Figure S18). From the similar $\overline{n}$ used in the experiments, however, we would expect similar distributions of rates from QD populations with $n \geq 1$ in both cases. The observed disparities may thus arise from the reduced catalyst absorption (Co^{II}) in the UV-Vis which was treated with an additional infinity component (ignored in the average).

Table S9. Fit parameters and ET rate constants accounting for the QDs without catalyst (n = 0).[a]

UV-Vis (530 nm)	τ_1 (A_1) [ps]	τ_2 (A_2) [ps]	τ_3 (A_3) [ps]	$<\tau>$[b] [ps]	$<k_{ET}>$[c] [ps^{-1}]	$<\tau_{ET}>$[c] [ps]	$\bar{n}_{fit.}$[d] [ps]
CIS/1 (biexp.)	1.5 (57 %)	32 (43 %)	-	5.5	0.16	6.4	0.72
CIS/2 (biexp.)	1.6 (56 %)	32 (44 %)	-	5.8	0.15	6.8	0.57
CIS/1 (triexp.)	0.47 (30 %)	4.5 (43 %)	56 (27 %)	2.3	0.41	2.4	0.74
CIS/2 (triexp.)	0.40 (29 %)	4.8 (44 %)	66 (27 %)	2.3	0.40	2.5	0.63
**MIR (3800 nm)**	**τ_1 (A_1) [ps]**	**τ_2 (A_2) [ps]**	**τ_3 (A_3) [ps]**	**$<\tau>$[b] [ps]**	**$<k_{ET}>$[c] [ps^{-1}]**	**$<\tau_{ET}>$[c] [ps]**	**$\bar{n}_{fit.}$[d] [ps]**
CIS/1 (biexp.)	0.41 (41 %)	3.7 (59 %)	-	1.3	0.76	1.3	0.61
CIS/2 (biexp.)	0.29 (25 %)	2.3 (75 %)	-	1.4	0.73	1.4	0.44

[a]Alternative fit parameters obtained for the same samples as in Table S8 and in Figure 7 (main text) but instead fitted to eq. S4 with the [b]amplitude weighted average $<\tau>$ calculated from eq. 3 in the main text and the [c]average ET rate constants as well as lifetimes from eq. 5. [d] The $\bar{n}_{fit.}$ are close to the average catalyst numbers estimated from the PL quenching: $\bar{n}$ = $[Co]/[QD]_{rel.}$ of *ca.* 0.7 (UV-Vis) and 0.6 (MIR).

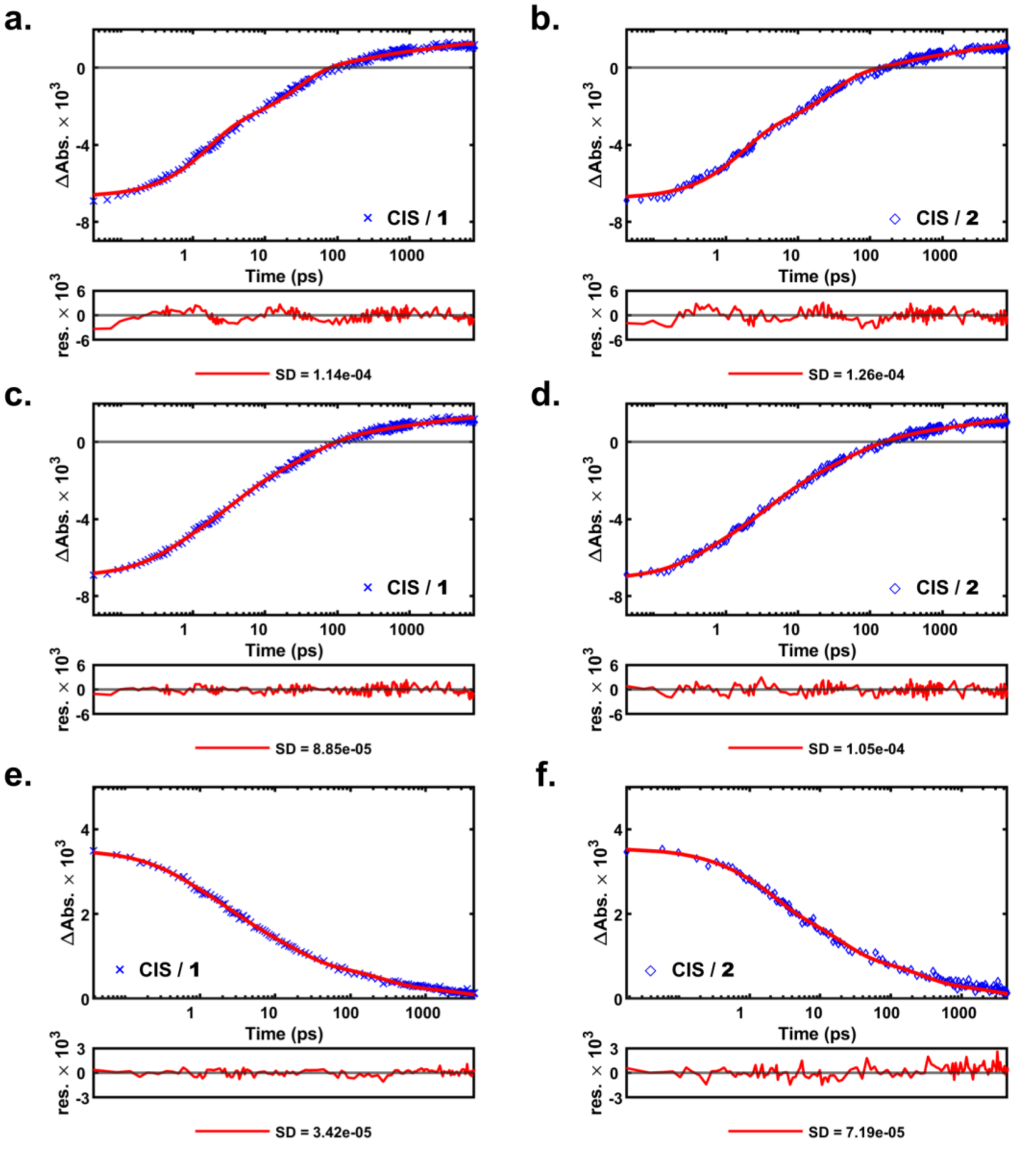

Figure S18. Example of kinetic traces fitted through eq. S4 with the corresponding fit parameters shown in Table S9. The UV-Vis data fitted with **a-b)** a biexponential, and **c-d)** a triexponential in the second term of eq. S4. **e-f)** MIR data fitted with two exponential terms. SD is the standard deviation.

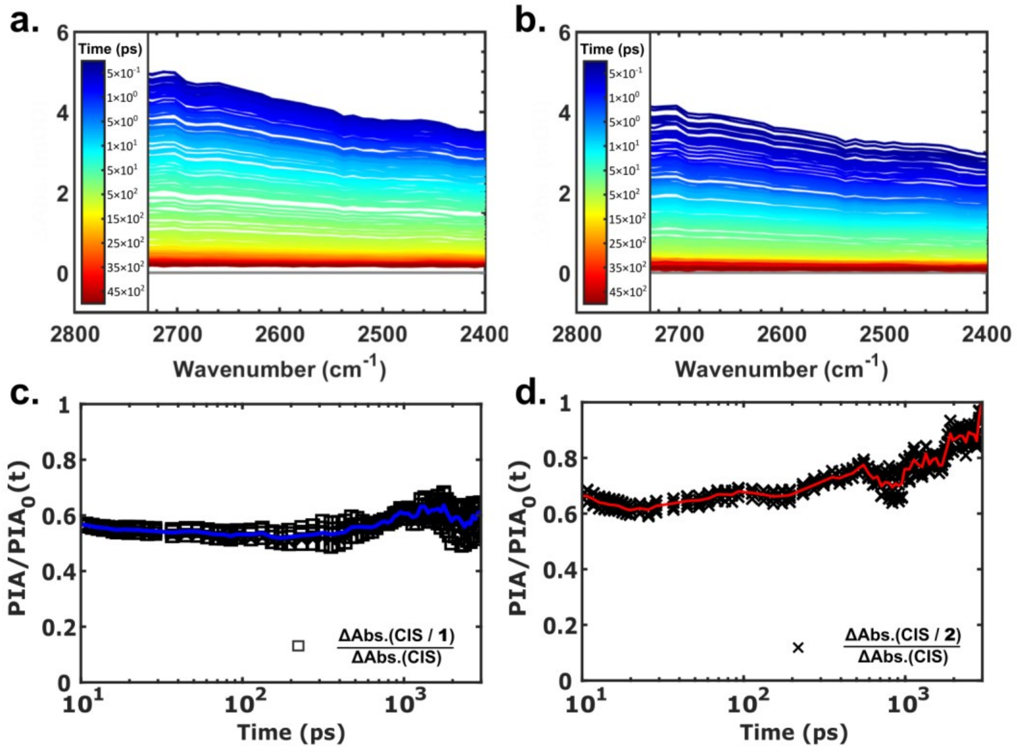

Figure S19. Transient absorption experiments probing in the MIR (pump: 400 nm, *ca.* 20 μJ/cm^2). Difference spectra of **a)** CIS quantum dots, and **b)** CIS quantum dots with **1** at a [**1**]/[QD]$_{rel.}$ ratio of $\overline{n}$ ≈ 0.6 at time-delays ranging from 0.5 ps (dark blue) to 4.5 ns (dark red). The samples were prepared in an ascorbate buffer (H_2Asc/NaHAsc, pH = 4.5). The PIA ratios (PIA/PIA$_0$) over the range of 10 ps to 3.5 ns for **c)** CIS/**1** and **d)** CIS/**2**. The kinetic traces were averaged over five wavenumbers. The solid lines represent the moving average.

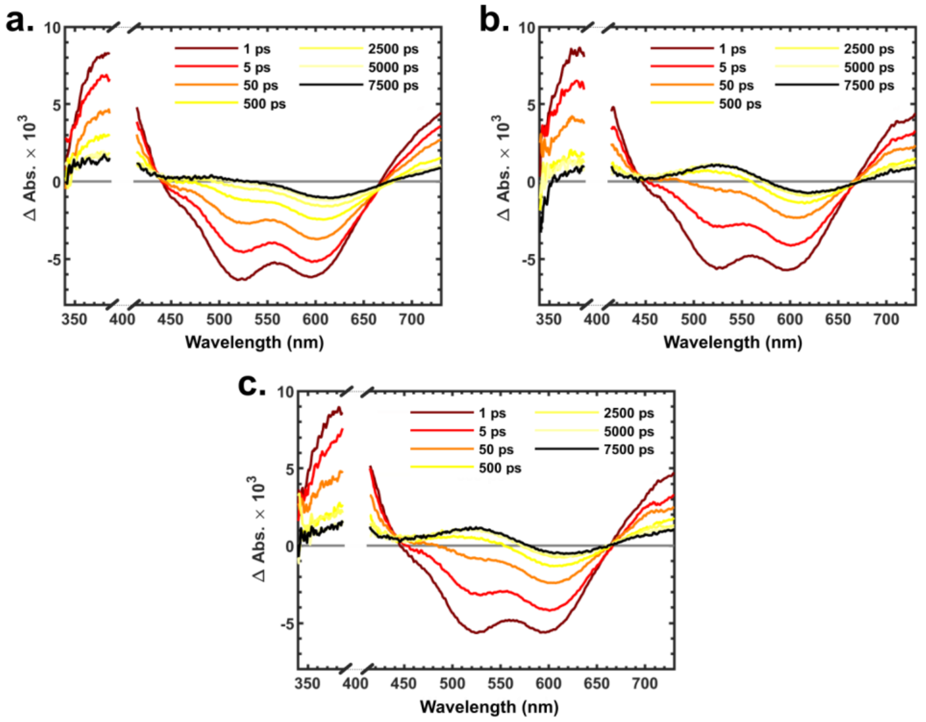

Figure S20. Transient absorption spectra (pump: 400 nm, *ca.* 40 μJ/cm^2) of **a)** CIS quantum dots in ascorbate buffer (H_2Asc/NaHAsc, pH = 4.5, 0.1 M) and with the addition of *ca.* 0.7 equivalents ([Co]/[QD]$_{rel.}$) of **b) 1** and **c) 2** at various time delays (insets). The scattering from the pump has been removed from the spectra for clarity (white cut-out).

The CIS QDs in water (unbuffered) show a slower B1 bleach recovery (~525 nm) compared to buffered samples ($H_2Asc/NaHAsc$, pH = 4.5) and a different B1/B2 bleach ratio at t = 0. Upon addition of *ca.* 0.7 equivalents of catalyst (either **1** or **2**), the TA spectral response hence differs (the absorption from the reduced catalyst gives less net positive ΔAbs. in the buffer-free samples, Figure S21 (a) under otherwise identical conditions. However, the ΔΔAbs. ($\Delta A_{QD/CAT} - \Delta A_{QD}$; corrected for the QD response *w/wo.* buffer) at 8 ns pump-probe delay in Figure S21 (b) show nearly identical features suggesting that the dominating ET product is Co^{II} in both cases.

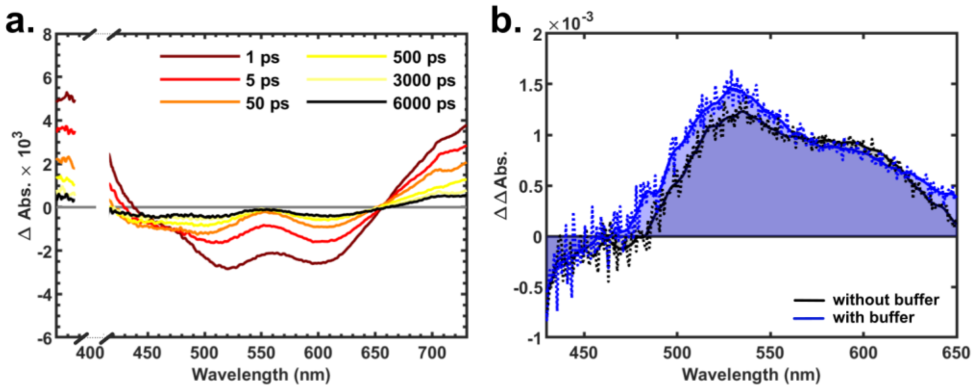

Figure S21. Transient absorption measurements of CIS/**1** with an average catalyst number $\overline{n}$ = [**1**]/$[QD]_{rel.}$ of *ca.* 0.7 ([**1**] = 50 µM). **a)** Difference spectra (ΔAbs.) of unbuffered CIS/**1**, and **b)** ΔΔAbs. ($\Delta A_{QD/CAT} - \Delta A_{QD}$) of CIS/**1** at 8 ns in the presence (shaded blue) and absence (shaded black) of ascorbate buffer ($H_2Asc/NaHAsc$, pH = 4.5, 0.1 M).

6.3 Fs-TA of CIS Quantum Dots with Methyl Viologen

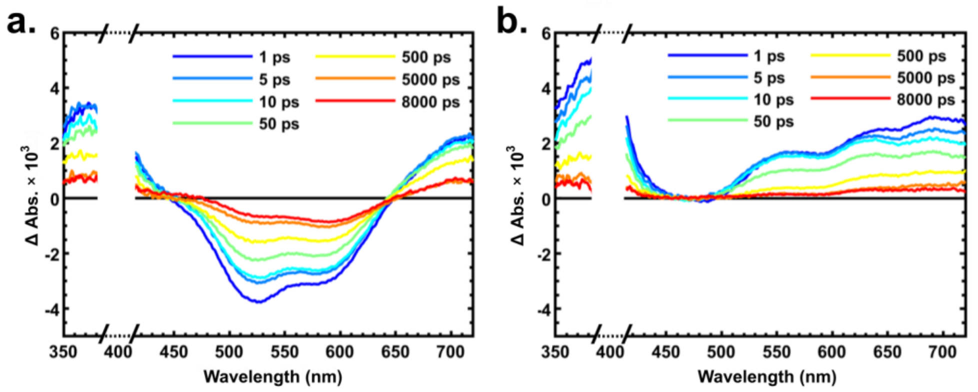

Figure S22. Transient absorption measurements (pump: 400 nm) of **a)** CIS QDs in water, and **b)** CIS with ~2.9 equivalents of methyl viologen (MV^{2+}). Note that the positive transient (PIA) from the $MV^{\bullet+}$ radical cation formed upon electron transfer from the photoexcited QDs is superimposed on the remaining QD signals.

6.4 Fs-TA of CIS Quantum Dots with Varying [**1**]

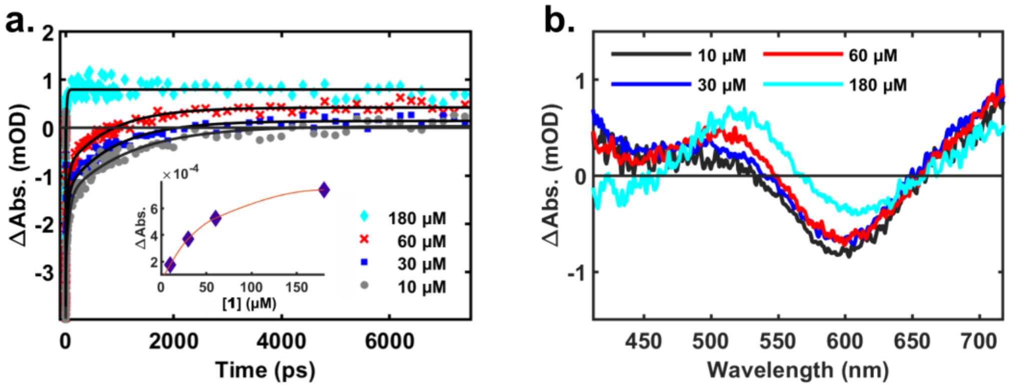

Figure S23. Transient absorption measurements of CIS quantum dots mixed with **1** (10–180 µM; $\overline{n}$ = 0.1–2 from [**1**]/$[QD]_{rel.}$) in 0.1 M H_2Asc/NaHAsc buffer (pH = 4.5). **a)** Kinetics extracted at 520 nm. The inset shows the increase in ΔA. from Co^{II} formation upon electron transfer from the photoexcited CIS quantum dots. **b)** Transient spectra averaged over 7-8 ns. All samples were prepared in a glovebox to mimic the conditions used in the oxygen-free measurements before being exposed to atmospheric oxygen for *ca.* 1 h prior to measurement. The samples were pumped at 400 nm and measured with a 1 mm pathlength.

6.5 Fs-TA of CIS/**1**/$HAsc^-$ Samples in the Absence and Presence of Oxygen

The effect of oxygen on the TA spectra and kinetics was small (Figure S24-S25) and mainly affected the level of remaining 520 nm-signal at longer pump-probe time delays, attributed to the Co^{II} state of the catalyst. The samples free of oxygen show no features that can be associated with Co^{I} even at lower catalyst/QD ratios (Figure S24: left).

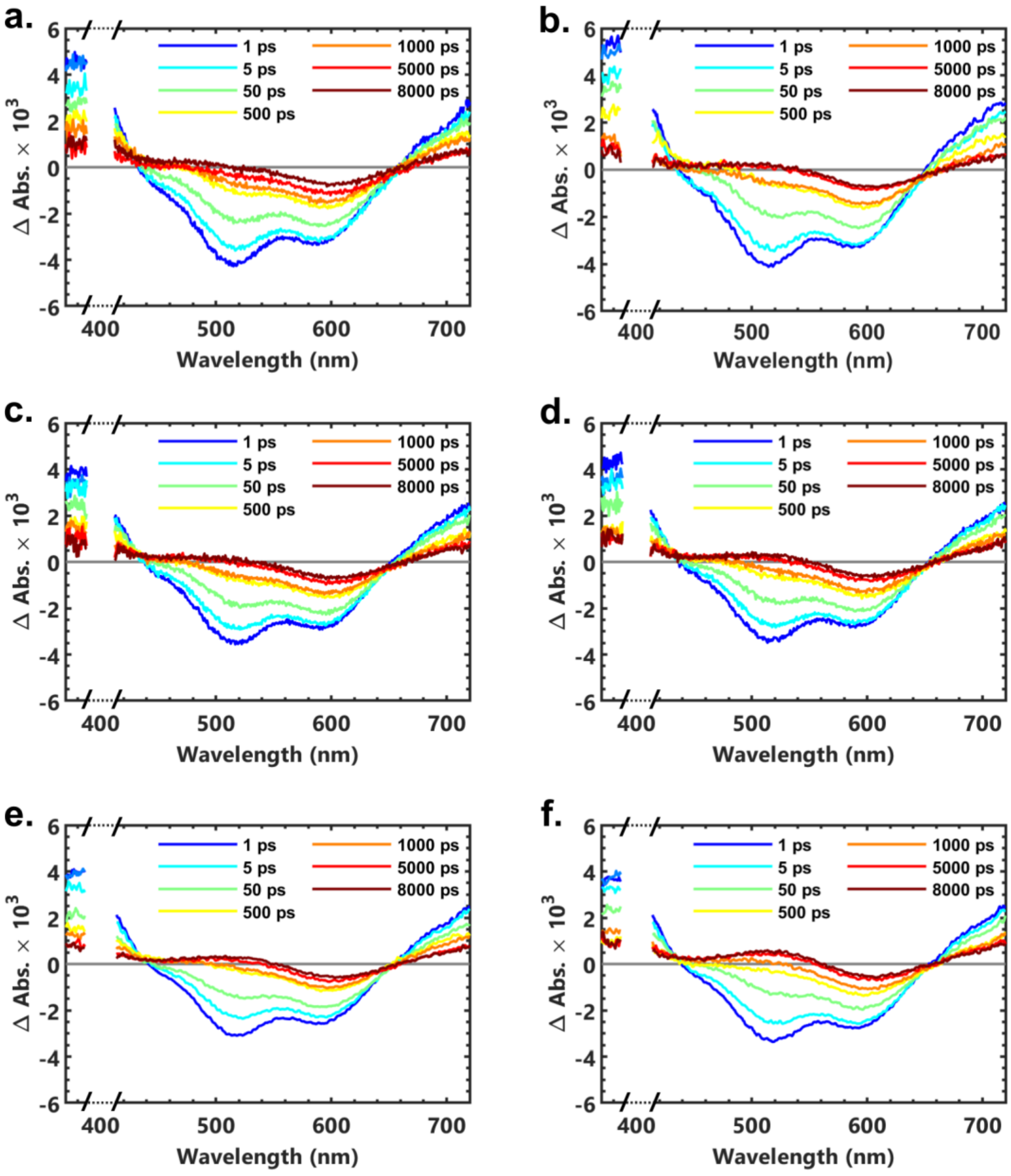

Figure S24. Transient absorption measurements of CIS quantum dots mixed with **1** in 0.1 M H_2Asc/NaHAsc buffer (pH = 4.5) in the absence (left panels) and presence (right panels) of oxygen. All samples were prepared and sealed in a glovebox with [**1**]/[QD]$_{rel.}$ ratios of **a,b)** ~0.11 ([**1**]=10 µM), **c,d)** ~0.32 ([**1**]=30 µM), and **e,f)** ~0.65 ([**1**]=60 µM). All samples were prepared in a glovebox, however, the samples in the right panels were exposed to atmospheric oxygen for *ca.* 1 h prior to measurement.

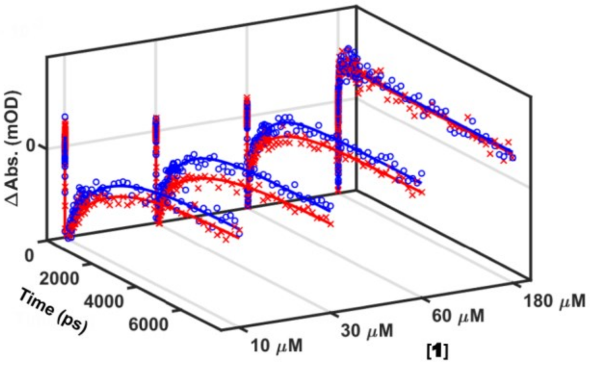

Figure S25. Kinetic traces extracted at 520 nm from transient absorption measurements of CIS quantum dots mixed with **1** (10–180 μM; $\overline{n}$ = 0.1–2 from [**1**]/[QD]$_{rel.}$) in 0.1 M $H_2Asc/NaHAsc$ buffer (pH = 4.5) with oxygen present (blue) and in the absence of oxygen (red). All samples were prepared and sealed in a glovebox. The samples with oxygen were exposed to atmospheric oxygen for *ca.* 1 h prior to measurement.

7. Synthesis and Characterization of Catalyst 2

7.1 Synthesis of 2

It was previously remarked in literature that preparing functionalized derivatives of **1** involves some synthetic challenges, particularly regarding the macrocyclization step in the cases where a derivative of diacetylpyridine or norspermidine with different solubilities is employed.[3] Therefore, some new alterations were made to the solvent system for macrocyclization, adding 1,4-dioxane as a co-solvent to aid solubility.

7.1.1 Synthesis of tert-Butyl-4-(2,6-diacetyl-pyridin-4-yl)benzoate

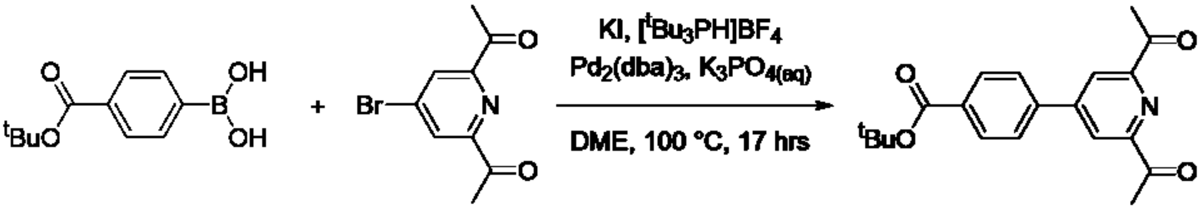

Scheme S1: Synthesis of tert-butyl-4-(2,6-diacetyl-pyridin-4-yl)benzoate from 4-bromo-2,6-diacetyl pyridine and 4-(tert-butoxycarbonyl)phenylboronic acid via a thermally activated Suzuki reaction.

Note that for this reaction emphasis is placed upon the oxygen-free conditions to maximize yield and minimize homocoupled side-products.

Method: A high pressure tolerant Schlenk vessel with a capacity greater than 80 mL to be used as the reaction vessel was evacuated and flushed with argon three times and left containing an atmosphere of argon gas and the magnetic stirring bar. The solids 4-bromo-2,6-diacetylpyridine (1500 mg, 6.20 mmol), 4-(*tert*-butoxycarbonyl)phenylboronic acid (1380 mg, 6.20 mmol, 1.00 eq.), oven-dried potassium iodide (2440 mg, 14.7 mmol, 2.37 eq.), and tri-*tert*-butylphosphonium tetrafluoroborate (197 mg, 0.68 mmol, 0.11 eq.) were poured one at a time into the reaction vessel (with argon inflow stopped to avoid blowing the powders out). Each insertion was followed by a brief careful additional purge. Following this, pre-purged dimethoxyethane (DME) (62 mL) was quickly inserted to give an orange solution. Lastly, the catalyst tris(dibenzylideneacetone)dipalladium (197 mg, 0.22 mmol, 0.03 eq.) was poured in to give a black solution. Heating to 65 °C was initiated. Meanwhile, K_3PO_4 (3340 g, 15.8 mmol, 2.54 eq.) was dissolved thoroughly in deionized, pre-purged water (16 mL), taking care to manage the heat produced, and the solution was purged for several further minutes before insertion at a moderate rate into the reaction vessel under argon inflow. The reaction vessel was sealed and the mixture was left stirring at 100 °C for 18 hours. After this time, the reaction mixture was allowed to cool, separating into distinct organic and aqueous phases; the top DME layer was dark and opaque, whereas the bottom water layer was slightly cloudy. The mixture was transferred to a large round-bottomed flask, washing with DCM, and the stirring bar was removed. The solvents were removed by rotary evaporation (DME causes issues in the following separation). The dark-brown, sticky crude product was then transferred to a separating funnel, washing through with DCM (150 mL) and aqueous KCl solution (150 mL, 10% saturated), made up by mixing 15 mL of saturated aqueous KCl solution and 135 mL of deionized water. The organic layer (bottom) was dark brown; the aqueous layer (top) was pale-cloudy. Some undissolved black-purple particles were present (suspected $Pd_2(dba)_3$). The organic layer was extracted, and the aqueous layer was further washed with DCM (2 × 150 mL). The organic layers were combined, washed with brine (150 mL), dried with $MgSO_4$ and filtered on a large silica frit into large round-bottomed flask. The solvent was removed by rotary evaporation to leave a brown-orange solid.

The crude product was purified by flash column chromatography (Stationary phase: 2 x 40 g silica gel (pore size 30 µm) *Interchim*® flash columns, packed with 4 column volumes of toluene (note that a single 80 g column would be superior). The crude product was dry loaded (found to be superior to wet

loading): Silica (approx. 20 g, pore size 60 μm) was added to the flask, followed by DCM to dissolve the crude product. The crude product and silica were mixed together, then DCM was removed by rotary evaporation (with a cotton wool plug in the adaptor) to leave a pale brown powder, which was inserted into a cartridge connected to the top of the column, gently tapping to achieve a level surface, covered with sand and sealed with the frit. Eluent: Initially 100% toluene with a gradual gradient to 1:1 toluene/chloroform, run at the recommended flow rate. Inbuilt UV-visible detectors and TLC were used to determine which fractions contained purified product (Stationary phase: aluminum-backed silica gel. Eluent: DCM *or* 1:1 toluene/chloroform). Product fractions were combined and dried by rotary evaporation, then direct vacuum to give *tert*-butyl-4-(2,6-diacetyl-pyridin-4-yl)benzoate as a fluffy white solid of high purity (1310 mg, 3.87 mmol, 63%). ^{1}H NMR ($CDCl_3$, 300 MHz): δ 8.47 (s, 2H), δ 8.12 (d, 2H, J = 8.4 Hz), δ 7.78 (d, 2H, J = 8.4 Hz), δ 2.84 (s, 6H), δ 1.63 (s, 9H). ^{13}C{^{1}H} NMR ($CDCl_3$, 300 MHz, proton numbers assigned by DEPT): δ 199.6 (**C**), δ 165.2 (**C**), δ 153.7 (**C**), δ 149.8 (**C**), δ 140.6 (**C**), δ 133.4 (**C**), δ 130.5 (**CH**), δ 127.2 (**CH**), δ 122.6 (**CH**), δ 81.8 (**C**), δ 28.3 (**CH_3**), δ 25.9 (**CH_3**).

7.1.2 Synthesis of [Co(N$_4$H-CO$_2$^{t}Bu)Cl$_2$]$^+$

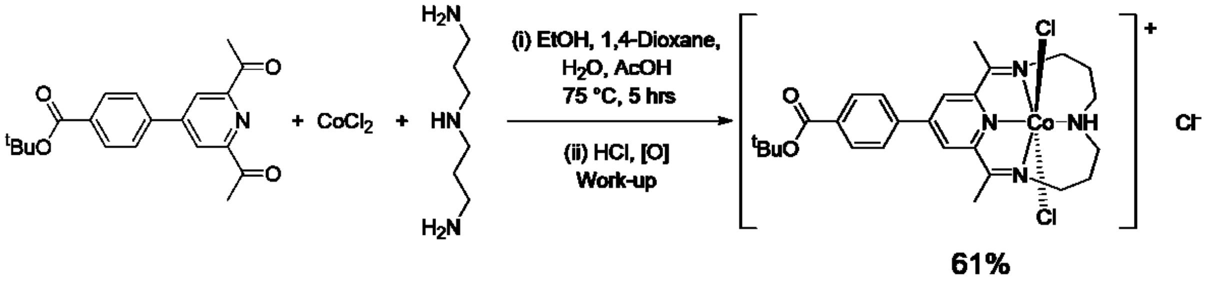

Scheme S2. Synthesis of **[Co(N$_4$H-CO$_2$^{t}Bu)Cl$_2$]$^+$Cl$^-$** from its tert-butyl-4-(2,6-diacetyl-pyridin-4-yl)benzoate and triamine ligand precursors as well as $CoCl_2$ via a templated macrocyclization reaction.

Method: *tert*-Butyl-4-(2,6-diacetyl-pyridin-4-yl)benzoate (340 mg, 1.00 mmol) was inserted into a pre-purged Schlenk tube containing an atmosphere of argon gas and the magnetic stirring bar. Pre-purged ethanol (6.8 mL) was inserted along the edge of the flask to wash the starting material to its base during argon inflow. No dissolution could be observed. Pre-purged 1,4-Dioxane (4.1 mL) was then inserted, causing partial dissolution to a turbid orange suspension. The reaction mixture was heated to 50 °C for 15 minutes to dissolve the substrate before a solution of cobalt dichloride hexahydrate (239 mg, 1.00 mmol) dissolved in pre-purged deionized water (2.0 mL) was inserted into the solution during argon inflow, causing a rapid colour change to blue. The reaction mixture was then allowed to heat further to 75 °C for 15 minutes before bis(3-aminopropyl)amine (136 μL, 128 mg, 0.98 mmol) was inserted dropwise under during argon inflow, causing rapid colour change to lighter blue, then purple. Finally, concentrated acetic acid (102 μL, 98%) was added dropwise under argon inflow, resulting in some fuming but no immediate colour change. The reaction mixture was then sealed and left stirring at 75 °C for 5 hours. After this time, the reaction vessel was placed in a water bath to cool. The mixture was then aerated by bubbling air into the solution overnight, from a pump through an ethanol trap so that evaporated solvent would be replaced. After 17 hours, concentrated aqueous HCl (238 μL, 37%) was added dropwise causing a colour change to brown. Aeration was continued for another hour with stirring.

Work-up: The crude product was transferred to a round-bottomed flask using water and ethanol. The stirring bar was removed before rotary evaporation to remove the solvents. The sticky solid was then redissolved in minimum water and the product was precipitated by adding excess saturated KCl aqueous solution. (Note: Dissolving the crude product in minimum water, then adding excess ethanol can be used to remove any remaining *tert*-butyl-4-(2,6-diacetyl-pyridin-4-yl)benzoate starting material if present.) The supernatant was removed and the remaining solid was dissolved in acetonitrile before excess diethyl ether was added in order to reprecipitate the product. This was then poured onto a silica

frit filter, transferring using excess diethyl ether and a tiny amount of tetrahydrofuran if necessary. After washing with excess diethyl ether, the product was washed through the filter using DCM until no more would pass through. Solvent was removed from the product by rotary evaporation then direct vacuum to leave $[Co(N_4H\text{-}CO_2{}^tBu)Cl_2]Cl^-$ as a dull green powder (365 mg, 61%). ^1H NMR (CD_3CN, 300 MHz): δ 8.63 (s, 2H), δ 8.22 (d, 2H, *J* = 8.5 Hz), δ 8.11 (d, 2H, *J* = 8.5 Hz), δ 6.03 (br t, 1H), δ 4.03 (d, 2H, *J* = 16.5 Hz), δ 3.58 (t, 2H, *J* = 14.7 Hz) δ 3.33 (q, 2H, *J* = 12.1 Hz), δ 2.94 (d, 2H, *J* = 12.3 Hz), δ 2.89 (s, 6H), δ 2.30 (d, 2H, *J* = 15.4 Hz), δ 2.09 (d, 2H, *J* = 12.3 Hz), δ 1.62 (s, 9H). $^{13}C\{^1H\}$ NMR (CD_3CN, 300 MHz, proton numbers assigned by DEPT): δ 179.3 (**C**), δ 165.7 (**C**), δ 158.2 (**C**), δ 154.0 (**C**), δ 140.3 (**C**), δ 135.3 (**C**), δ 131.1 (**CH**), δ 129.4 (**CH**), δ 127.0 (**CH**), δ 82.6 (**C**), δ 52.4 (**CH_2**), δ 50.8 (**CH_2**), δ 28.3 (**CH_3**), δ 27.0 (**CH_2**), δ 18.3 (**CH_3**).

7.1.3 Synthesis of $[Co(N_4H\text{-}CO_2H)Cl_2]^+Cl^-$

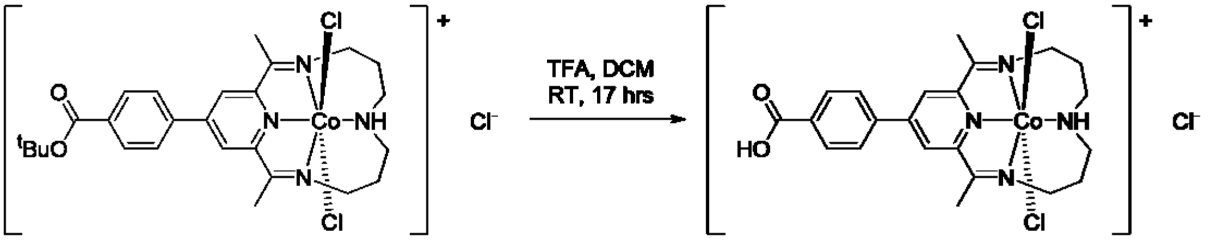

Scheme S3. Synthesis of **$[Co(N_4H\text{-}CO_2H)Cl_2]^+Cl^-$** by deprotection of the tert-butyl ester of **$[Co(N_4H\text{-}CO_2{}^tBu)Cl_2]^+Cl^-$** using trifluoroacetic acid.

Method: **$[Co(N_4H\text{-}CO_2{}^tBu)Cl_2]^+Cl^-$** (61.5 mg, 0.103 mmol) was placed into a 50 mL round-bottomed flask. DCM (8.0 mL) was added to dissolve the substrate with stirring. Once dissolution was complete, trifluoroacetic acid (480 µL, >98%) was carefully added dropwise. The reaction vessel was then sealed with a septum and covered with aluminum foil and the reaction mixture was left stirring at room temperature overnight. After 17 hours, the reaction mixture's appearance had darkened somewhat. The solvent and trifluoroacetic acid were removed by rotary evaporation, then the product was transferred to a sample vial with minimum DCM and dried under direct vacuum for many hours to leave **$[Co(N_4H\text{-}CO_2H)Cl_2]^+Cl^-$** as a viscous, dark green solid. After evaporation until the product is dry, quantification is difficult because in most cases the final product is very hygroscopic. Complete hydrolysis is determined by ^1H NMR. ^1H NMR (CD_3CN, 300 MHz): δ 8.62 (s, 2H), δ 8.27 (d, 2H, *J* = 7.9 Hz), δ 8.12 (d, 2H, *J* = 8.1 Hz), δ 6.00 (br t, 1H, *J* = 10.6 Hz), δ 4.02 (d, 2H, *J* = 16.3 Hz), δ 3.58 (t, 2H, *J* = 14.7 Hz), δ 3.33 (q, 2H, *J* = 12.1 Hz), δ 2.99 (d, 2H, *J* = 15.5 Hz), δ 2.88 (s, 6H), δ 2.31 (d, 2H, *J* = 15.8 Hz), δ 2.12 (d, 2H, *J* = 14.2 Hz). $^{13}C\{^1H\}$ NMR (CD_3CN, 300 MHz, proton numbers assigned by DEPT): δ 179.3 (**C**), δ 172.2 (**C**), δ 167.0 (**C**), δ 158.2 (**C**), δ 140.0 (**C**), δ 135.9 (**C**), δ 131.6 (**CH**), δ 129.5 (**CH**), δ 127.0 (**CH**), δ 95.7 (**C**), δ 52.4 (**CH_2**), δ 50.8 (**CH_2**), δ 27.0 (**CH_2**), δ 18.3 (**CH_3**).

7.2 Electrochemical Characterization

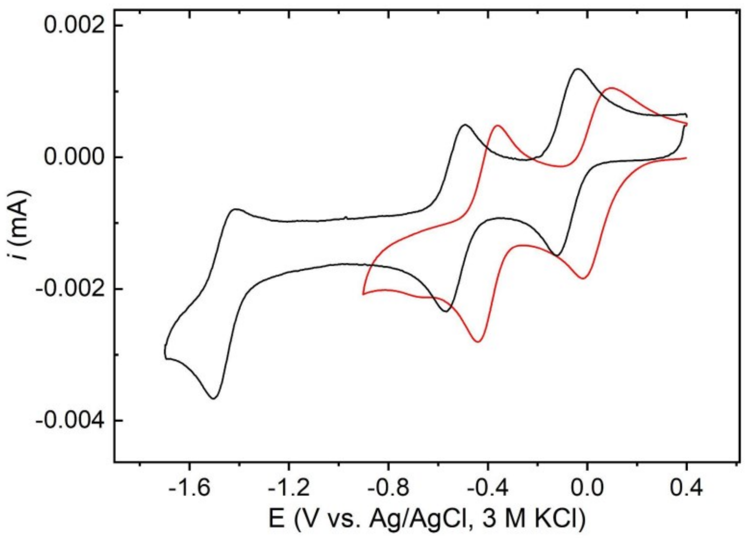

Figure S26. Cyclic voltammograms of 0.5 mM **1** (black) and **2** (red) in 0.1 M $TBAPF_6$/MeCN. Scan rate: 100 mV. The benzoic acid moiety shifts the $Co^{III/II}$ and $Co^{II/I}$ redox couples to more positive potentials. The $Co^{III/II}$ redox couple shows somewhat decreased reversibility.

8. References

(1) Huang, J.; Xu, B.; Tian, L.; Pati, P. B.; Etman, A. S.; Sun, J.; Hammarström, L.; Tian, H. A Heavy Metal-Free $CuInS_2$ Quantum Dot Sensitized NiO Photocathode with a Re Molecular Catalyst for Photoelectrochemical CO_2 Reduction. *Chem. Commun.* **2019**, *55* (55), 7918–7921.

(2) De Roo, J. Chemical Considerations for Colloidal Nanocrystal Synthesis. *Chem. Mater.* **2022**, *34* (13), 5766–5779.

(3) Nie, C.; Ni, W.; Gong, L.; Jiang, J.; Wang, J.; Wang, M. Charge Transfer Dynamics and Catalytic Performance of a Covalently Linked Hybrid Assembly Comprising a Functionalized Cobalt Tetraazamacrocyclic Catalyst and $CuInS_2$/ZnS Quantum Dots for Photochemical Hydrogen Production. *J. Mater. Chem. A* **2019**, *7* (48), 27432–27440.

(4) Fan, X.-B.; Yu, S.; Zhan, F.; Li, Z.-J.; Gao, Y.-J.; Li, X.-B.; Zhang, L.-P.; Tao, Y.; Tung, C.-H.; Wu, L.-Z. Nonstoichiometric $Cu_x In_y S$ Quantum Dots for Efficient Photocatalytic Hydrogen Evolution. *ChemSusChem* **2017**, *10* (24), 4833–4838.

(5) Sandroni, M.; Gueret, R.; Wegner, K. D.; Reiss, P.; Fortage, J.; Aldakov, D.; Collomb, M.-N. Cadmium-Free $CuInS_2$/ZnS Quantum Dots as Efficient and Robust Photosensitizers in Combination with a Molecular Catalyst for Visible Light-Driven H_2 Production in Water. *Energy Environ. Sci.* **2018**, *11* (7), 1752–1761.

(6) National Renewable Energy Laboratory (NREL). *Solar Resource Data and Tools. Reference Air Mass 1.5 Spectra*. Solar Spectra | Grid Modernization | NREL. https://www.nrel.gov/grid/solar-resource/spectra-am1.5.html (accessed 2024-01-31).

(7) Orlando, A.; Lucarini, F.; Benazzi, E.; Droghetti, F.; Ruggi, A.; Natali, M. Rethinking Electronic Effects in Photochemical Hydrogen Evolution Using $CuInS_2$@ZnS Quantum Dots Sensitizers. *Molecules* **2022**, *27* (23), 8277.

(8) Hansson, S. Photophysical Investigation of $CuInS_2$ Quantum Dots with Molecular Acceptors of Different Charge (Dissertation), Uppsala University, Uppsala, 2023. https://urn.kb.se/resolve?urn=urn:nbn:se:uu:diva-506304.
